# DNA methylation and expression of *MAPRE3* affect overall survival of early‐stage non‐small cell lung cancer patients

**DOI:** 10.1002/1878-0261.70260

**Published:** 2026-04-25

**Authors:** Chao Chen, Jiancheng Cheng, Ruili Hou, Xiaoyuan Zheng, Li Su, Maria Moksnes Bjaanæs, Anna Karlsson, Maria Planck, Johan Staaf, Åslaug Helland, Manel Esteller, David C. Christiani, Feng Chen, Xiaofei Cao, Ruyang Zhang

**Affiliations:** ^1^ Department of Biostatistics, Center for Global Health, School of Public Health Nanjing Medical University Jiangsu China; ^2^ Information Center, The Second People's Hospital of Changzhou, The Third Affiliated Hospital of Nanjing Medical University Jiangsu China; ^3^ Department of Environmental Health Harvard T.H. Chan School of Public Health Boston MA USA; ^4^ Department of Cancer Genetics Institute for Cancer Research, Oslo University Hospital Norway; ^5^ Division of Oncology, Department of Clinical Sciences Lund and CREATE Health Strategic Center for Translational Cancer Research Lund University Sweden; ^6^ Institute of Clinical Medicine University of Oslo Norway; ^7^ Cancer Epigenetics Group Josep Carreras Leukaemia Research Institute (IJC) Barcelona Spain; ^8^ Centro de Investigación Biomédica en Red Cancer (CIBERONC) Madrid Spain; ^9^ Institució Catalana de Recerca i Estudis Avançats (ICREA) Barcelona Spain; ^10^ Physiological Sciences Department, School of Medicine and Health Sciences University of Barcelona Spain; ^11^ Pulmonary and Critical Care Division, Department of Medicine, Massachusetts General Hospital and Harvard Medical School Boston USA; ^12^ China International Cooperation Center for Environment and Human Health Nanjing Medical University Jiangsu China; ^13^ Department of Anesthesiology and Perioperative Medicine, The First School of Clinical Medicine The First Affiliated Hospital of Nanjing Medical University Jiangsu China; ^14^ Changzhou Medical Center Nanjing Medical University Changzhou Jiangsu China; ^15^ Laboratory for Digital Intelligence & Health Governance Nanjing Medical University Jiangsu China

**Keywords:** DNA methylation, *MAPRE3*, non‐small cell lung cancer, overall survival, smoking cessation

## Abstract

The *MAPRE3* gene is aberrantly expressed in several cancers. We profiled DNA methylation in tumor tissues from early‐stage non‐small cell lung cancer (NSCLC) patients and assessed associations with overall survival (OS). Significant CpG probes were validated in The Cancer Genome Atlas (TCGA). The methylation level of cg12821679_*MAPRE3*_ showed significant associations with OS in lung squamous cell carcinoma (LUSC) (HR = 0.32, *P* = 6.55 × 10^−7^), but it was not observed in lung adenocarcinoma (LUAD). In LUSC, *MAPRE3* expression was significantly correlated with cg12821679_*MAPRE3*_ (*r* = 0.17, *P* = 2.96 × 10^−3^) and potential *trans*‐regulated genes were enriched in the *Nicotine addiction* pathway. Additionally, *MAPRE3* expression showed significant associations with OS in both LUAD and LUSC (LUAD: HR_low vs high_ = 2.28, *P* = 2.40 × 10^−3^; LUSC: HR_low vs high_ = 1.61, *P* = 0.0244). The association between smoking cessation and overall survival was significantly modified by *MAPRE3* expression (HR_interaction_ = 0.69, *P* = 0.0282). Smoking cessation improved OS only in patients with high *MAPRE3* expression (HR = 0.56, *P* = 2.82 × 10^−3^). We conclude *MAPRE3* may predict NSCLC prognosis and influence the prognostic benefit of smoking cessation.

## Graphical abstract

Both cg12821679_*MAPRE3*_ methylation and *MAPRE3* expression are significantly associated with overall survival (OS) of non‐small cell lung cancer. Meanwhile, *MAPRE3* expression significantly modified the effect of smoking cessation on OS. Smoking cessation benefits OS merely for patients with high *MAPRE3* expression. *MAPRE3* may serve as a prognostic biomarker and influence the prognostic benefit of smoking cessation.

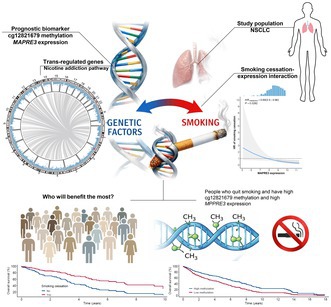

**Abbreviations**
*ALK*‐TKIs: *ALK* tyrosine kinase inhibitors
*APC*
: adenomatous polyposis coli
CI: confidence interval
CV: coefficient of variance
FDR: false discovery rate
GO: gene ontology
HR: hazard ratio
KEGG: Kyoto Encyclopedia of Genes and Genomes
LUAD: lung adenocarcinoma
LUSC: lung squamous cell carcinoma
*MAPRE3*
: microtubule‐associated protein RP/EB family member 3
MGH: Massachusetts General Hospital
NSCLC: non‐small cell lung cancer
OS: overall survival
*PTEN*
: phosphatase and tensin homolog
QC: quality control
ROS: reactive oxygen species
RSEM: RNA‐sequencing by expectation maximization
SD: standard deviation
TCGA: The Cancer Genome Atlas
UTR: untranslated region
WHO: World Health Organization

## 1 Introduction

Lung cancer was the most commonly diagnosed cancer in 2022, with an estimated nearly 2.5 million new cases, and remained the leading cause of cancer mortality worldwide, accounting for 1.82 million deaths (18.7%) [1]. Even among patients with early‐stage lung cancer undergoing surgical resection, substantial variability in outcomes persists, with mortality rates ranging from 8 to 66% [2], representing a persistent and considerable global health challenge.

Continued smoking confers a roughly 50% increased risk of all‐cause mortality and a 61% elevated risk of cancer‐specific mortality in patients with multiple cancer types. It also adversely affects the efficacy of cancer therapies across various cancer types [3]. Recent studies in patients with lung cancers indicate that quitting smoking extends life expectancy and reduces mortality rates [4, 5, 6]. Nevertheless, the specific processes driving these benefits are still not well understood [7].

DNA methylation, an inherently reversible epigenetic modification, serves as a pivotal regulator of transcriptional programs and emerges as a fertile ground for biomarker discovery and epigenetic‐based interventions in oncology [8, 9], with particular relevance to non‐small cell lung cancer (NSCLC) [10]. Notably, environmental exposures can trigger aberrant DNA methylation and transcriptomic shifts, contributing in part to the elevated rates of relapse and cancer‐specific death observed in smokers [11, 12, 13]. The multifactorial nature of cancer underscores the synergistic contributions of clinical features, environmental exposures, germline genetics, and epigenetic regulation [14, 15]. Nevertheless, most studies focus on identifying factors with marginal effects [16], overlooking epigenetic–environment interactions and thereby limiting the discovery of novel biomarkers [17]. Indeed, we have observed a series of gene‐smoking interactions which affect lung cancer prognosis in DNA methylation level [18, 19, 20, 21]. Despite this, how smoking cessation interacts with gene expression or DNA methylation to affect early‐stage NSCLC survival has not been comprehensively studied.

Microtubule‐associated protein RP/EB family member 3 (*MAPRE3*) is a component of the cytoplasmic microtubule network and belongs to the EB family of proteins. This family includes three members (*MAPRE1*, *MAPRE2*, and *MAPRE3*), which play essential roles in various cellular processes [22]. *MAPRE1* interacts with adenomatous polyposis coli (*APC*), a tumor suppressor, and plays a critical role in microtubule dynamics and cell polarity regulation [23]. Similarly, *MAPRE3* associates with an *APC* homolog (*APCL*) and contributes to microtubule depolymerization [24]. Notably, the *MAPRE3* gene is aberrantly expressed in several cancers, such as colorectal and ovarian cancers [25, 26]. While previous studies have reported prognostic associations of *MAPRE1* [27] and *MAPRE2* [28] with NSCLC, evidence regarding the impact of *MAPRE3* on NSCLC survival remains limited and inconclusive.

Thus, we utilized a two‐phase (discovery and validation) analytic design to evaluate the association between *MAPRE3* and NSCLC survival, which may also have an interaction with smoking, jointly affecting NSCLC prognosis.

## 2 Materials and methods

### 2.1 Populations of epigenomic study of lung cancer survival

Only patients with stage I–II NSCLC, specifically lung adenocarcinoma (LUAD) and lung squamous cell carcinoma (LUSC) subtypes, were included. DNA methylation data were pooled from an international multi‐cohort resource encompassing TCGA and four independent cohorts (USA‐Harvard, Spain, Norway, and Sweden) [20, 21, 29, 30]. The study methodologies conformed to the standards set by the Declaration of Helsinki.

#### 2.1.1 USA‐Harvard

Since 1992, Massachusetts General Hospital (MGH) had enrolled patients with histologically confirmed primary NSCLC [2]. We analyzed 151 early‐stage patients from this cohort. Tumor specimens were obtained during curative surgery with complete resection and immediately snap‐frozen. A MGH pathologist assessed each specimen for tumor cellularity (> 70%) and quality. Histological classification followed World Health Organization (WHO) guidelines. The study received approval from the Institutional Review Boards at Harvard T.H. Chan School of Public Health and MGH, and all patients provided written informed consent (Partners Human Research Committee, Protocol #1999P004935/MGH).

#### 2.1.2 Spain

The Spain cohort comprised 226 early‐stage NSCLC patients enrolled across eight sub‐centers from 1991 to 2009 [31]. Tumor DNA was extracted from fresh‐frozen specimens and evaluated for integrity and quantity. Patients gave written informed consent, and tumors were surgically obtained. The study was approved by the Institutional Review Boards of the Bellvitge Biomedical Research Institute (PR055/10).

#### 2.1.3 Norway

The Norway cohort included 133 early‐stage NSCLC patients enrolled at Oslo University Hospital‐Riks Hospitalet between 2006 and 2011 [32]. Tumor tissues were snap‐frozen in liquid nitrogen and stored at −80 °C prior to DNA extraction. The study was approved by the Oslo University Institutional Review Board and the Regional Ethics Committee (S‐05307), with written informed consent obtained from all patients.

#### 2.1.4 Sweden

At Skane University Hospital in Lund, Sweden, tumor DNA was obtained from 103 early‐stage NSCLC patients, comprising 80 LUAD and 23 LUSC cases [16]. The study was approved by the Regional Ethical Review Board in Lund, Sweden (registration no. 2004/762 and 2008/702), and all participants provided written informed consent.

#### 2.1.5 TCGA

The study included 332 LUAD and 285 LUSC cases with complete DNA methylation, survival time, and covariate data. On October 1, 2015, Level‐1 HumanMethylation450 DNA methylation data for early‐stage NSCLC patients were downloaded [33].

### 2.2 Quality control for DNA methylation data

DNA methylation was evaluated using Illumina Infinium HumanMethylation450 BeadChips (Illumina Inc.). Raw image data were processed in Genome Studio Methylation Module V1.8 (Illumina Inc.) to compute methylation signals, with normalization, background subtraction, and quality control (QC) performed. Probes were excluded if they met any of these criteria: (i) detection failure (*P* > 0.05) in ≥ 5% of samples, (ii) coefficient of variance (CV) < 5%, (iii) fully methylated or unmethylated across all samples, (iv) presence of common single nucleotide polymorphisms in the probe sequence or within 10‐bp flanking regions, (v) cross‐reactive probes [34], or (vi) failure to pass QC across all cohorts. Samples with > 5% undetectable probes were removed. Methylation signals underwent quantile normalization (using the *betaqn* function in R package *minfi* [35]), correction for type I and II probes (using the BMIQ function in R package *lumi* [36]), and batch effect adjustment (using the ComBat function in R package *sva* [37]). The QC process is detailed in Fig. S1.

### 2.3 Gene expression data

The TCGA workgroup processed the quality control of mRNA sequencing data. Raw counts were normalized using RNA‐sequencing by expectation maximization (RSEM). Level‐3 gene quantification data were retrieved from the TCGA data portal and subjected to additional quality check. Gene expression values were extracted and log2‐transformed prior to analysis.

### 2.4 Statistical analysis

#### 2.4.1 Association analysis between DNA methylation and lung cancer survival

**Fig. 1:**
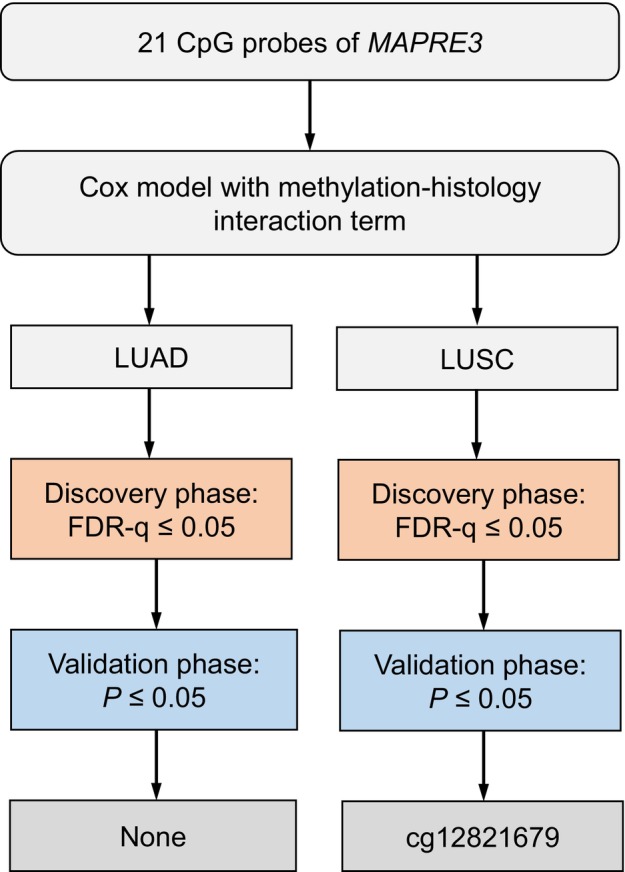
Flow chart of study design and statistical analyses. Patients from USA‐Harvard, Spain, Norway, and Sweden study cohorts were assigned to the discovery phase, while patients in TCGA were assigned to the validation phase. TCGA, The Cancer Genome Atlas; *MAPRE3*, Microtubule‐associated protein RP/EB family member 3; LUSC, lung squamous cell carcinoma; LUAD, lung adenocarcinoma; FDR, false discovery rate.

Statistical analysis flow is presented in Fig. 1. Patients from USA‐Harvard, Spain, Norway, and Sweden study cohorts were assigned to the discovery phase, while patients in TCGA were assigned to the validation phase. Multivariable Cox proportional hazards models with methylation‐histology interaction term, adjusted for age, gender, smoking status, clinical stage, histology, and study center were used to evaluate associations between each CpG and overall survival. Proportional hazards tests were conducted based on weighted residuals [38] using R package *Survival* [39]. In the discovery phase, each of the 21 CpG probes in *MAPRE3* gene was evaluated using Cox model. Multiple comparisons were corrected using Benjamini‐Hochberg method to control the false discovery rate (FDR) at 5% level. CpG probes with FDR‐*q* ≤ 0.05 in the discovery phase were further replicated in the validation phase. Robustly significant CpG probes were finally retained if they met the following criteria: (i) *P* ≤ 0.05 in the validation phase; (ii) consistent direction of coefficient across two phases. Hazard ratio (HR) and 95% confidence interval (CI) were with respect to per 10% level of methylation increment. For robustly significant CpG probes, covariate‐adjusted Kaplan–Meier curves were performed using R package *adjustedCurves* [40] to compare survival difference between patients with different methylation levels.

#### 2.4.2 Association analysis between gene expression and lung cancer survival

In TCGA cohort, all of the 281 LUAD and 277 LUSC cases had complete mRNA sequencing data. Methylation‐expression correlation analysis was performed using mRNA sequencing data from TCGA. Because the significant methylation probe cg12821679_*MAPRE3*_ was detected exclusively in LUSC patients, all subsequent analyses involving this probe were confined to the LUSC patients. While analyses of *MAPRE3* expression were performed using the combined cohort to maximize statistical power due to the significant results in both histological types of patients. A histology‐stratified Cox proportional hazards model with *MAPRE3* expression‐smoking cessation interaction term was fitted, adjusted for the same covariates aforementioned. We encoded the variable smoking cessation as ‘yes’ for former and ‘no’ for current smokers.

#### 2.4.3 Correlation analysis between DNA methylation and gene expression

Genome‐wide methylation‐expression correlation analyses were conducted using *Pearson*'s correlation coefficient to identify potential *cis*‐ and *trans*‐regulated genes by cg12821679_*MAPRE3*_ in TCGA dataset. CpG probes located within the annotated region of a gene and showing a statistically significant correlation with the expression of the gene (*P* ≤ 0.05) were defined as candidate sites for epigenetic *cis*‐regulation of transcription, whereas probes that were significantly correlated with expression of other genes were defined as candidate sites for epigenetic *trans*‐regulation of transcription. Genes were considered significant at FDR‐*q* ≤ 0.05 and were presented in a Circos plot. Gene set enrichment analyses of potential *trans*‐regulation genes were conducted using R package *clusterProfiler* based on GO and KEGG database [41].

Continuous variables were summarized as mean ± SD; categorical variables were described as *n* (%). All statistical analyses were performed in R version 4.4.2 (R Foundation for Statistical Computing, Vienna, Austria).

## 3 Results

Tumor DNA samples collected from 1230 individuals with early‐stage NSCLC, representing five separate international cohorts, underwent molecular analyses. The corresponding demographic and clinical data for the cohorts were presented in Table S1. The annotation of all 21 CpG probes located in *MAPRE3* was presented in Table S2.

### 3.1 cg12821679MAPRE3 was significantly associated with the LUSC survival

**Fig. 2:**
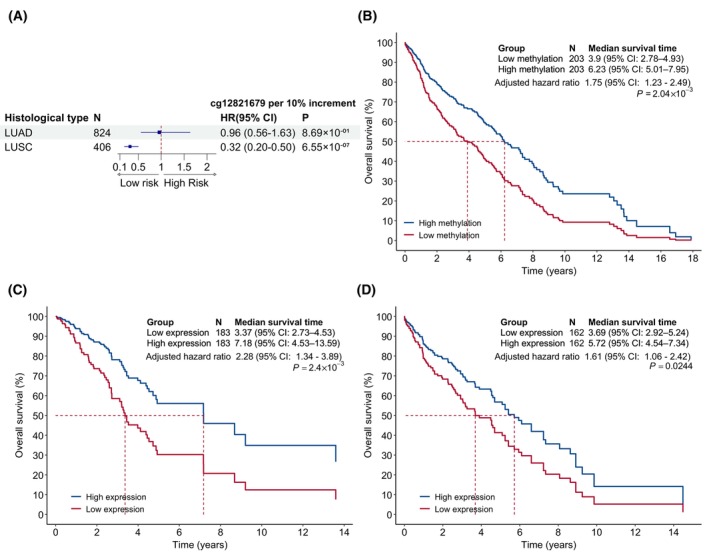
Forest plot for OS in LUAD and LUSC subgroups and Kaplan–Meier survival curves of NSCLC patients with different levels of cg12821679_*MAPRE3*_ methylation and *MAPRE3* expression. (A) Forest plot showing HRs and 95% CIs for OS in LUAD and LUSC subgroups. HR, 95% CI, and *P* values were derived from Cox proportional hazards model adjusted for age, sex, smoking status, clinical stage, and study center. (B) Kaplan–Meier survival curves of LUSC patients with high and low methylation level. Patients were categorized by median value of cg12821679_*MAPRE3*_, which is 0.94. HR, 95% CI, and *P* values were derived from Cox proportional hazards model adjusted for age, sex, smoking status, clinical stage and study center. (C) Kaplan–Meier survival curves of LUAD patients with high and low expression categorized by median value of cg12821679_*MAPRE3*_, which is 8.59 after log2 transformation. HR, 95% CI, and *P* values were derived from Cox proportional hazards model adjusted for age, sex, smoking status, and clinical stage. (D) Kaplan–Meier survival curves of LUSC patients with high and low expression categorized by median value of cg12821679_*MAPRE3*_, which is 8.89 after log2 transformation. HR, 95% CI, and *P* values were derived from Cox proportional hazards model adjusted for age, sex, smoking status and clinical stage. All dashed lines in Kaplan–Meier survival curves at a survival probability of 50% intersecting the curve represents the estimated median survival time. OS, overall survival; NSCLC, non‐small cell lung cancer; LUSC, lung squamous cell carcinoma; LUAD, lung adenocarcinoma; *MAPRE3*, Microtubule‐associated protein RP/EB family member 3; HR, hazard ratio; CI, confidence interval.

In the LUAD cohort, no CpG site reached statistical significance during the discovery phase (Table S3). However, only one probe cg12821679_*MAPRE3*_ was consistently associated with overall survival in LUSC across both the discovery and validation phases (HR_discovery_ = 0.32, 95% CI: 0.18–0.58, *P* = 1.54 × 10^−4^, FDR‐*q* = 3.18 × 10^−3^; HR_validation_ = 0.34, 95% CI: 0.17–0.70, *P* = 3.48 × 10^−3^). The association tended to be more pronounced in the combined dataset (HR_combined_ = 0.32, 95% CI: 0.21–0.50, *P* = 6.55 × 10^−7^) (Tables S4 and S5). Moreover, a meta‐analysis demonstrated a robust association (HR = 0.29, 95% CI: 0.18–0.44) that was highly consistent across sub‐cohorts, with negligible heterogeneity (*P*_heterogeneity_ = 0.81) (Fig. S2). The forest plot indicated significant heterogeneity in the association between cg12821679_*MAPRE3*_ and survival across two histological subtypes (Fig. 2A), while cg12821679_*MAPRE3*_ methylation was similarly distributed in patients with LUAD and LUSC (Fig. S3A–D). In the combined dataset, patients were classified into high‐ and low‐methylation subgroups using the median cg12821679_*MAPRE3*_ methylation level as the cutoff value. Compared with LUSC patients showing high cg12821679_*MAPRE3*_ methylation, those with low methylation levels demonstrated markedly worse survival outcomes (HR_low vs high_ = 1.75, 95% CI: 1.23–2.49, *P* = 2.04 × 10^−3^) (Fig. 2B and Table S6). The Kaplan–Meier plot confirmed the significant heterogeneity in the association of cg12821679_*MAPRE3*_ with survival between the two groups.

### 3.2 *MAPRE3* expression was significantly associated with the NSCLC survival

Patients were classified into low‐ and high‐expression subgroups using the median *MAPRE3* expression as the cutoff value. And these two groups exhibited significant differences in overall survival, indicating a prognostic relevance of *MAPRE3* expression (LUAD: HR_low vs high_ = 2.28, 95% CI: 1.34–3.89, *P* = 2.40 × 10^−3^; LUSC: HR_low vs high_ = 1.61, 95% CI: 1.06–2.42, *P* = 0.0244) (Fig. 2C,D and Table S7).

### 3.3 Gene expression was potentially *cis*‐ and *trans*‐regulated by cg12821679_*MAPRE3*_

**Fig. 3:**
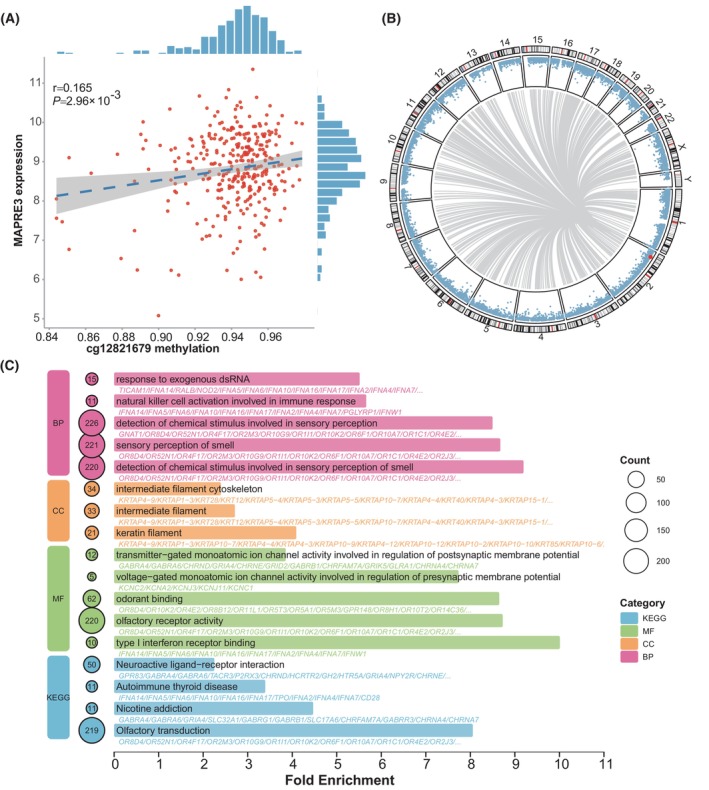
Scatter plot of *cis*‐regulation, Circos plot of genome‐wide *trans*‐regulation analysis, and significant pathways of gene enrichment pathway analysis. (A) Scatter plot of correlation between DNA methylation of cg12821679_*MAPRE3*_ and expression of *MAPRE3* in TCGA cohort. Correlation coefficient and hypothesis test were derived from *Pearson* correlation analysis. Gene expression was log_2_‐transformed before analysis. Histogram on top shows the distribution of cg12821679_*MAPRE3*_ methylation; histogram on side shows the distribution of *MAPRE3* expression. The dashed line represents the fitted linear regression line and the gray band indicates the corresponding confidence interval. (B) Circos plot of genome‐wide *trans*‐regulation analysis in the TCGA cohort. Red point represents *P* value of correlation between *MAPRE3* expression and cg12821679_*MAPRE3*_ methylation, while blue points ordered by genomic position represent *P* values of correlation between expression of other genes and methylation level of cg12821679_*MAPRE3*_. Gray lines represent significant correlations with FDR‐*q* ≤ 0.05. (C) Gene set enrichment analysis of 1299 genes correlated with cg12821679_*MAPRE3*_ methylation based on KEGG and GO database. *MAPRE3*, Microtubule‐associated protein RP/EB family member 3; TCGA, The Cancer Genome Atlas; FDR, false discovery rate; KEGG, Kyoto Encyclopedia of Genes and Genomes; GO, Gene Ontology; MF, Molecular Function; CC, Cellular Component; BP, Biological Process.

**Fig. 4:**
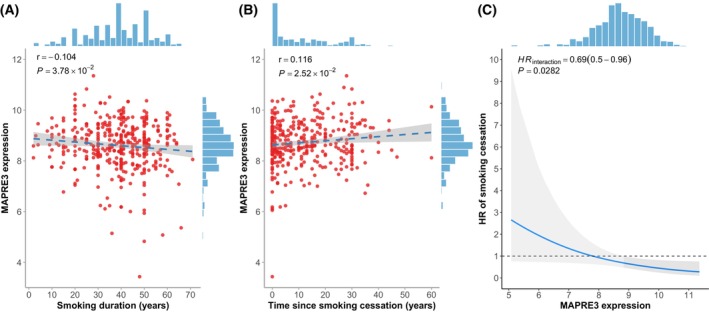
Scatter plot of *MAPRE3* expression–smoking correlation and curve of *MAPRE3* expression–smoking cessation interaction. (A) Correlation between smoking duration (years) and *MAPRE3* expression. (B) Correlation between time since smoking cessation (years) and *MAPRE3* expression. Correlation coefficient and hypothesis test were derived from *Pearson* correlation analysis. Gene expression was log2‐transformed before analysis. The dashed line represents the fitted linear regression line and the gray band indicates the corresponding confidence interval. (C) *MAPRE3* expression–smoking cessation interaction effect on survival of NSCLC patients. The blue curve shows the estimated HR for smoking cessation across *MAPRE3* expression levels and the gray shaded area indicates the 95% CI. The horizontal dashed line marks the null value of HR (1.0). HR, 95% CI, and *P* value of interaction were derived from histology‐stratified Cox proportional hazards model adjusted for age, sex, smoking cessation, and clinical stage. Shallow area represented 95% CI. Histogram on the top shows the distribution of *MAPRE3* expression. All analyses in this figure were conducted in TCGA cohort. *MAPRE3*, Microtubule‐associated protein RP/EB family member 3; HR, hazard ratio; CI, confidence interval; NSCLC, non‐small cell lung cancer; TCGA, The Cancer Genome Atlas.

*MAPRE3* expression levels showed a significant correlation with cg12821679_*MAPRE3*_ methylation in LUSC patients (*r* = 0.17, *P* = 2.96 × 10^−3^) (Fig. 3A), indicating that methylation of cg12821679_*MAPRE3*_ may *cis*‐regulate *MAPRE3* expression. Further, through genome‐wide *trans*‐regulation analysis, we identified 1299 genes whose expression was significantly correlated with cg12821679_*MAPRE3*_ methylation (Table S8; Fig. 3B). Gene set enrichment analyses performed on the 1299 potential *trans*‐regulated genes identified significant enrichment in multiple immune‐related pathways, including *Response to exogenous dsRNA*, *Natural killer cell activation involved in immune response*, and *Autoimmune thyroid disease*. Notably, the enrichment analyses also revealed significant enrichment of the *trans*‐regulated genes in the *nicotine addiction* pathway (Fig. 3C). Additionally, *MAPRE3* expression was also significantly correlated with smoking duration (years) and time since smoking cessation (years) (Fig. 4A,B).

### 3.4 High *MAPRE3* expression enhanced the benefit of smoking cessation to NSCLC survival

No significant interaction was observed between cg12821679_*MAPRE3*_ and smoking cessation (Table S9), whereas *MAPRE3* expression showed a significant interaction with smoking cessation in NSCLC patients (HR_interaction_ = 0.69, 95% CI: 0.502–0.962, *P* = 0.0282) (Table S10). Specifically, elevated *MAPRE3* expression significantly strengthened the favorable association between smoking cessation and overall survival in NSCLC (Fig. 4C), suggesting a moderating role of *MAPRE3* expression in this relationship.

**Fig. 5:**
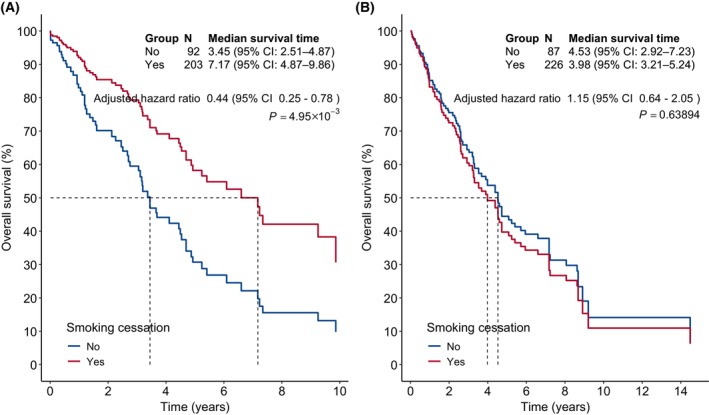
Kaplan–Meier survival curves of NSCLC patients categorized into current smokers (without smoking cessation) and former smokers (who had quit smoking). Patients with high and low expression in the NSCLC patients categorized by median value of *MAPRE3* expression, which is 8.71 after log2 transformation. (A) Kaplan–Meier survival curves of NSCLC patients with high *MAPRE3* expression. (B) Kaplan–Meier survival curves of NSCLC patients with low *MAPRE3* expression. HR, 95% CI, and *P* values were derived from the histology‐stratified Cox proportional hazards model adjusted for age, sex, and clinical stage. All dashed lines in Kaplan–Meier survival curves at a survival probability of 50% intersect the curve represents the estimated median survival time. *MAPRE3*, Microtubule‐associated protein RP/EB family member 3; HR, hazard ratio; CI, confidence interval; NSCLC, non‐small cell lung cancer.

To illustrate potential modification by *MAPRE3* expression, we evaluated the association between smoking cessation and NSCLC survival among patients with low versus high *MAPRE3* expression. Stratification by *MAPRE3* expression levels revealed heterogeneity in the survival benefit of smoking cessation, which was observed only among NSCLC patients with high *MAPRE3* expression (HR_high_ = 0.46, 95% CI: 0.27–0.80, *P* = 6.33 × 10^−3^) (Fig. 5A and Table S11). No statistically significant association was found between smoking cessation and overall survival among NSCLC patients with low *MAPRE3* expression (HR_low_ = 1.13, 95% CI: 0.63–2.03, *P* = 0.6725) (Fig. 5B). Collectively, these results suggest that the prognostic advantage associated with smoking cessation in NSCLC was most pronounced in patients with high levels of *MAPRE3* expression. Among NSCLC patients with high *MAPRE3* expression, smoking cessation was associated with an exceptionally pronounced survival benefit, suggesting that *MAPRE3* expression may serve as a valuable biomarker for identifying individuals who derive extraordinary benefit from quitting smoking.

## 4 Discussion

We performed both DNA methylation and transcriptomic analyses of *MAPRE3* in early‐stage NSCLC. Notably, the CpG site cg12821679_*MAPRE3*_, located within the 3'untranslated region (UTR) of *MAPRE3*, emerged as a prognostic biomarker exclusively for early‐stage LUSC. In addition, cg12821679_*MAPRE3*_ was found to potentially *cis*‐regulate *MAPRE3* expression, which was further interacted with smoking cessation to influence NSCLC survival.

Previous studies have consistently demonstrated a higher prevalence of smoking history among patients with LUSC compared to those with LUAD [20, 42, 43]. Moreover, cigarette smoking confers a markedly greater risk for the development of LUSC than for LUAD [42]. Persistent smoking or post‐diagnosis relapse remains prevalent among lung cancer patients, with estimates suggesting that nearly half may continue tobacco use despite awareness of its harmful effects [44, 45]. In our study, the potential *trans*‐regulated genes associated with cg12821679_*MAPRE3*_ methylation in LUSC patients were significantly enriched in the *nicotine addiction* pathway. Also, patients in low cg12821679_*MAPRE3*_ methylation group showed worse survival than those in high methylation group. Our findings suggest that smoking cessation may be particularly critical for LUSC patients with low methylation level of cg12821679_*MAPRE3*_.

Numerous studies have consistently demonstrated that cigarette smoking is associated with extensive alterations in DNA methylation patterns in peripheral blood. However, a recent study has revealed a subset of CpGs exhibiting reverse or bidirectional causality with smoking behavior [46], indicating that methylation variation at these sites may modestly influence smoking susceptibility. The insights challenge the traditional view of methylation as a mere exposure biomarker, suggesting that certain CpGs could serve dual roles as indicators of tobacco use and partial contributors to smoking liability. The cg12821679_*MAPRE3*_ methylation may offer a novel biological perspective for understanding the underlying mechanisms. Future efforts must incorporate longitudinal cohorts and experimental validations to probe causality at candidate *loci*.

Moreover, among NSCLC patients with high *MAPRE3* expression, those who had quit smoking exhibited superior survival outcomes compared to current smokers, who remained under continuous and cumulative tobacco exposure. However, in the subgroup with low expression, no significant difference in prognosis was observed between current smokers and former smokers. Additionally, smoking duration and time since smoking cessation were both significantly correlated with the expression of *cis*‐regulated gene *MAPRE3* in the NSCLC population. Collectively, these findings suggest that *MAPRE3* expression modifies the survival benefit associated with smoking cessation and may be therapeutically actionable—supported by external evidence that *MAPRE3* can be de‐repressed via epigenetic therapy and that *MAPRE3* itself is druggable with small‐molecule inhibitors *in vivo* [26, 47, 48].

Although many studies indicated that smoking cessation conferred a survival benefit in lung cancer patients [6, 49, 50], other studies reported negative results [51, 52]. These inconsistencies highlight an unresolved question, thereby underscoring the need to identify modifying factors that may account for this heterogeneity. On the basis of our interaction analyses, we hypothesize that gene expression may serve as an effect modifier underlying the observed inconsistencies. Specifically, the impact of smoking cessation appears to differ according to gene expression, such that in a heterogeneous population with mixed gene expression levels, the beneficial effect may be attenuated or obscured. Consequently, conventional marginal analyses are underpowered to detect statistically significant associations in the presence of such complex effect heterogeneity.

*MAPRE3* exhibits aberrant expression across multiple malignancies, including ovarian cancer [26], colorectal adenocarcinoma [53] and prostate cancer [22]. In addition, *MAPRE3* may undergo fusion with the *ALK* in NSCLC patients, resulting in alterations that impact the therapeutic efficacy of *ALK* tyrosine kinase inhibitors (ALK‐TKIs) [48]. Notably, apart from binding *APCL*, *MAPRE3* also interacts with APC, which may inhibit the *Wnt/β‐catenin* signaling pathway [54]. The pathway drives tumorigenesis through stabilization of β‐catenin, nuclear translocation, and consequent activation of oncogenic target genes [55]. Thus, the *MAPRE3*–*APC* interaction may be critical for tumorigenesis and metastasis, which is similar to *MAPRE1* [56].

As a key exogenous source of reactive oxygen species (ROS), cigarette smoke has been demonstrated to suppress phosphatase and tensin homolog (*PTEN*) expression by promoting phosphorylation events within the Src/EGFR‐p38MAPK signaling axis [57]. Functionally, *PTEN* exerts its tumor‐suppressive activity predominantly via negative regulation of the *PI3K* signaling pathway [58]. Prolonged exposure to cigarette smoke in individuals with extensive smoking histories may therefore drive sustained ROS‐dependent repression of *PTEN*, establishing a mechanistic link between tobacco‐induced oxidative stress and impaired tumor suppressor function, which likely underpins the unfavorable prognosis observed in NSCLC.

**Fig. 6:**
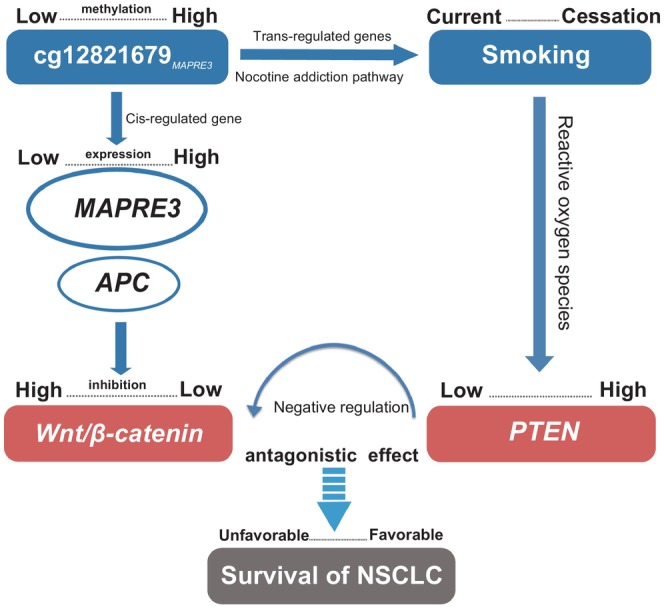
Diagram for pathway of expression–smoking cessation interaction for LUSC patients. The 1299 trans‐regulated genes in the diagram were identified in genome‐wide methylation‐expression correlation analyses using *Pearson*'s correlation coefficient in TCGA dataset, which were then found to be enriched in the *Nicotine addiction* pathway based on KEGG and GO database. LUSC, lung squamous cell carcinoma; *MAPRE3*, Microtubule‐associated protein RP/EB family member 3; *APC*, adenomatous polyposis coli; *PTEN*, phosphatase and tensin homolog; NSCLC, non‐small cell lung cancer; TCGA, The Cancer Genome Atlas; GO, Gene Ontology; KEGG, Kyoto Encyclopedia of Genes and Genomes.

Moreover, *PTEN* can inhibit *Wnt/β‐catenin* pathways [59]. Although *PTEN* acts as an upstream regulator of the *Wnt/β‐catenin* pathway, this pathway can in turn suppress *PTEN* expression through direct transcriptional activation of miR‐21 [60, 61]. Regarding the interaction between *MAPRE3* expression and smoking cessation, elevated expression was associated with a more favorable prognosis in former smokers compared to current smokers, probably because of the regulation between *Wnt/β‐catenin* and *PTEN* (Fig. 6). Nevertheless, additional mechanistic studies are required to fully delineate how *MAPRE3* interacts with smoking cessation to influence survival outcomes in NSCLC patients.

Our study has several strengths. First, we found the new methylation site that may modestly influence smoking susceptibility and NSCLC prognosis. Second, by effectively controlling for false positives, the two‐stage and multicenter study design enhanced the robustness and reliability of our findings. Third, to the best of our knowledge, this represents the first study to explore the interaction between *MAPRE3* and smoking cessation, providing insights into the heterogeneous impact of smoking cessation modulated by *MAPRE3* expression levels. Fourth, beyond the statistically significant interaction, we elaborated on a plausible functional interaction between two pathways, drawing on established literature evidence. As a future direction, integrating multiple smoking‐interaction–related genes and/or CpG sites into a composite multi‐marker score may improve risk stratification and predictive performance, pending rigorous model development and external validation.

This study has several limitations that should be considered when interpreting the results. First, although we identified a correlation between *MAPRE3* expression and smoking duration, as well as time since smoking cessation, these correlations were relatively weak, and the underlying biological mechanisms remain unclear. Similarly, although genes correlated with cg12821679_*MAPRE3*_ were enriched in the *Nicotine Addiction* pathway, the precise biological mechanisms driving this association remain undefined. Therefore, these findings should be interpreted with caution, and further investigation is needed to elucidate the biological underpinnings. Second, the relatively high censoring rate in the TCGA dataset may reduce statistical power to some extent; nevertheless, the prognostic association with cg12821679_*MAPRE3*_ retained statistical significance, suggesting that our estimates are likely robust. Third, the observed positive correlation between cg12821679_*MAPRE3*_ methylation and *MAPRE3* expression has not yet been documented in prior literature, underscoring the need for additional mechanistic investigations to validate this relationship. Fourth, our findings regarding the *MAPRE3*–smoking interaction emerged solely from exploratory analyses in TCGA dataset, and thus warrant confirmation in larger, well‐powered cohorts to substantiate their validity. Fifth, given the overrepresentation of European ancestry in our study (89.19%), these results should be interpreted with caution regarding their transferability to populations with distinct genetic ancestries.

## 5 Conclusion

In summary, this study highlights *MAPRE3* as a promising prognostic indicator in NSCLC and proposes that its interaction with smoking cessation may partially explain the heterogeneous survival benefits associated with smoking cessation. These findings suggest that *MAPRE3* could represent a modifiable therapeutic target with potential clinical relevance for patients with NSCLC.

## Conflict of interest

The authors declare no conflicts of interest.

## Author contributions

CC, JC, RH, XZ, DCC, FC, XC, and RZ contributed to conception and design. DCC, RZ, and FC contributed to financial support. LS, MMB, AK, MP, JS, ÅH, ME, DCC, and RZ contributed to data collection and quality control. CC, DCC, XC, and RZ contributed to data analysis and interpretation. CC and RZ contributed to manuscript writing. CC, DCC, and RZ contributed to critical revision. All authors contributed to final approval of the manuscript and are accountable for all aspects of the work.

## Consent

All participants or their surrogate care providers gave written informed consent. All authors have reviewed the manuscript and consented to its publication.

## Supporting information


**Table S1.** Demographic and clinical characteristics for early‐stage non‐small cell lung cancer (NSCLC) patients from five cohorts.
**Table S2.** Annotation information for 21 CpG probes located in *MAPRE3*.
**Table S3.** Results of association analysis of 21 DNA methylation probes of *MAPRE3* in lung adenocarcinoma (LUAD) patients.
**Table S4.** Results of association analysis of 21 DNA methylation probes of *MAPRE3* in lung squamous cell carcinoma (LUSC) patients.
**Table S5.** Results of proportional hazards test for cg12821679 in LUSC samples. The CpG probe was treated as a continuous variable in the model.
**Table S6.** Results of proportional hazards test for cg12821679 in LUSC samples. Patients were stratified into high‐ and low‐methylation groups based on the median methylation level of the cg12821679_*MAPRE3*_ and the CpG probe was treated as a binary variable in the model.
**Table S7.** Results of proportional hazards test for *MAPRE3* expression in LUAD and LUSC samples. Patients were stratified into high and low expression groups based on the median *MAPRE3* expression and the *MAPRE3* expression was treated as a binary variable in the model.
**Table S8.** Results of trans‐regulation analysis of the significant 1299 genes associated with cg12821679_*MAPRE3*_ in TCGA NSCLC samples.
**Table S9.** Results of cg12821679_*MAPRE3*_‐smoking cessation interaction analysis in LUAD and LUSC patients.
**Table S10.** Results of *MAPRE3* expression‐smoking cessation interaction analysis in NSCLC patients.
**Table S11.** Results of proportional hazards test for *MAPRE3* expression in NSCLC samples.
**Fig. S1.** Quality control procedures for epigenome‐wide DNA methylation data.
**Fig. S2.** Meta‐analysis of association between DNA methylation and LUSC prognosis from four cohorts (HSPH, Spain, Sweden and TCGA).
**Fig. S3.** Distribution of cg12821679_*MAPRE3*_ in LUAD and LUSC patients.

## Data Availability

The DNA methylation image data from the USA Harvard cohort can be requested from DCC. Data from the Spain, Norway, and Sweden cohorts can be requested from ME, A° H, and JS, respectively. TCGA: https://tcga‐data.nci.nih.gov; now hosted at GDC: https://portal.gdc.cancer.gov.
