# Supplementary material for: DNA methylation and expression of MAPRE3 affect overall survival of early‐stage non‐small cell lung cancer patients
Source: Mol Oncol. 2026 Apr 25:10.1002/1878-0261.70260. Online ahead of print. doi: 10.1002/1878-0261.70260 (PMC13398577; doi:10.1002/1878-0261.70260)
Supplement: Supplementary file 1 — Table S1. Demographic and clinical characteristics for early‐stage non‐small cell lung cancer (NSCLC) patients from five cohorts. Table S2. Annotation information for 21 CpG probes located in MAPRE3. Table S3. Results of association analysis of 21 DNA methylation probes of MAPRE3 in lung adenocarcinoma (LUAD) patients. Table S4. Results of association analysis of 21 DNA methylation probes of MAPRE3 in lung squamous cell carcinoma (LUSC) patients. Table S5. Results of proportional hazards test for cg12821679 in LUSC samples. The CpG probe was treated as a continuous variable in the model. Table S6. Results of proportional hazards test for cg12821679 in LUSC samples. Patients were stratified into high‐ and low‐methylation groups based on the median methylation level of the cg12821679 MAPRE3 and the CpG probe was treated as a binary variable in the model. Table S7. Results of proportional hazards test for MAPRE3 expression in LUAD and LUSC samples. Patients were stratified into high and low expression groups based on the median MAPRE3 expression and the MAPRE3 expression was treated as a binary variable in the model. Table S8. Results of trans‐regulation analysis of the significant 1299 genes associated with cg12821679 MAPRE3 in TCGA NSCLC samples. Table S9. Results of cg12821679 MAPRE3 ‐smoking cessation interaction analysis in LUAD and LUSC patients. Table S10. Results of MAPRE3 expression‐smoking cessation interaction analysis in NSCLC patients. Table S11. Results of proportional hazards test for MAPRE3 expression in NSCLC samples. Fig. S1. Quality control procedures for epigenome‐wide DNA methylation data. Fig. S2. Meta‐analysis of association between DNA methylation and LUSC prognosis from four cohorts (HSPH, Spain, Sweden and TCGA). Fig. S3. Distribution of cg12821679 MAPRE3 in LUAD and LUSC patients. [file MOL2-9999-0-s001.docx]

**Table S1.** Demographic and clinical characteristics for early-stage non-small cell lung cancer (NSCLC) patients from five cohorts.

| Variable | Discovery phase | | | | | Validation phase | Combined dataset |
| --- | --- | --- | --- | --- | --- | --- | --- |
|  | Cohort 1: Harvard  (*N* = 151) | Cohort 2: Spain^a^  (*N* = 226) | Cohort 3: Norway  (*N* = 133) | Cohort 4: Sweden  (*N* = 103) | Discovery: All  (*N* = 613) | Cohort 5: TCGA  (*N* = 617) | Overall samples  (*N* = 1230) |
| Age (years) | 67.67±9.92 | 65.67±10.58 | 65.52±9.34 | 67.54±9.99 | 66.44±10.08 | 66.51±9.47 | 66.48±9.78 |
| Sex, *n* (%) |  |  |  |  |  |  |  |
| Female | 67 (44.37) | 105 (46.46) | 71 (53.38) | 54 (52.43) | 297 (48.45) | 255 (41.33) | 552 (44.88) |
| Male | 84 (55.63) | 121 (53.54) | 62 (46.62) | 49 (47.57) | 316 (51.55) | 362 (58.67) | 678 (55.12) |
| Smoking status, *n* (%) |  |  |  |  |  |  |  |
| Never | 18 (11.92) | 30 (13.57) | 17 (12.78) | 18 (17.48) | 83 (13.65) | 55 (9.18) | 138(11.22) |
| Former | 81 (53.64) | 120 (54.30) | 74 (55.64) | 54 (52.43) | 329 (54.11) | 376 (62.77) | 705 (58.41) |
| Current | 52 (34.44) | 71 (32.13) | 42 (31.58) | 31 (30.10) | 196 (32.24) | 168 (28.05) | 364 (30.16) |
| Unknown | 0 | 5 | 0 | 0 | 5 | 18 | 23 |
| Clinical stage, *n* (%) |  |  |  |  |  |  |  |
| I | 104 (68.87) | 183 (80.97) | 93 (69.92) | 95 (92.23) | 475 (77.49) | 393 (63.70) | 868 (70.57) |
| II | 47 (31.13) | 43 (19.03) | 40 (30.08) | 8 (7.77) | 138 (22.51) | 224 (36.30) | 362 (29.43) |
| Histology, *n* (%) |  |  |  |  |  |  |  |
| LUAD | 96 (63.58) | 183 (80.97) | 133 (100.00) | 80 (77.67) | 492 (80.26) | 332 (53.81) | 824 (66.99) |
| LUSC | 55 (36.42) | 43 (19.03) | 0 (0.00) | 23 (22.33) | 121 (19.74) | 285 (46.19) | 406 (33.01) |
| Chemotherapy, *n* (%) |  |  |  |  |  |  |  |
| No | 142 (94.04) | 177 (90.77) | 102 (76.69) | 67 (90.54) | 488 (88.25) | 194 (76.98) | 682 (84.72) |
| Yes | 9 (5.96) | 18 (9.23) | 31 (23.31) | 7 (9.46) | 65 (11.75) | 58 (23.02) | 123 (15.28) |
| Unknown | 0 | 31 | 0 | 29 | 60 | 365 | 425 |
| Radiotherapy, *n* (%) |  |  |  |  |  |  |  |
| No | 132 (87.42) | 184 (94.36) | 132 (99.25) | 74 (100.00) | 522 (94.39) | 239 (94.84) | 761 (94.53) |
| Yes | 19 (12.58) | 11 (5.64) | 1 (0.75) | 0 (0.00) | 31 (5.61) | 13 (5.16) | 44 (5.47) |
| Unknown | 0 | 31 | 0 | 29 | 60 | 365 | 425 |
| Adjuvant therapy^b^, *n* (%) |  |  |  |  |  |  |  |
| No | 127 (84.11) | 168 (86.15) | 101 (75.94) | 67 (90.54) | 463 (83.73) | 187 (74.21) | 650 (80.75) |
| Yes | 24 (15.89) | 27 (13.85) | 32 (24.06) | 7 (9.46) | 90 (16.27) | 65 (25.79) | 155 (19.25) |
| Unknown | 0 | 31 | 0 | 29 | 60 | 365 | 425 |
| Survival year |  |  |  |  |  |  |  |
| Median (95% CI) | 6.66 (5.41-7.87) | 7.12 (5.06-9.63) | 7.36 (6.77-7.95)^*^ | 7.39 (4.98-9.12) | 7.39 (6.50-8.23) | 4.54 (3.68-5.41) | 6.60 (5.84-7.35) |
| Dead (%) | 122 (80.79) | 101 (44.69) | 42 (31.58) | 58 (31.58) | 323 (52.69) | 142 (23.01) | 465 (37.80) |

^a^Cohort 2: Spain is a collaborative cohort, recruiting samples from Spain, Italy, UK, France, and USA.

^b^Adjuvant therapy includes chemotherapy or radiotherapy. ^*^Restricted mean survival time is given since median survival time is not available.

**Table S2.** Annotation information for 21 CpG probes located in *MAPRE3*.

| **Probe** | **Location** | **Region** | **CpG island** | **Relation to CpG islands** |
| --- | --- | --- | --- | --- |
| cg01925962 | Chr2:27193339 | TSS200 | chr2:27193273-27193864 | Island |
| cg03754074 | Chr2:27248790 | Body |  |  |
| cg04059696 | Chr2:27211953 | 5'UTR |  |  |
| cg04400587 | Chr2:27193588 | 1stExon;5'UTR | chr2:27193273-27193864 | Island |
| cg06350345 | Chr2:27239739 | 5'UTR |  |  |
| cg07495389 | Chr2:27233872 | 5'UTR |  |  |
| cg08791692 | Chr2:27193447 | TSS200 | chr2:27193273-27193864 | Island |
| cg08940001 | Chr2:27193211 | TSS1500 | chr2:27193273-27193864 | N_Shore |
| cg09337677 | Chr2:27193299 | TSS1500 | chr2:27193273-27193864 | Island |
| cg09708084 | Chr2:27247208 | Body |  |  |
| cg11153596 | Chr2:27210232 | 5'UTR |  |  |
| **cg12821679** | **Chr2:27249349** | **3'UTR** |  |  |
| cg15315096 | Chr2:27193331 | TSS200 | chr2:27193273-27193864 | Island |
| cg16212114 | Chr2:27193496 | TSS200 | chr2:27193273-27193864 | Island |
| cg17192247 | Chr2:27194436 | 5'UTR | chr2:27193273-27193864 | S_Shore |
| cg18625303 | Chr2:27226681 | 5'UTR |  |  |
| cg24321774 | Chr2:27193835 | 5'UTR | chr2:27193273-27193864 | Island |
| cg25029561 | Chr2:27194315 | 5'UTR | chr2:27193273-27193864 | S_Shore |
| cg25377468 | Chr2:27200226 | 5'UTR |  |  |
| cg25515091 | Chr2:27196810 | 5'UTR | chr2:27193273-27193864 | S_Shelf |
| cg25781926 | Chr2:27239543 | 5'UTR |  |  |

**Table S3.** Results of association analysis of 21 DNA methylation probes of *MAPRE3* in lung adenocarcinoma (LUAD) patients.

| CpG probe | Discovery phase | | | | | Validation phase | | | | Combined data | | | |
| --- | --- | --- | --- | --- | --- | --- | --- | --- | --- | --- | --- | --- | --- |
|  | HR | 95% CI | | *P* | FDR-q | HR | 95% CI | | *P* | HR | 95% CI | | *P* |
| cg07495389 | 0.899 | 0.81 | 0.997 | 4.316 × 10^-^⁰² | 3.021 × 10^-^⁰¹ | 0.943 | 0.774 | 1.149 | 5.614 × 10^-^⁰¹ | 0.898 | 0.82 | 0.984 | 2.131 × 10^-^⁰² |
| cg25377468 | 0.6 | 0.274 | 1.313 | 2.007 × 10^-^⁰¹ | 7.269 × 10^-^⁰¹ | 0.118 | 0.04 | 0.353 | 1.300 × 10^-^⁰⁴ | 0.434 | 0.213 | 0.887 | 2.212 × 10^-^⁰² |
| cg17192247 | 1.056 | 0.937 | 1.19 | 3.750 × 10^-^⁰¹ | 7.269 × 10^-^⁰¹ | 1.261 | 1.058 | 1.504 | 9.767 × 10^-^⁰³ | 1.1 | 0.995 | 1.216 | 6.393 × 10^-^⁰² |
| cg01925962 | 3.38 | 0.142 | 80.71 | 4.519 × 10^-^⁰¹ | 7.269 × 10^-^⁰¹ | 245.683 | 0.961 | 62837.331 | 5.169 × 10^-^⁰² | 6.559 | 0.407 | 105.775 | 1.849 × 10^-^⁰¹ |
| cg25515091 | 1.236 | 0.966 | 1.58 | 9.178 × 10^-^⁰² | 4.818 × 10^-^⁰¹ | 0.888 | 0.655 | 1.203 | 4.432 × 10^-^⁰¹ | 1.133 | 0.922 | 1.392 | 2.355 × 10^-^⁰¹ |
| cg18625303 | 0.897 | 0.722 | 1.115 | 3.284 × 10^-^⁰¹ | 7.269 × 10^-^⁰¹ | 0.934 | 0.67 | 1.301 | 6.848 × 10^-^⁰¹ | 0.902 | 0.749 | 1.085 | 2.736 × 10^-^⁰¹ |
| cg25781926 | 0.92 | 0.854 | 0.991 | 2.797 × 10^-^⁰² | 3.021 × 10^-^⁰¹ | 1.341 | 1.028 | 1.749 | 3.042 × 10^-^⁰² | 0.961 | 0.892 | 1.036 | 3.011 × 10^-^⁰¹ |
| cg08791692 | 1.207 | 0.248 | 5.868 | 8.154 × 10^-^⁰¹ | 9.514 × 10^-^⁰¹ | 62.383 | 1.846 | 2108.037 | 2.137 × 10^-^⁰² | 1.939 | 0.462 | 8.144 | 3.659 × 10^-^⁰¹ |
| cg04400587 | 0.168 | 0.032 | 0.868 | 3.333 × 10^-^⁰² | 3.021 × 10^-^⁰¹ | 6.65 | 0.064 | 692.837 | 4.241 × 10^-^⁰¹ | 0.203 | 0.045 | 0.921 | 3.873 × 10^-^⁰² |
| cg15315096 | 0.565 | 0.134 | 2.376 | 4.358 × 10^-^⁰¹ | 7.269 × 10^-^⁰¹ | 12.085 | 1.179 | 123.906 | 3.587 × 10^-^⁰² | 1.344 | 0.42 | 4.297 | 6.185 × 10^-^⁰¹ |
| cg09337677 | 0.964 | 0.144 | 6.461 | 9.701 × 10^-^⁰¹ | 9.701 × 10^-^⁰¹ | 0.001 | 0 | 50.723 | 2.205 × 10^-^⁰¹ | 0.674 | 0.092 | 4.966 | 6.990 × 10^-^⁰¹ |
| cg24321774 | 0.981 | 0.702 | 1.37 | 9.100 × 10^-^⁰¹ | 9.555 × 10^-^⁰¹ | 0.508 | 0.176 | 1.464 | 2.096 × 10^-^⁰¹ | 0.952 | 0.695 | 1.304 | 7.592 × 10^-^⁰¹ |
| cg03754074 | 1.285 | 0.762 | 2.165 | 3.471 × 10^-^⁰¹ | 7.269 × 10^-^⁰¹ | 0.423 | 0.164 | 1.093 | 7.558 × 10^-^⁰² | 1.055 | 0.665 | 1.675 | 8.203 × 10^-^⁰¹ |
| cg07495389 | 0.899 | 0.81 | 0.997 | 4.316 × 10^-^⁰² | 3.021 × 10^-^⁰¹ | 0.943 | 0.774 | 1.149 | 5.614 × 10^-^⁰¹ | 0.898 | 0.82 | 0.984 | 2.131 × 10^-^⁰² |
| cg25377468 | 0.6 | 0.274 | 1.313 | 2.007 × 10^-^⁰¹ | 7.269 × 10^-^⁰¹ | 0.118 | 0.04 | 0.353 | 1.300 × 10^-^⁰⁴ | 0.434 | 0.213 | 0.887 | 2.212 × 10^-^⁰² |
| cg17192247 | 1.056 | 0.937 | 1.19 | 3.750 × 10^-^⁰¹ | 7.269 × 10^-^⁰¹ | 1.261 | 1.058 | 1.504 | 9.767 × 10^-^⁰³ | 1.1 | 0.995 | 1.216 | 6.393 × 10^-^⁰² |
| cg01925962 | 3.38 | 0.142 | 80.71 | 4.519 × 10^-^⁰¹ | 7.269 × 10^-^⁰¹ | 245.683 | 0.961 | 62837.331 | 5.169 × 10^-^⁰² | 6.559 | 0.407 | 105.775 | 1.849 × 10^-^⁰¹ |
| cg25515091 | 1.236 | 0.966 | 1.58 | 9.178 × 10^-^⁰² | 4.818 × 10^-^⁰¹ | 0.888 | 0.655 | 1.203 | 4.432 × 10^-^⁰¹ | 1.133 | 0.922 | 1.392 | 2.355 × 10^-^⁰¹ |
| cg18625303 | 0.897 | 0.722 | 1.115 | 3.284 × 10^-^⁰¹ | 7.269 × 10^-^⁰¹ | 0.934 | 0.67 | 1.301 | 6.848 × 10^-^⁰¹ | 0.902 | 0.749 | 1.085 | 2.736 × 10^-^⁰¹ |
| cg25781926 | 0.92 | 0.854 | 0.991 | 2.797 × 10^-^⁰² | 3.021 × 10^-^⁰¹ | 1.341 | 1.028 | 1.749 | 3.042 × 10^-^⁰² | 0.961 | 0.892 | 1.036 | 3.011 × 10^-^⁰¹ |
| cg08791692 | 1.207 | 0.248 | 5.868 | 8.154 × 10^-^⁰¹ | 9.514 × 10^-^⁰¹ | 62.383 | 1.846 | 2108.037 | 2.137 × 10^-^⁰² | 1.939 | 0.462 | 8.144 | 3.659 × 10^-^⁰¹ |

HR: hazard ratio; 95% CI: 95% confidence interval.

**Table S4.** Results of association analysis of 21 DNA methylation probes of *MAPRE3* in lung squamous cell carcinoma (LUSC) patients.

| CpG probe | Discovery phase | | | | | Validation phase | | | | Combined data | | | |
| --- | --- | --- | --- | --- | --- | --- | --- | --- | --- | --- | --- | --- | --- |
|  | HR | 95% CI | | *P* | FDR-q | HR | 95% CI | | *P* | HR | 95% CI | | *P* |
| **cg12821679** | **0.323** | **0.18** | **0.579** | **1.513 × 10^-^⁰⁴** | **3.177 × 10^-^⁰³** | **0.34** | **0.165** | **0.701** | **3.482 × 10^-^⁰³** | **0.321** | **0.205** | **0.502** | **6.553 × 10^-^⁰⁷** |
| cg09708084 | 0.373 | 0.217 | 0.641 | 3.555 × 10^-^⁰⁴ | 3.732 × 10^-^⁰³ | 0.743 | 0.522 | 1.059 | 1.001 × 10^-^⁰¹ | 0.577 | 0.424 | 0.786 | 4.903 × 10^-^⁰⁴ |
| cg08940001 | 3.964 | 0.001 | 11601.365 | 7.352 × 10^-^⁰¹ | 9.583 × 10^-^⁰¹ | 8705.622 | 20.356 | 3723102.5 | 3.337 × 10^-^⁰³ | 661.561 | 2.688 | 162802.64 | 2.078 × 10^-^⁰² |
| cg24321774 | 1.635 | 1.11 | 2.408 | 1.278 × 10^-^⁰² | 8.947 × 10^-^⁰² | 1.413 | 0.596 | 3.347 | 4.321 × 10^-^⁰¹ | 1.486 | 1.062 | 2.079 | 2.086 × 10^-^⁰² |
| cg25377468 | 1.565 | 0.459 | 5.33 | 4.738 × 10^-^⁰¹ | 7.904 × 10^-^⁰¹ | 4.573 | 0.61 | 34.279 | 1.392 × 10^-^⁰¹ | 2.864 | 0.714 | 11.485 | 1.377 × 10^-^⁰¹ |
| cg11153596 | 0.764 | 0.405 | 1.443 | 4.069 × 10^-^⁰¹ | 7.768 × 10^-^⁰¹ | 0.65 | 0.297 | 1.422 | 2.809 × 10^-^⁰¹ | 0.718 | 0.439 | 1.174 | 1.865 × 10^-^⁰¹ |
| cg07495389 | 0.899 | 0.794 | 1.018 | 9.189 × 10^-^⁰² | 3.859 × 10^-^⁰¹ | 0.956 | 0.845 | 1.08 | 4.672 × 10^-^⁰¹ | 0.949 | 0.87 | 1.034 | 2.319 × 10^-^⁰¹ |
| cg03754074 | 0.903 | 0.448 | 1.819 | 7.758 × 10^-^⁰¹ | 9.583 × 10^-^⁰¹ | 0.598 | 0.251 | 1.426 | 2.459 × 10^-^⁰¹ | 0.723 | 0.422 | 1.237 | 2.365 × 10^-^⁰¹ |
| cg25515091 | 0.977 | 0.678 | 1.41 | 9.027 × 10^-^⁰¹ | 9.630 × 10^-^⁰¹ | 0.594 | 0.369 | 0.957 | 3.214 × 10^-^⁰² | 0.835 | 0.619 | 1.126 | 2.369 × 10^-^⁰¹ |
| cg15315096 | 0.042 | 0.003 | 0.611 | 2.025 × 10^-^⁰² | 1.063 × 10^-^⁰¹ | 2.247 | 0.237 | 21.324 | 4.808 × 10^-^⁰¹ | 0.342 | 0.042 | 2.79 | 3.164 × 10^-^⁰¹ |
| cg25029561 | 0.788 | 0.559 | 1.11 | 1.735 × 10^-^⁰¹ | 6.071 × 10^-^⁰¹ | 1.077 | 0.74 | 1.568 | 6.977 × 10^-^⁰¹ | 0.922 | 0.731 | 1.162 | 4.902 × 10^-^⁰¹ |
| cg17192247 | 0.98 | 0.811 | 1.184 | 8.327 × 10^-^⁰¹ | 9.630 × 10^-^⁰¹ | 1.104 | 0.946 | 1.288 | 2.098 × 10^-^⁰¹ | 1.038 | 0.929 | 1.16 | 5.066 × 10^-^⁰¹ |
| cg01925962 | 0.031 | 0 | 27.38 | 3.149 × 10^-^⁰¹ | 7.348 × 10^-^⁰¹ | 3.435 | 0.002 | 5991.458 | 7.459 × 10^-^⁰¹ | 0.199 | 0.001 | 39.992 | 5.504 × 10^-^⁰¹ |
| cg25781926 | 1.111 | 0.912 | 1.353 | 2.953 × 10^-^⁰¹ | 7.348 × 10^-^⁰¹ | 1.005 | 0.791 | 1.277 | 9.694 × 10^-^⁰¹ | 1.05 | 0.893 | 1.236 | 5.539 × 10^-^⁰¹ |
| cg04400587 | 0.86 | 0.038 | 19.589 | 9.245 × 10^-^⁰¹ | 9.630 × 10^-^⁰¹ | 0.292 | 0.002 | 37.691 | 6.194 × 10^-^⁰¹ | 0.474 | 0.034 | 6.527 | 5.767 × 10^-^⁰¹ |
| cg18625303 | 0.807 | 0.514 | 1.266 | 3.507 × 10^-^⁰¹ | 7.364 × 10^-^⁰¹ | 1.092 | 0.593 | 2.01 | 7.786 × 10^-^⁰¹ | 0.91 | 0.645 | 1.284 | 5.925 × 10^-^⁰¹ |
| cg16212114 | 0.362 | 0.073 | 1.808 | 2.159 × 10^-^⁰¹ | 6.477 × 10^-^⁰¹ | 1.065 | 0.001 | 1511.232 | 9.865 × 10^-^⁰¹ | 0.574 | 0.067 | 4.942 | 6.130 × 10^-^⁰¹ |
| cg06350345 | 0.923 | 0.736 | 1.158 | 4.893 × 10^-^⁰¹ | 7.904 × 10^-^⁰¹ | 1.089 | 0.9 | 1.317 | 3.816 × 10^-^⁰¹ | 1.033 | 0.91 | 1.174 | 6.161 × 10^-^⁰¹ |
| cg08791692 | 1.925 | 0.09 | 41.338 | 6.756 × 10^-^⁰¹ | 9.458 × 10^-^⁰¹ | 2.347 | 0.099 | 55.847 | 5.977 × 10^-^⁰¹ | 1.707 | 0.178 | 16.334 | 6.428 × 10^-^⁰¹ |
| cg04059696 | 1.003 | 0.875 | 1.15 | 9.630 × 10^-^⁰¹ | 9.630 × 10^-^⁰¹ | 0.933 | 0.792 | 1.101 | 4.122 × 10^-^⁰¹ | 0.982 | 0.887 | 1.086 | 7.181 × 10^-^⁰¹ |
| cg09337677 | 0.613 | 0.074 | 5.076 | 6.503 × 10^-^⁰¹ | 9.458 × 10^-^⁰¹ | 16.412 | 0.005 | 56363.30 | 5.006 × 10^-^⁰¹ | 1.498 | 0.087 | 25.808 | 7.808 × 10^-^⁰¹ |

HR: hazard ratio; 95% CI: 95% confidence interval.

**Table S5.** Results of proportional hazards test for cg12821679 in LUSC samples. The CpG probe was treated as a continuous variable in the model.

| CpG probe | Discovery phase | | Validation phase | | Combined dataset | |
| --- | --- | --- | --- | --- | --- | --- |
|  | *χ*^2^ | *P* | *χ*^2^ | *P* | *χ*^2^ | *P* |
| cg12821679 | 0.289 | 0.59 | 3.674 | 0.06 | 1.890 | 0.17 |

**Table S6.** Results of proportional hazards test for cg12821679 in LUSC samples. Patients were stratified into high and low methylation groups based on the median methylation level of the cg12821679*_MAPRE3_* and the CpG probe was treated as a binary variable in the model.

| Variable | Combined dataset | |
| --- | --- | --- |
|  | *χ*^2^ | *P* |
| cg12821679 | 0.440 | 0.51 |

**Table S7.** Results of proportional hazards test for *MAPRE3* expression in LUAD and LUSC samples. Patients were stratified into high and low expression groups based on the median *MAPRE3* expression and the *MAPRE3* expression was treated as a binary variable in the model.

| Population | TCGA dataset^*a^ | |
| --- | --- | --- |
|  | *χ*^2^ | *P* |
| LUAD | 0.673 | 0.412 |
| LUSC | 0.021 | 0.885 |

^*a^ Gene expression analyses were conducted only in TCGA dataset.

**Table S8.** Results of trans-regulation analysis of the significant 1299 genes associated with cg12821679*_MAPRE3_* in TCGA NSCLC samples.

| Gene | Correlation | LCI | UCI | *P^*a^* | FDR | *P^*b^* |
| --- | --- | --- | --- | --- | --- | --- |
| *SPANXN4* | -0.211 | -0.313 | -0.104 | 1.326 × 10⁻⁰⁴ | 1.012 × 10⁻⁰² | 1.735 × 10⁻¹¹ |
| *OR4F6* | -0.175 | -0.279 | -0.067 | 1.570 × 10⁻⁰³ | 3.229 × 10⁻⁰² | 1.636 × 10⁻⁰⁷ |
| *OR10J1* | -0.182 | -0.285 | -0.074 | 1.012 × 10⁻⁰³ | 2.529 × 10⁻⁰² | 1.870 × 10⁻⁰⁷ |
| *OR5AN1* | -0.197 | -0.299 | -0.090 | 3.693 × 10⁻⁰⁴ | 1.488 × 10⁻⁰² | 2.961 × 10⁻⁰⁷ |
| *X..15* | -0.198 | -0.300 | -0.091 | 3.425 × 10⁻⁰⁴ | 1.433 × 10⁻⁰² | 7.210 × 10⁻⁰⁷ |
| *HIST1H2AA* | -0.198 | -0.300 | -0.091 | 3.425 × 10⁻⁰⁴ | 1.433 × 10⁻⁰² | 7.210 × 10⁻⁰⁷ |
| *OR1S1* | -0.198 | -0.300 | -0.091 | 3.425 × 10⁻⁰⁴ | 1.433 × 10⁻⁰² | 7.210 × 10⁻⁰⁷ |
| *OR2M7* | -0.198 | -0.300 | -0.091 | 3.425 × 10⁻⁰⁴ | 1.433 × 10⁻⁰² | 7.210 × 10⁻⁰⁷ |
| *OR2T27* | -0.198 | -0.300 | -0.091 | 3.425 × 10⁻⁰⁴ | 1.433 × 10⁻⁰² | 7.210 × 10⁻⁰⁷ |
| *OR4A5* | -0.198 | -0.300 | -0.091 | 3.425 × 10⁻⁰⁴ | 1.433 × 10⁻⁰² | 7.210 × 10⁻⁰⁷ |
| *OR4D11* | -0.198 | -0.300 | -0.091 | 3.425 × 10⁻⁰⁴ | 1.433 × 10⁻⁰² | 7.210 × 10⁻⁰⁷ |
| *OR4K5* | -0.198 | -0.300 | -0.091 | 3.425 × 10⁻⁰⁴ | 1.433 × 10⁻⁰² | 7.210 × 10⁻⁰⁷ |
| *OR5AR1* | -0.198 | -0.300 | -0.091 | 3.425 × 10⁻⁰⁴ | 1.433 × 10⁻⁰² | 7.210 × 10⁻⁰⁷ |
| *OR5D16* | -0.198 | -0.300 | -0.091 | 3.425 × 10⁻⁰⁴ | 1.433 × 10⁻⁰² | 7.210 × 10⁻⁰⁷ |
| *OR5H15* | -0.198 | -0.300 | -0.091 | 3.425 × 10⁻⁰⁴ | 1.433 × 10⁻⁰² | 7.210 × 10⁻⁰⁷ |
| *OR8J1* | -0.198 | -0.300 | -0.091 | 3.425 × 10⁻⁰⁴ | 1.433 × 10⁻⁰² | 7.210 × 10⁻⁰⁷ |
| *OR2T35* | -0.198 | -0.300 | -0.091 | 3.425 × 10⁻⁰⁴ | 1.433 × 10⁻⁰² | 7.210 × 10⁻⁰⁷ |
| *OR4B1* | -0.198 | -0.300 | -0.091 | 3.425 × 10⁻⁰⁴ | 1.433 × 10⁻⁰² | 7.210 × 10⁻⁰⁷ |
| *OR4C45* | -0.198 | -0.300 | -0.091 | 3.425 × 10⁻⁰⁴ | 1.433 × 10⁻⁰² | 7.210 × 10⁻⁰⁷ |
| *OR5W2* | -0.198 | -0.300 | -0.091 | 3.425 × 10⁻⁰⁴ | 1.433 × 10⁻⁰² | 7.210 × 10⁻⁰⁷ |
| *SNORD115.7* | -0.198 | -0.300 | -0.091 | 3.425 × 10⁻⁰⁴ | 1.433 × 10⁻⁰² | 7.210 × 10⁻⁰⁷ |
| *OR10Z1* | -0.198 | -0.300 | -0.091 | 3.425 × 10⁻⁰⁴ | 1.433 × 10⁻⁰² | 7.210 × 10⁻⁰⁷ |
| *OR2T1* | -0.198 | -0.300 | -0.091 | 3.425 × 10⁻⁰⁴ | 1.433 × 10⁻⁰² | 7.210 × 10⁻⁰⁷ |
| *OR4K2* | -0.198 | -0.300 | -0.091 | 3.425 × 10⁻⁰⁴ | 1.433 × 10⁻⁰² | 7.210 × 10⁻⁰⁷ |
| *OR5B3* | -0.198 | -0.300 | -0.091 | 3.425 × 10⁻⁰⁴ | 1.433 × 10⁻⁰² | 7.210 × 10⁻⁰⁷ |
| *OR6C4* | -0.198 | -0.300 | -0.091 | 3.425 × 10⁻⁰⁴ | 1.433 × 10⁻⁰² | 7.210 × 10⁻⁰⁷ |
| *OR6Y1* | -0.198 | -0.300 | -0.091 | 3.425 × 10⁻⁰⁴ | 1.433 × 10⁻⁰² | 7.210 × 10⁻⁰⁷ |
| *ATXN8OS* | -0.198 | -0.300 | -0.091 | 3.425 × 10⁻⁰⁴ | 1.433 × 10⁻⁰² | 7.210 × 10⁻⁰⁷ |
| *C11orf40* | -0.198 | -0.300 | -0.091 | 3.425 × 10⁻⁰⁴ | 1.433 × 10⁻⁰² | 7.210 × 10⁻⁰⁷ |
| *DEFB127* | -0.198 | -0.300 | -0.091 | 3.425 × 10⁻⁰⁴ | 1.433 × 10⁻⁰² | 7.210 × 10⁻⁰⁷ |
| *KRTAP12.4* | -0.198 | -0.300 | -0.091 | 3.425 × 10⁻⁰⁴ | 1.433 × 10⁻⁰² | 7.210 × 10⁻⁰⁷ |
| *OR8I2* | -0.198 | -0.300 | -0.091 | 3.425 × 10⁻⁰⁴ | 1.433 × 10⁻⁰² | 7.210 × 10⁻⁰⁷ |
| *OR8J3* | -0.198 | -0.300 | -0.091 | 3.425 × 10⁻⁰⁴ | 1.433 × 10⁻⁰² | 7.210 × 10⁻⁰⁷ |
| *LOC730811* | -0.222 | -0.323 | -0.116 | 5.536 × 10⁻⁰⁵ | 6.189 × 10⁻⁰³ | 7.210 × 10⁻⁰⁷ |
| *OR1A1* | -0.198 | -0.300 | -0.091 | 3.425 × 10⁻⁰⁴ | 1.433 × 10⁻⁰² | 7.210 × 10⁻⁰⁷ |
| *OR2Y1* | -0.198 | -0.300 | -0.091 | 3.425 × 10⁻⁰⁴ | 1.433 × 10⁻⁰² | 7.210 × 10⁻⁰⁷ |
| *OR4C13* | -0.198 | -0.300 | -0.091 | 3.425 × 10⁻⁰⁴ | 1.433 × 10⁻⁰² | 7.210 × 10⁻⁰⁷ |
| *OR4K13* | -0.198 | -0.300 | -0.091 | 3.425 × 10⁻⁰⁴ | 1.433 × 10⁻⁰² | 7.210 × 10⁻⁰⁷ |
| *OR5L2* | -0.198 | -0.300 | -0.091 | 3.425 × 10⁻⁰⁴ | 1.433 × 10⁻⁰² | 7.210 × 10⁻⁰⁷ |
| *TTTY12* | -0.198 | -0.300 | -0.091 | 3.425 × 10⁻⁰⁴ | 1.433 × 10⁻⁰² | 7.210 × 10⁻⁰⁷ |
| *TAAR5* | -0.198 | -0.300 | -0.091 | 3.425 × 10⁻⁰⁴ | 1.433 × 10⁻⁰² | 7.210 × 10⁻⁰⁷ |
| *OR4C46* | -0.198 | -0.300 | -0.091 | 3.425 × 10⁻⁰⁴ | 1.433 × 10⁻⁰² | 7.210 × 10⁻⁰⁷ |
| *OR51S1* | -0.198 | -0.300 | -0.091 | 3.425 × 10⁻⁰⁴ | 1.433 × 10⁻⁰² | 7.210 × 10⁻⁰⁷ |
| *OR5AS1* | -0.198 | -0.300 | -0.091 | 3.425 × 10⁻⁰⁴ | 1.433 × 10⁻⁰² | 7.210 × 10⁻⁰⁷ |
| *OR5I1* | -0.198 | -0.300 | -0.091 | 3.425 × 10⁻⁰⁴ | 1.433 × 10⁻⁰² | 7.210 × 10⁻⁰⁷ |
| *OR7G1* | -0.198 | -0.300 | -0.091 | 3.425 × 10⁻⁰⁴ | 1.433 × 10⁻⁰² | 7.210 × 10⁻⁰⁷ |
| *OR8K1* | -0.198 | -0.300 | -0.091 | 3.425 × 10⁻⁰⁴ | 1.433 × 10⁻⁰² | 7.210 × 10⁻⁰⁷ |
| *IFNA6* | -0.198 | -0.300 | -0.091 | 3.425 × 10⁻⁰⁴ | 1.433 × 10⁻⁰² | 7.210 × 10⁻⁰⁷ |
| *KRTAP15.1* | -0.198 | -0.300 | -0.091 | 3.425 × 10⁻⁰⁴ | 1.433 × 10⁻⁰² | 7.210 × 10⁻⁰⁷ |
| *LCE4A* | -0.198 | -0.300 | -0.091 | 3.425 × 10⁻⁰⁴ | 1.433 × 10⁻⁰² | 7.210 × 10⁻⁰⁷ |
| *OR2T34* | -0.198 | -0.300 | -0.091 | 3.425 × 10⁻⁰⁴ | 1.433 × 10⁻⁰² | 7.210 × 10⁻⁰⁷ |
| *PISRT1* | -0.198 | -0.300 | -0.091 | 3.425 × 10⁻⁰⁴ | 1.433 × 10⁻⁰² | 7.210 × 10⁻⁰⁷ |
| *OR10X1* | -0.198 | -0.300 | -0.091 | 3.425 × 10⁻⁰⁴ | 1.433 × 10⁻⁰² | 7.210 × 10⁻⁰⁷ |
| *OR51F1* | -0.198 | -0.300 | -0.091 | 3.425 × 10⁻⁰⁴ | 1.433 × 10⁻⁰² | 7.210 × 10⁻⁰⁷ |
| *OR5F1* | -0.198 | -0.300 | -0.091 | 3.425 × 10⁻⁰⁴ | 1.433 × 10⁻⁰² | 7.210 × 10⁻⁰⁷ |
| *OR5M9* | -0.198 | -0.300 | -0.091 | 3.425 × 10⁻⁰⁴ | 1.433 × 10⁻⁰² | 7.210 × 10⁻⁰⁷ |
| *OR5R1* | -0.198 | -0.300 | -0.091 | 3.425 × 10⁻⁰⁴ | 1.433 × 10⁻⁰² | 7.210 × 10⁻⁰⁷ |
| *OR6C74* | -0.198 | -0.300 | -0.091 | 3.425 × 10⁻⁰⁴ | 1.433 × 10⁻⁰² | 7.210 × 10⁻⁰⁷ |
| *OR6K2* | -0.198 | -0.300 | -0.091 | 3.425 × 10⁻⁰⁴ | 1.433 × 10⁻⁰² | 7.210 × 10⁻⁰⁷ |
| *TAAR2* | -0.198 | -0.300 | -0.091 | 3.425 × 10⁻⁰⁴ | 1.433 × 10⁻⁰² | 7.210 × 10⁻⁰⁷ |
| *FKSG73* | -0.198 | -0.300 | -0.091 | 3.425 × 10⁻⁰⁴ | 1.433 × 10⁻⁰² | 7.210 × 10⁻⁰⁷ |
| *OR10K1* | -0.198 | -0.300 | -0.091 | 3.425 × 10⁻⁰⁴ | 1.433 × 10⁻⁰² | 7.210 × 10⁻⁰⁷ |
| *OR1S2* | -0.198 | -0.300 | -0.091 | 3.425 × 10⁻⁰⁴ | 1.433 × 10⁻⁰² | 7.210 × 10⁻⁰⁷ |
| *OR2T6* | -0.198 | -0.300 | -0.091 | 3.425 × 10⁻⁰⁴ | 1.433 × 10⁻⁰² | 7.210 × 10⁻⁰⁷ |
| *OR4X1* | -0.198 | -0.300 | -0.091 | 3.425 × 10⁻⁰⁴ | 1.433 × 10⁻⁰² | 7.210 × 10⁻⁰⁷ |
| *OR52A1* | -0.198 | -0.300 | -0.091 | 3.425 × 10⁻⁰⁴ | 1.433 × 10⁻⁰² | 7.210 × 10⁻⁰⁷ |
| *OR6Q1* | -0.197 | -0.300 | -0.090 | 3.511 × 10⁻⁰⁴ | 1.447 × 10⁻⁰² | 7.210 × 10⁻⁰⁷ |
| *LOC151300* | -0.218 | -0.320 | -0.112 | 7.420 × 10⁻⁰⁵ | 7.392 × 10⁻⁰³ | 7.245 × 10⁻⁰⁷ |
| *BIRC8* | -0.191 | -0.294 | -0.084 | 5.384 × 10⁻⁰⁴ | 1.789 × 10⁻⁰² | 7.254 × 10⁻⁰⁷ |
| *KRTAP4.3* | -0.199 | -0.301 | -0.092 | 3.158 × 10⁻⁰⁴ | 1.433 × 10⁻⁰² | 7.359 × 10⁻⁰⁷ |
| *KRTAP10.9* | -0.193 | -0.296 | -0.086 | 4.695 × 10⁻⁰⁴ | 1.690 × 10⁻⁰² | 7.394 × 10⁻⁰⁷ |
| *OR4F17* | -0.263 | -0.362 | -0.159 | 1.555 × 10⁻⁰⁶ | 7.318 × 10⁻⁰⁴ | 7.547 × 10⁻⁰⁷ |
| *SPATA3* | -0.185 | -0.288 | -0.078 | 8.049 × 10⁻⁰⁴ | 2.231 × 10⁻⁰² | 7.710 × 10⁻⁰⁷ |
| *OR5B17* | -0.196 | -0.299 | -0.089 | 3.780 × 10⁻⁰⁴ | 1.511 × 10⁻⁰² | 7.749 × 10⁻⁰⁷ |
| *TRIM60* | -0.201 | -0.303 | -0.094 | 2.760 × 10⁻⁰⁴ | 1.433 × 10⁻⁰² | 7.772 × 10⁻⁰⁷ |
| *OR5D13* | -0.198 | -0.300 | -0.091 | 3.463 × 10⁻⁰⁴ | 1.436 × 10⁻⁰² | 7.772 × 10⁻⁰⁷ |
| *LOC729121* | -0.192 | -0.295 | -0.085 | 5.194 × 10⁻⁰⁴ | 1.768 × 10⁻⁰² | 7.793 × 10⁻⁰⁷ |
| *OR7A10* | -0.194 | -0.297 | -0.087 | 4.396 × 10⁻⁰⁴ | 1.641 × 10⁻⁰² | 7.799 × 10⁻⁰⁷ |
| *DEFB118* | -0.192 | -0.295 | -0.085 | 4.950 × 10⁻⁰⁴ | 1.726 × 10⁻⁰² | 7.841 × 10⁻⁰⁷ |
| *OR10T2* | -0.209 | -0.310 | -0.102 | 1.561 × 10⁻⁰⁴ | 1.104 × 10⁻⁰² | 7.895 × 10⁻⁰⁷ |
| *OR4D9* | -0.190 | -0.293 | -0.083 | 5.805 × 10⁻⁰⁴ | 1.868 × 10⁻⁰² | 8.043 × 10⁻⁰⁷ |
| *OR2G3* | -0.188 | -0.291 | -0.081 | 6.506 × 10⁻⁰⁴ | 1.989 × 10⁻⁰² | 8.131 × 10⁻⁰⁷ |
| *CDY1* | -0.172 | -0.276 | -0.064 | 1.886 × 10⁻⁰³ | 3.608 × 10⁻⁰² | 8.223 × 10⁻⁰⁷ |
| *C20orf71* | -0.184 | -0.287 | -0.076 | 8.814 × 10⁻⁰⁴ | 2.312 × 10⁻⁰² | 8.560 × 10⁻⁰⁷ |
| *OR4C15* | -0.203 | -0.306 | -0.097 | 2.274 × 10⁻⁰⁴ | 1.315 × 10⁻⁰² | 8.730 × 10⁻⁰⁷ |
| *PRL* | -0.170 | -0.274 | -0.062 | 2.105 × 10⁻⁰³ | 3.879 × 10⁻⁰² | 8.741 × 10⁻⁰⁷ |
| *TMEM225* | -0.239 | -0.340 | -0.134 | 1.323 × 10⁻⁰⁵ | 2.651 × 10⁻⁰³ | 8.795 × 10⁻⁰⁷ |
| *OR4C3* | -0.194 | -0.297 | -0.087 | 4.311 × 10⁻⁰⁴ | 1.624 × 10⁻⁰² | 9.209 × 10⁻⁰⁷ |
| *OR10K2* | -0.247 | -0.347 | -0.142 | 6.767 × 10⁻⁰⁶ | 2.006 × 10⁻⁰³ | 9.216 × 10⁻⁰⁷ |
| *OR5H1* | -0.199 | -0.302 | -0.092 | 3.068 × 10⁻⁰⁴ | 1.433 × 10⁻⁰² | 9.249 × 10⁻⁰⁷ |
| *IFNA16* | -0.191 | -0.294 | -0.084 | 5.350 × 10⁻⁰⁴ | 1.783 × 10⁻⁰² | 9.281 × 10⁻⁰⁷ |
| *LOC646813* | -0.189 | -0.291 | -0.081 | 6.483 × 10⁻⁰⁴ | 1.988 × 10⁻⁰² | 9.333 × 10⁻⁰⁷ |
| *REG1P* | -0.168 | -0.272 | -0.060 | 2.479 × 10⁻⁰³ | 4.289 × 10⁻⁰² | 9.485 × 10⁻⁰⁷ |
| *OR4S1* | -0.196 | -0.298 | -0.089 | 3.904 × 10⁻⁰⁴ | 1.525 × 10⁻⁰² | 9.551 × 10⁻⁰⁷ |
| *OR6P1* | -0.203 | -0.305 | -0.096 | 2.386 × 10⁻⁰⁴ | 1.334 × 10⁻⁰² | 9.830 × 10⁻⁰⁷ |
| *OR10A7* | -0.242 | -0.342 | -0.137 | 1.049 × 10⁻⁰⁵ | 2.454 × 10⁻⁰³ | 9.911 × 10⁻⁰⁷ |
| *IQCF5* | -0.227 | -0.328 | -0.121 | 3.588 × 10⁻⁰⁵ | 4.776 × 10⁻⁰³ | 9.934 × 10⁻⁰⁷ |
| *OR8H2* | -0.197 | -0.299 | -0.090 | 3.724 × 10⁻⁰⁴ | 1.495 × 10⁻⁰² | 1.010 × 10⁻⁰⁶ |
| *OR8B8* | -0.198 | -0.300 | -0.091 | 3.377 × 10⁻⁰⁴ | 1.433 × 10⁻⁰² | 1.019 × 10⁻⁰⁶ |
| *OR2M5* | -0.196 | -0.299 | -0.089 | 3.786 × 10⁻⁰⁴ | 1.511 × 10⁻⁰² | 1.041 × 10⁻⁰⁶ |
| *CSHL1* | -0.202 | -0.304 | -0.095 | 2.604 × 10⁻⁰⁴ | 1.412 × 10⁻⁰² | 1.073 × 10⁻⁰⁶ |
| *OR8B4* | -0.192 | -0.295 | -0.085 | 5.117 × 10⁻⁰⁴ | 1.760 × 10⁻⁰² | 1.130 × 10⁻⁰⁶ |
| *OR10G9* | -0.249 | -0.348 | -0.144 | 5.786 × 10⁻⁰⁶ | 1.830 × 10⁻⁰³ | 1.138 × 10⁻⁰⁶ |
| *OR4K15* | -0.174 | -0.278 | -0.067 | 1.637 × 10⁻⁰³ | 3.322 × 10⁻⁰² | 1.166 × 10⁻⁰⁶ |
| *OR1I1* | -0.248 | -0.348 | -0.143 | 6.240 × 10⁻⁰⁶ | 1.943 × 10⁻⁰³ | 1.176 × 10⁻⁰⁶ |
| *HSFYL1* | -0.190 | -0.292 | -0.082 | 6.037 × 10⁻⁰⁴ | 1.911 × 10⁻⁰² | 1.254 × 10⁻⁰⁶ |
| *OR10G3* | -0.193 | -0.296 | -0.086 | 4.729 × 10⁻⁰⁴ | 1.690 × 10⁻⁰² | 1.372 × 10⁻⁰⁶ |
| *OR5A1* | -0.221 | -0.322 | -0.115 | 5.904 × 10⁻⁰⁵ | 6.480 × 10⁻⁰³ | 1.420 × 10⁻⁰⁶ |
| *OR5A2* | -0.164 | -0.268 | -0.056 | 3.020 × 10⁻⁰³ | 4.816 × 10⁻⁰² | 1.422 × 10⁻⁰⁶ |
| *OR4C12* | -0.193 | -0.296 | -0.086 | 4.787 × 10⁻⁰⁴ | 1.696 × 10⁻⁰² | 1.512 × 10⁻⁰⁶ |
| *OR51V1* | -0.193 | -0.296 | -0.086 | 4.625 × 10⁻⁰⁴ | 1.683 × 10⁻⁰² | 1.779 × 10⁻⁰⁶ |
| *POM121L12* | -0.204 | -0.306 | -0.097 | 2.158 × 10⁻⁰⁴ | 1.273 × 10⁻⁰² | 1.849 × 10⁻⁰⁶ |
| *OR3A4* | -0.242 | -0.342 | -0.137 | 1.055 × 10⁻⁰⁵ | 2.454 × 10⁻⁰³ | 1.872 × 10⁻⁰⁶ |
| *KRTAP10.12* | -0.190 | -0.293 | -0.082 | 5.965 × 10⁻⁰⁴ | 1.901 × 10⁻⁰² | 1.940 × 10⁻⁰⁶ |
| *CYP11B2* | -0.185 | -0.288 | -0.077 | 8.405 × 10⁻⁰⁴ | 2.274 × 10⁻⁰² | 2.344 × 10⁻⁰⁶ |
| *OR11L1* | -0.232 | -0.332 | -0.126 | 2.485 × 10⁻⁰⁵ | 3.868 × 10⁻⁰³ | 2.513 × 10⁻⁰⁶ |
| *GPR139* | -0.223 | -0.324 | -0.116 | 5.286 × 10⁻⁰⁵ | 5.976 × 10⁻⁰³ | 2.653 × 10⁻⁰⁶ |
| *OR4C16* | -0.197 | -0.300 | -0.090 | 3.547 × 10⁻⁰⁴ | 1.454 × 10⁻⁰² | 3.004 × 10⁻⁰⁶ |
| *OR10H4* | -0.192 | -0.294 | -0.084 | 5.250 × 10⁻⁰⁴ | 1.771 × 10⁻⁰² | 3.116 × 10⁻⁰⁶ |
| *OR2Z1* | -0.189 | -0.292 | -0.082 | 6.106 × 10⁻⁰⁴ | 1.919 × 10⁻⁰² | 3.374 × 10⁻⁰⁶ |
| *C20orf79* | -0.185 | -0.289 | -0.078 | 7.941 × 10⁻⁰⁴ | 2.223 × 10⁻⁰² | 3.430 × 10⁻⁰⁶ |
| *OR4F4* | -0.211 | -0.313 | -0.105 | 1.262 × 10⁻⁰⁴ | 9.785 × 10⁻⁰³ | 3.455 × 10⁻⁰⁶ |
| *SPANXN1* | -0.203 | -0.305 | -0.096 | 2.314 × 10⁻⁰⁴ | 1.319 × 10⁻⁰² | 3.466 × 10⁻⁰⁶ |
| *OR8U1* | -0.199 | -0.301 | -0.092 | 3.217 × 10⁻⁰⁴ | 1.433 × 10⁻⁰² | 3.525 × 10⁻⁰⁶ |
| *OR5AC2* | -0.176 | -0.280 | -0.068 | 1.460 × 10⁻⁰³ | 3.113 × 10⁻⁰² | 4.249 × 10⁻⁰⁶ |
| *OR51D1* | -0.204 | -0.306 | -0.097 | 2.165 × 10⁻⁰⁴ | 1.274 × 10⁻⁰² | 4.718 × 10⁻⁰⁶ |
| *OR51F2* | -0.207 | -0.309 | -0.100 | 1.786 × 10⁻⁰⁴ | 1.167 × 10⁻⁰² | 4.770 × 10⁻⁰⁶ |
| *OR2L8* | -0.198 | -0.300 | -0.091 | 3.472 × 10⁻⁰⁴ | 1.437 × 10⁻⁰² | 4.948 × 10⁻⁰⁶ |
| *OR7D4* | -0.186 | -0.289 | -0.079 | 7.709 × 10⁻⁰⁴ | 2.206 × 10⁻⁰² | 5.475 × 10⁻⁰⁶ |
| *OR1D2* | -0.228 | -0.328 | -0.122 | 3.568 × 10⁻⁰⁵ | 4.776 × 10⁻⁰³ | 5.563 × 10⁻⁰⁶ |
| *OR12D3* | -0.190 | -0.293 | -0.083 | 5.713 × 10⁻⁰⁴ | 1.850 × 10⁻⁰² | 5.886 × 10⁻⁰⁶ |
| *FLJ43859* | -0.189 | -0.292 | -0.082 | 6.108 × 10⁻⁰⁴ | 1.919 × 10⁻⁰² | 5.888 × 10⁻⁰⁶ |
| *OR52A5* | -0.197 | -0.299 | -0.090 | 3.647 × 10⁻⁰⁴ | 1.476 × 10⁻⁰² | 6.005 × 10⁻⁰⁶ |
| *OR2W5* | -0.205 | -0.307 | -0.099 | 1.985 × 10⁻⁰⁴ | 1.203 × 10⁻⁰² | 7.341 × 10⁻⁰⁶ |
| *TAAR8* | -0.201 | -0.304 | -0.094 | 2.656 × 10⁻⁰⁴ | 1.430 × 10⁻⁰² | 7.607 × 10⁻⁰⁶ |
| *OR10G8* | -0.191 | -0.294 | -0.084 | 5.568 × 10⁻⁰⁴ | 1.821 × 10⁻⁰² | 8.647 × 10⁻⁰⁶ |
| *OR10W1* | -0.194 | -0.297 | -0.087 | 4.376 × 10⁻⁰⁴ | 1.640 × 10⁻⁰² | 9.457 × 10⁻⁰⁶ |
| *OR10J5* | -0.193 | -0.296 | -0.086 | 4.698 × 10⁻⁰⁴ | 1.690 × 10⁻⁰² | 1.114 × 10⁻⁰⁵ |
| *OR51A7* | -0.200 | -0.302 | -0.093 | 3.005 × 10⁻⁰⁴ | 1.433 × 10⁻⁰² | 1.159 × 10⁻⁰⁵ |
| *OR8B12* | -0.233 | -0.334 | -0.128 | 2.216 × 10⁻⁰⁵ | 3.531 × 10⁻⁰³ | 1.479 × 10⁻⁰⁵ |
| *OR14C36* | -0.206 | -0.308 | -0.099 | 1.911 × 10⁻⁰⁴ | 1.194 × 10⁻⁰² | 1.623 × 10⁻⁰⁵ |
| *OR8H3* | -0.201 | -0.303 | -0.094 | 2.709 × 10⁻⁰⁴ | 1.433 × 10⁻⁰² | 1.713 × 10⁻⁰⁵ |
| *C18orf62* | -0.231 | -0.331 | -0.125 | 2.756 × 10⁻⁰⁵ | 4.162 × 10⁻⁰³ | 1.782 × 10⁻⁰⁵ |
| *OR4D6* | -0.205 | -0.307 | -0.098 | 2.048 × 10⁻⁰⁴ | 1.233 × 10⁻⁰² | 1.796 × 10⁻⁰⁵ |
| *X..16* | -0.213 | -0.314 | -0.106 | 1.151 × 10⁻⁰⁴ | 9.232 × 10⁻⁰³ | 2.039 × 10⁻⁰⁵ |
| *OR9G4* | -0.189 | -0.292 | -0.082 | 6.230 × 10⁻⁰⁴ | 1.934 × 10⁻⁰² | 2.148 × 10⁻⁰⁵ |
| *C16orf82* | -0.224 | -0.325 | -0.118 | 4.703 × 10⁻⁰⁵ | 5.597 × 10⁻⁰³ | 2.163 × 10⁻⁰⁵ |
| *IFNA14* | -0.236 | -0.336 | -0.130 | 1.834 × 10⁻⁰⁵ | 3.284 × 10⁻⁰³ | 2.169 × 10⁻⁰⁵ |
| *OR10G7* | -0.177 | -0.281 | -0.069 | 1.379 × 10⁻⁰³ | 3.042 × 10⁻⁰² | 2.269 × 10⁻⁰⁵ |
| *OR51L1* | -0.206 | -0.308 | -0.100 | 1.851 × 10⁻⁰⁴ | 1.185 × 10⁻⁰² | 2.373 × 10⁻⁰⁵ |
| *PAR4* | -0.206 | -0.308 | -0.100 | 1.833 × 10⁻⁰⁴ | 1.179 × 10⁻⁰² | 2.719 × 10⁻⁰⁵ |
| *MGC26647* | -0.184 | -0.288 | -0.077 | 8.513 × 10⁻⁰⁴ | 2.282 × 10⁻⁰² | 3.305 × 10⁻⁰⁵ |
| *OR9I1* | -0.170 | -0.274 | -0.062 | 2.163 × 10⁻⁰³ | 3.945 × 10⁻⁰² | 3.736 × 10⁻⁰⁵ |
| *OTUD6A* | -0.218 | -0.319 | -0.111 | 7.813 × 10⁻⁰⁵ | 7.453 × 10⁻⁰³ | 3.906 × 10⁻⁰⁵ |
| *KRTAP24.1* | -0.176 | -0.280 | -0.069 | 1.443 × 10⁻⁰³ | 3.094 × 10⁻⁰² | 3.936 × 10⁻⁰⁵ |
| *OR4K17* | -0.201 | -0.304 | -0.095 | 2.637 × 10⁻⁰⁴ | 1.423 × 10⁻⁰² | 3.938 × 10⁻⁰⁵ |
| *FGF6* | -0.185 | -0.288 | -0.077 | 8.461 × 10⁻⁰⁴ | 2.280 × 10⁻⁰² | 4.093 × 10⁻⁰⁵ |
| *PRNT* | -0.190 | -0.293 | -0.083 | 5.746 × 10⁻⁰⁴ | 1.857 × 10⁻⁰² | 4.185 × 10⁻⁰⁵ |
| *OR4F15* | -0.186 | -0.289 | -0.078 | 7.875 × 10⁻⁰⁴ | 2.210 × 10⁻⁰² | 4.251 × 10⁻⁰⁵ |
| *AWAT1* | -0.178 | -0.281 | -0.070 | 1.333 × 10⁻⁰³ | 2.967 × 10⁻⁰² | 4.428 × 10⁻⁰⁵ |
| *OR5J2* | -0.187 | -0.290 | -0.079 | 7.398 × 10⁻⁰⁴ | 2.148 × 10⁻⁰² | 4.620 × 10⁻⁰⁵ |
| *OR5L1* | -0.195 | -0.298 | -0.088 | 4.061 × 10⁻⁰⁴ | 1.561 × 10⁻⁰² | 4.699 × 10⁻⁰⁵ |
| *CXorf66* | -0.169 | -0.273 | -0.061 | 2.307 × 10⁻⁰³ | 4.125 × 10⁻⁰² | 5.061 × 10⁻⁰⁵ |
| *FLJ46321* | -0.189 | -0.292 | -0.082 | 6.064 × 10⁻⁰⁴ | 1.914 × 10⁻⁰² | 5.304 × 10⁻⁰⁵ |
| *TCP10L2* | -0.178 | -0.281 | -0.070 | 1.310 × 10⁻⁰³ | 2.943 × 10⁻⁰² | 5.400 × 10⁻⁰⁵ |
| *CRISP1* | -0.327 | -0.421 | -0.226 | 1.589 × 10⁻⁰⁹ | 5.358 × 10⁻⁰⁶ | 5.656 × 10⁻⁰⁵ |
| *STH* | -0.207 | -0.309 | -0.100 | 1.794 × 10⁻⁰⁴ | 1.167 × 10⁻⁰² | 5.704 × 10⁻⁰⁵ |
| *OR10P1* | -0.177 | -0.280 | -0.069 | 1.393 × 10⁻⁰³ | 3.055 × 10⁻⁰² | 5.831 × 10⁻⁰⁵ |
| *OR52R1* | -0.188 | -0.291 | -0.081 | 6.758 × 10⁻⁰⁴ | 2.042 × 10⁻⁰² | 5.941 × 10⁻⁰⁵ |
| *OR4K14* | -0.181 | -0.285 | -0.074 | 1.046 × 10⁻⁰³ | 2.570 × 10⁻⁰² | 5.990 × 10⁻⁰⁵ |
| *ASB17* | -0.171 | -0.275 | -0.064 | 1.956 × 10⁻⁰³ | 3.710 × 10⁻⁰² | 6.059 × 10⁻⁰⁵ |
| *SPANXN2* | -0.178 | -0.281 | -0.070 | 1.324 × 10⁻⁰³ | 2.953 × 10⁻⁰² | 6.556 × 10⁻⁰⁵ |
| *C19orf75* | -0.207 | -0.309 | -0.100 | 1.814 × 10⁻⁰⁴ | 1.173 × 10⁻⁰² | 6.616 × 10⁻⁰⁵ |
| *OR6X1* | -0.188 | -0.291 | -0.080 | 6.844 × 10⁻⁰⁴ | 2.058 × 10⁻⁰² | 6.680 × 10⁻⁰⁵ |
| *OR2J2* | -0.210 | -0.312 | -0.103 | 1.422 × 10⁻⁰⁴ | 1.035 × 10⁻⁰² | 6.827 × 10⁻⁰⁵ |
| *NEUROD6* | -0.225 | -0.326 | -0.119 | 4.203 × 10⁻⁰⁵ | 5.250 × 10⁻⁰³ | 7.231 × 10⁻⁰⁵ |
| *PTF1A* | -0.189 | -0.292 | -0.082 | 6.179 × 10⁻⁰⁴ | 1.927 × 10⁻⁰² | 7.626 × 10⁻⁰⁵ |
| *OR4L1* | -0.183 | -0.286 | -0.076 | 9.303 × 10⁻⁰⁴ | 2.398 × 10⁻⁰² | 7.950 × 10⁻⁰⁵ |
| *OR52B2* | -0.165 | -0.269 | -0.057 | 2.934 × 10⁻⁰³ | 4.745 × 10⁻⁰² | 8.119 × 10⁻⁰⁵ |
| *OR5M3* | -0.215 | -0.317 | -0.109 | 9.620 × 10⁻⁰⁵ | 8.517 × 10⁻⁰³ | 8.377 × 10⁻⁰⁵ |
| *OR8H1* | -0.210 | -0.312 | -0.104 | 1.376 × 10⁻⁰⁴ | 1.022 × 10⁻⁰² | 8.552 × 10⁻⁰⁵ |
| *IFNA10* | -0.195 | -0.298 | -0.088 | 4.088 × 10⁻⁰⁴ | 1.564 × 10⁻⁰² | 9.417 × 10⁻⁰⁵ |
| *TAS2R39* | -0.185 | -0.288 | -0.078 | 8.240 × 10⁻⁰⁴ | 2.256 × 10⁻⁰² | 9.624 × 10⁻⁰⁵ |
| *HPVC1* | -0.187 | -0.290 | -0.080 | 7.192 × 10⁻⁰⁴ | 2.112 × 10⁻⁰² | 1.182 × 10⁻⁰⁴ |
| *PRAMEF10* | -0.215 | -0.317 | -0.109 | 9.280 × 10⁻⁰⁵ | 8.401 × 10⁻⁰³ | 1.211 × 10⁻⁰⁴ |
| *RNASE8* | -0.208 | -0.310 | -0.101 | 1.629 × 10⁻⁰⁴ | 1.130 × 10⁻⁰² | 1.434 × 10⁻⁰⁴ |
| *IFNA17* | -0.190 | -0.293 | -0.083 | 5.822 × 10⁻⁰⁴ | 1.870 × 10⁻⁰² | 1.668 × 10⁻⁰⁴ |
| *OR2M2* | -0.197 | -0.299 | -0.090 | 3.656 × 10⁻⁰⁴ | 1.477 × 10⁻⁰² | 1.888 × 10⁻⁰⁴ |
| *LRRC10* | -0.283 | -0.381 | -0.180 | 2.143 × 10⁻⁰⁷ | 2.245 × 10⁻⁰⁴ | 1.893 × 10⁻⁰⁴ |
| *OR4X2* | -0.173 | -0.277 | -0.066 | 1.739 × 10⁻⁰³ | 3.448 × 10⁻⁰² | 1.937 × 10⁻⁰⁴ |
| *PWRN2* | -0.228 | -0.329 | -0.123 | 3.296 × 10⁻⁰⁵ | 4.541 × 10⁻⁰³ | 1.944 × 10⁻⁰⁴ |
| *ACTL9* | -0.164 | -0.268 | -0.056 | 3.158 × 10⁻⁰³ | 4.953 × 10⁻⁰² | 1.958 × 10⁻⁰⁴ |
| *KIF2B* | -0.218 | -0.319 | -0.112 | 7.635 × 10⁻⁰⁵ | 7.392 × 10⁻⁰³ | 2.170 × 10⁻⁰⁴ |
| *OR52E4* | -0.183 | -0.286 | -0.076 | 9.354 × 10⁻⁰⁴ | 2.402 × 10⁻⁰² | 2.175 × 10⁻⁰⁴ |
| *FGF4* | -0.172 | -0.276 | -0.064 | 1.914 × 10⁻⁰³ | 3.650 × 10⁻⁰² | 2.252 × 10⁻⁰⁴ |
| *GK2* | -0.176 | -0.280 | -0.068 | 1.467 × 10⁻⁰³ | 3.117 × 10⁻⁰² | 2.314 × 10⁻⁰⁴ |
| *OR6C6* | -0.186 | -0.289 | -0.078 | 7.870 × 10⁻⁰⁴ | 2.210 × 10⁻⁰² | 2.317 × 10⁻⁰⁴ |
| *LOC116437* | -0.207 | -0.309 | -0.101 | 1.710 × 10⁻⁰⁴ | 1.146 × 10⁻⁰² | 2.588 × 10⁻⁰⁴ |
| *KRTAP10.7* | -0.202 | -0.304 | -0.095 | 2.593 × 10⁻⁰⁴ | 1.410 × 10⁻⁰² | 2.631 × 10⁻⁰⁴ |
| *BPESC1* | -0.188 | -0.291 | -0.081 | 6.782 × 10⁻⁰⁴ | 2.043 × 10⁻⁰² | 2.684 × 10⁻⁰⁴ |
| *IFNA7* | -0.173 | -0.277 | -0.065 | 1.787 × 10⁻⁰³ | 3.500 × 10⁻⁰² | 2.706 × 10⁻⁰⁴ |
| *KRTAP10.6* | -0.177 | -0.280 | -0.069 | 1.412 × 10⁻⁰³ | 3.071 × 10⁻⁰² | 2.989 × 10⁻⁰⁴ |
| *NLRP8* | -0.201 | -0.303 | -0.094 | 2.705 × 10⁻⁰⁴ | 1.433 × 10⁻⁰² | 2.994 × 10⁻⁰⁴ |
| *LOC494141* | -0.208 | -0.310 | -0.101 | 1.670 × 10⁻⁰⁴ | 1.130 × 10⁻⁰² | 3.003 × 10⁻⁰⁴ |
| *LOC400940* | -0.176 | -0.279 | -0.068 | 1.492 × 10⁻⁰³ | 3.140 × 10⁻⁰² | 3.012 × 10⁻⁰⁴ |
| *TAAR9* | -0.194 | -0.296 | -0.087 | 4.545 × 10⁻⁰⁴ | 1.665 × 10⁻⁰² | 3.017 × 10⁻⁰⁴ |
| *DSCR10* | -0.256 | -0.355 | -0.152 | 2.927 × 10⁻⁰⁶ | 1.077 × 10⁻⁰³ | 3.023 × 10⁻⁰⁴ |
| *MMP26* | -0.229 | -0.330 | -0.123 | 3.100 × 10⁻⁰⁵ | 4.439 × 10⁻⁰³ | 3.076 × 10⁻⁰⁴ |
| *USP29* | -0.237 | -0.337 | -0.131 | 1.660 × 10⁻⁰⁵ | 3.170 × 10⁻⁰³ | 3.350 × 10⁻⁰⁴ |
| *CYP4A11* | -0.247 | -0.347 | -0.142 | 6.715 × 10⁻⁰⁶ | 2.006 × 10⁻⁰³ | 3.404 × 10⁻⁰⁴ |
| *OR1E2* | -0.177 | -0.280 | -0.069 | 1.420 × 10⁻⁰³ | 3.071 × 10⁻⁰² | 3.434 × 10⁻⁰⁴ |
| *KCNE1L* | -0.167 | -0.271 | -0.059 | 2.520 × 10⁻⁰³ | 4.338 × 10⁻⁰² | 3.481 × 10⁻⁰⁴ |
| *C8orf71* | -0.239 | -0.339 | -0.133 | 1.398 × 10⁻⁰⁵ | 2.720 × 10⁻⁰³ | 3.663 × 10⁻⁰⁴ |
| *OR1A2* | -0.180 | -0.284 | -0.073 | 1.105 × 10⁻⁰³ | 2.658 × 10⁻⁰² | 3.729 × 10⁻⁰⁴ |
| *KRTAP13.4* | -0.179 | -0.283 | -0.072 | 1.178 × 10⁻⁰³ | 2.759 × 10⁻⁰² | 3.794 × 10⁻⁰⁴ |
| *CRX* | -0.184 | -0.287 | -0.077 | 8.740 × 10⁻⁰⁴ | 2.305 × 10⁻⁰² | 3.801 × 10⁻⁰⁴ |
| *FAM75A5* | -0.306 | -0.402 | -0.204 | 1.844 × 10⁻⁰⁸ | 3.109 × 10⁻⁰⁵ | 3.885 × 10⁻⁰⁴ |
| *FAM47B* | -0.283 | -0.380 | -0.180 | 2.219 × 10⁻⁰⁷ | 2.245 × 10⁻⁰⁴ | 4.483 × 10⁻⁰⁴ |
| *USP26* | -0.268 | -0.366 | -0.164 | 9.926 × 10⁻⁰⁷ | 5.428 × 10⁻⁰⁴ | 4.941 × 10⁻⁰⁴ |
| *ATXN3L* | -0.240 | -0.340 | -0.135 | 1.224 × 10⁻⁰⁵ | 2.527 × 10⁻⁰³ | 5.045 × 10⁻⁰⁴ |
| *RNASE11* | -0.184 | -0.287 | -0.077 | 8.599 × 10⁻⁰⁴ | 2.292 × 10⁻⁰² | 5.286 × 10⁻⁰⁴ |
| *FAM99B* | -0.192 | -0.295 | -0.085 | 5.165 × 10⁻⁰⁴ | 1.768 × 10⁻⁰² | 5.535 × 10⁻⁰⁴ |
| *OR7G2* | -0.176 | -0.280 | -0.069 | 1.448 × 10⁻⁰³ | 3.095 × 10⁻⁰² | 5.872 × 10⁻⁰⁴ |
| *OR6F1* | -0.244 | -0.344 | -0.139 | 9.052 × 10⁻⁰⁶ | 2.348 × 10⁻⁰³ | 5.940 × 10⁻⁰⁴ |
| *OR2A12* | -0.213 | -0.314 | -0.106 | 1.134 × 10⁻⁰⁴ | 9.181 × 10⁻⁰³ | 6.027 × 10⁻⁰⁴ |
| *OR2F2* | -0.186 | -0.289 | -0.078 | 7.846 × 10⁻⁰⁴ | 2.210 × 10⁻⁰² | 6.100 × 10⁻⁰⁴ |
| *KRTAP27.1* | -0.176 | -0.280 | -0.068 | 1.478 × 10⁻⁰³ | 3.128 × 10⁻⁰² | 6.130 × 10⁻⁰⁴ |
| *HTA* | -0.206 | -0.308 | -0.099 | 1.908 × 10⁻⁰⁴ | 1.194 × 10⁻⁰² | 6.424 × 10⁻⁰⁴ |
| *OR6C1* | -0.216 | -0.318 | -0.110 | 8.813 × 10⁻⁰⁵ | 8.106 × 10⁻⁰³ | 6.878 × 10⁻⁰⁴ |
| *SERPINA13* | -0.165 | -0.269 | -0.057 | 2.890 × 10⁻⁰³ | 4.693 × 10⁻⁰² | 7.415 × 10⁻⁰⁴ |
| *LOC339568* | -0.201 | -0.303 | -0.094 | 2.764 × 10⁻⁰⁴ | 1.433 × 10⁻⁰² | 7.595 × 10⁻⁰⁴ |
| *OR13C4* | -0.185 | -0.289 | -0.078 | 7.960 × 10⁻⁰⁴ | 2.224 × 10⁻⁰² | 7.768 × 10⁻⁰⁴ |
| *LOC150527* | -0.231 | -0.332 | -0.125 | 2.658 × 10⁻⁰⁵ | 4.082 × 10⁻⁰³ | 8.307 × 10⁻⁰⁴ |
| *KRTAP10.10* | -0.184 | -0.288 | -0.077 | 8.547 × 10⁻⁰⁴ | 2.288 × 10⁻⁰² | 8.344 × 10⁻⁰⁴ |
| *NPBWR2* | -0.205 | -0.307 | -0.099 | 1.969 × 10⁻⁰⁴ | 1.200 × 10⁻⁰² | 8.782 × 10⁻⁰⁴ |
| *TPD52L3* | -0.228 | -0.329 | -0.122 | 3.351 × 10⁻⁰⁵ | 4.551 × 10⁻⁰³ | 9.132 × 10⁻⁰⁴ |
| *OR4E2* | -0.235 | -0.335 | -0.129 | 2.002 × 10⁻⁰⁵ | 3.462 × 10⁻⁰³ | 9.168 × 10⁻⁰⁴ |
| *OR9Q1* | -0.205 | -0.307 | -0.098 | 2.020 × 10⁻⁰⁴ | 1.220 × 10⁻⁰² | 9.221 × 10⁻⁰⁴ |
| *OR10G2* | -0.190 | -0.293 | -0.083 | 5.904 × 10⁻⁰⁴ | 1.890 × 10⁻⁰² | 9.336 × 10⁻⁰⁴ |
| *OR2W1* | -0.166 | -0.270 | -0.058 | 2.709 × 10⁻⁰³ | 4.541 × 10⁻⁰² | 9.640 × 10⁻⁰⁴ |
| *KRTAP4.9* | -0.295 | -0.391 | -0.192 | 6.393 × 10⁻⁰⁸ | 7.609 × 10⁻⁰⁵ | 9.727 × 10⁻⁰⁴ |
| *USP17* | -0.194 | -0.296 | -0.086 | 4.569 × 10⁻⁰⁴ | 1.666 × 10⁻⁰² | 9.733 × 10⁻⁰⁴ |
| *OR2T12* | -0.180 | -0.283 | -0.072 | 1.156 × 10⁻⁰³ | 2.736 × 10⁻⁰² | 1.023 × 10⁻⁰³ |
| *OR51T1* | -0.217 | -0.318 | -0.111 | 8.198 × 10⁻⁰⁵ | 7.680 × 10⁻⁰³ | 1.028 × 10⁻⁰³ |
| *LOC144776* | -0.208 | -0.310 | -0.102 | 1.608 × 10⁻⁰⁴ | 1.129 × 10⁻⁰² | 1.088 × 10⁻⁰³ |
| *C21orf99* | -0.195 | -0.297 | -0.088 | 4.201 × 10⁻⁰⁴ | 1.592 × 10⁻⁰² | 1.163 × 10⁻⁰³ |
| *LOC255025* | -0.224 | -0.325 | -0.118 | 4.580 × 10⁻⁰⁵ | 5.521 × 10⁻⁰³ | 1.202 × 10⁻⁰³ |
| *ODF3* | -0.179 | -0.283 | -0.072 | 1.184 × 10⁻⁰³ | 2.764 × 10⁻⁰² | 1.277 × 10⁻⁰³ |
| *KRTAP4.12* | -0.192 | -0.295 | -0.085 | 4.945 × 10⁻⁰⁴ | 1.726 × 10⁻⁰² | 1.329 × 10⁻⁰³ |
| *OR2M3* | -0.251 | -0.351 | -0.146 | 4.693 × 10⁻⁰⁶ | 1.557 × 10⁻⁰³ | 1.374 × 10⁻⁰³ |
| *FAM71C* | -0.236 | -0.337 | -0.131 | 1.706 × 10⁻⁰⁵ | 3.199 × 10⁻⁰³ | 1.411 × 10⁻⁰³ |
| *KRTAP2.4* | -0.176 | -0.280 | -0.069 | 1.429 × 10⁻⁰³ | 3.078 × 10⁻⁰² | 1.422 × 10⁻⁰³ |
| *FAM99A* | -0.188 | -0.291 | -0.081 | 6.746 × 10⁻⁰⁴ | 2.042 × 10⁻⁰² | 1.483 × 10⁻⁰³ |
| *OR51G1* | -0.185 | -0.289 | -0.078 | 7.992 × 10⁻⁰⁴ | 2.224 × 10⁻⁰² | 1.485 × 10⁻⁰³ |
| *CARTPT* | -0.183 | -0.286 | -0.076 | 9.342 × 10⁻⁰⁴ | 2.402 × 10⁻⁰² | 1.564 × 10⁻⁰³ |
| *USP17L6P* | -0.203 | -0.305 | -0.096 | 2.395 × 10⁻⁰⁴ | 1.335 × 10⁻⁰² | 1.872 × 10⁻⁰³ |
| *CHRND* | -0.269 | -0.367 | -0.165 | 8.985 × 10⁻⁰⁷ | 5.428 × 10⁻⁰⁴ | 1.883 × 10⁻⁰³ |
| *OR5H14* | -0.182 | -0.286 | -0.075 | 9.716 × 10⁻⁰⁴ | 2.464 × 10⁻⁰² | 1.886 × 10⁻⁰³ |
| *LRIT1* | -0.195 | -0.298 | -0.088 | 4.135 × 10⁻⁰⁴ | 1.573 × 10⁻⁰² | 1.928 × 10⁻⁰³ |
| *GOLGA2P3* | -0.219 | -0.321 | -0.113 | 6.913 × 10⁻⁰⁵ | 7.131 × 10⁻⁰³ | 2.377 × 10⁻⁰³ |
| *OR2C3* | -0.194 | -0.296 | -0.087 | 4.550 × 10⁻⁰⁴ | 1.665 × 10⁻⁰² | 2.456 × 10⁻⁰³ |
| *OR10G4* | -0.210 | -0.312 | -0.104 | 1.351 × 10⁻⁰⁴ | 1.017 × 10⁻⁰² | 2.528 × 10⁻⁰³ |
| *ASXL3* | -0.171 | -0.274 | -0.063 | 2.063 × 10⁻⁰³ | 3.837 × 10⁻⁰² | 2.589 × 10⁻⁰³ |
| *FSCB* | -0.200 | -0.302 | -0.093 | 2.906 × 10⁻⁰⁴ | 1.433 × 10⁻⁰² | 2.630 × 10⁻⁰³ |
| *PRAMEF18* | -0.178 | -0.281 | -0.070 | 1.322 × 10⁻⁰³ | 2.953 × 10⁻⁰² | 2.743 × 10⁻⁰³ |
| *KRTAP9.9* | -0.175 | -0.279 | -0.068 | 1.541 × 10⁻⁰³ | 3.194 × 10⁻⁰² | 2.795 × 10⁻⁰³ |
| *FAM24A* | -0.171 | -0.275 | -0.063 | 2.001 × 10⁻⁰³ | 3.767 × 10⁻⁰² | 2.959 × 10⁻⁰³ |
| *PATE1* | -0.217 | -0.318 | -0.110 | 8.490 × 10⁻⁰⁵ | 7.845 × 10⁻⁰³ | 2.973 × 10⁻⁰³ |
| *LOC157627* | -0.343 | -0.436 | -0.243 | 2.274 × 10⁻¹⁰ | 1.658 × 10⁻⁰⁶ | 3.173 × 10⁻⁰³ |
| *KRTAP4.7* | -0.177 | -0.280 | -0.069 | 1.416 × 10⁻⁰³ | 3.071 × 10⁻⁰² | 3.190 × 10⁻⁰³ |
| *GRID2* | -0.212 | -0.314 | -0.106 | 1.189 × 10⁻⁰⁴ | 9.399 × 10⁻⁰³ | 3.278 × 10⁻⁰³ |
| *OR1N1* | -0.165 | -0.269 | -0.057 | 2.894 × 10⁻⁰³ | 4.693 × 10⁻⁰² | 3.349 × 10⁻⁰³ |
| *POTEB* | -0.207 | -0.309 | -0.100 | 1.737 × 10⁻⁰⁴ | 1.149 × 10⁻⁰² | 3.366 × 10⁻⁰³ |
| *GJC3* | -0.186 | -0.289 | -0.078 | 7.786 × 10⁻⁰⁴ | 2.210 × 10⁻⁰² | 3.457 × 10⁻⁰³ |
| *DKFZP434H168* | -0.229 | -0.330 | -0.123 | 3.155 × 10⁻⁰⁵ | 4.439 × 10⁻⁰³ | 3.477 × 10⁻⁰³ |
| *OR5T3* | -0.226 | -0.327 | -0.120 | 4.161 × 10⁻⁰⁵ | 5.230 × 10⁻⁰³ | 3.649 × 10⁻⁰³ |
| *GPX5* | -0.180 | -0.284 | -0.073 | 1.107 × 10⁻⁰³ | 2.658 × 10⁻⁰² | 3.918 × 10⁻⁰³ |
| *OR51G2* | -0.182 | -0.285 | -0.074 | 1.029 × 10⁻⁰³ | 2.555 × 10⁻⁰² | 4.000 × 10⁻⁰³ |
| *GLRA1* | -0.176 | -0.279 | -0.068 | 1.489 × 10⁻⁰³ | 3.138 × 10⁻⁰² | 4.092 × 10⁻⁰³ |
| *SSX7* | -0.178 | -0.281 | -0.070 | 1.313 × 10⁻⁰³ | 2.947 × 10⁻⁰² | 4.141 × 10⁻⁰³ |
| *OR9A2* | -0.201 | -0.303 | -0.094 | 2.800 × 10⁻⁰⁴ | 1.433 × 10⁻⁰² | 4.151 × 10⁻⁰³ |
| *C3orf27* | -0.189 | -0.292 | -0.082 | 6.250 × 10⁻⁰⁴ | 1.937 × 10⁻⁰² | 4.276 × 10⁻⁰³ |
| *OR5T1* | -0.179 | -0.282 | -0.071 | 1.248 × 10⁻⁰³ | 2.856 × 10⁻⁰² | 4.398 × 10⁻⁰³ |
| *OR8K5* | -0.192 | -0.295 | -0.085 | 5.202 × 10⁻⁰⁴ | 1.768 × 10⁻⁰² | 4.436 × 10⁻⁰³ |
| *CCDC70* | -0.168 | -0.272 | -0.060 | 2.479 × 10⁻⁰³ | 4.289 × 10⁻⁰² | 4.719 × 10⁻⁰³ |
| *WDR66* | 0.194 | 0.087 | 0.297 | 4.343 × 10⁻⁰⁴ | 1.630 × 10⁻⁰² | 4.874 × 10⁻⁰³ |
| *GPR148* | -0.215 | -0.316 | -0.108 | 9.939 × 10⁻⁰⁵ | 8.668 × 10⁻⁰³ | 5.035 × 10⁻⁰³ |
| *PRSS33* | -0.166 | -0.270 | -0.058 | 2.795 × 10⁻⁰³ | 4.607 × 10⁻⁰² | 5.367 × 10⁻⁰³ |
| *OR8K3* | -0.181 | -0.284 | -0.073 | 1.086 × 10⁻⁰³ | 2.628 × 10⁻⁰² | 5.554 × 10⁻⁰³ |
| *TTTY7* | -0.185 | -0.288 | -0.077 | 8.366 × 10⁻⁰⁴ | 2.272 × 10⁻⁰² | 5.703 × 10⁻⁰³ |
| *TRYX3* | -0.178 | -0.281 | -0.070 | 1.315 × 10⁻⁰³ | 2.947 × 10⁻⁰² | 5.739 × 10⁻⁰³ |
| *GFRAL* | -0.209 | -0.311 | -0.103 | 1.465 × 10⁻⁰⁴ | 1.055 × 10⁻⁰² | 5.743 × 10⁻⁰³ |
| *NKX6.3* | -0.166 | -0.270 | -0.058 | 2.731 × 10⁻⁰³ | 4.561 × 10⁻⁰² | 5.861 × 10⁻⁰³ |
| *CATSPER4* | -0.166 | -0.270 | -0.058 | 2.753 × 10⁻⁰³ | 4.579 × 10⁻⁰² | 6.574 × 10⁻⁰³ |
| *IFNA2* | -0.181 | -0.285 | -0.074 | 1.049 × 10⁻⁰³ | 2.570 × 10⁻⁰² | 6.778 × 10⁻⁰³ |
| *CNGA2* | -0.181 | -0.285 | -0.074 | 1.051 × 10⁻⁰³ | 2.570 × 10⁻⁰² | 7.183 × 10⁻⁰³ |
| *OR1C1* | -0.242 | -0.342 | -0.136 | 1.086 × 10⁻⁰⁵ | 2.466 × 10⁻⁰³ | 7.479 × 10⁻⁰³ |
| *LRP10* | 0.165 | 0.057 | 0.269 | 2.872 × 10⁻⁰³ | 4.686 × 10⁻⁰² | 7.631 × 10⁻⁰³ |
| *CLRN2* | -0.172 | -0.276 | -0.064 | 1.876 × 10⁻⁰³ | 3.598 × 10⁻⁰² | 8.429 × 10⁻⁰³ |
| *PRR23A* | -0.236 | -0.337 | -0.131 | 1.723 × 10⁻⁰⁵ | 3.199 × 10⁻⁰³ | 8.453 × 10⁻⁰³ |
| *OR4A15* | -0.186 | -0.289 | -0.078 | 7.839 × 10⁻⁰⁴ | 2.210 × 10⁻⁰² | 8.960 × 10⁻⁰³ |
| *GDEP* | -0.207 | -0.309 | -0.101 | 1.694 × 10⁻⁰⁴ | 1.140 × 10⁻⁰² | 9.132 × 10⁻⁰³ |
| *NSUN4* | -0.186 | -0.289 | -0.079 | 7.577 × 10⁻⁰⁴ | 2.184 × 10⁻⁰² | 9.179 × 10⁻⁰³ |
| *APOL5* | -0.166 | -0.270 | -0.058 | 2.701 × 10⁻⁰³ | 4.536 × 10⁻⁰² | 9.180 × 10⁻⁰³ |
| *FLJ44082* | -0.191 | -0.294 | -0.084 | 5.339 × 10⁻⁰⁴ | 1.783 × 10⁻⁰² | 9.195 × 10⁻⁰³ |
| *IFNA4* | -0.177 | -0.280 | -0.069 | 1.391 × 10⁻⁰³ | 3.055 × 10⁻⁰² | 9.708 × 10⁻⁰³ |
| *LRRC30* | -0.254 | -0.353 | -0.149 | 3.582 × 10⁻⁰⁶ | 1.228 × 10⁻⁰³ | 9.994 × 10⁻⁰³ |
| *H1FOO* | -0.164 | -0.268 | -0.056 | 3.139 × 10⁻⁰³ | 4.935 × 10⁻⁰² | 1.002 × 10⁻⁰² |
| *OR7G3* | -0.199 | -0.301 | -0.092 | 3.155 × 10⁻⁰⁴ | 1.433 × 10⁻⁰² | 1.028 × 10⁻⁰² |
| *REG1B* | -0.193 | -0.295 | -0.085 | 4.922 × 10⁻⁰⁴ | 1.726 × 10⁻⁰² | 1.050 × 10⁻⁰² |
| *OR4F5* | -0.221 | -0.322 | -0.115 | 5.896 × 10⁻⁰⁵ | 6.480 × 10⁻⁰³ | 1.068 × 10⁻⁰² |
| *OR2B2* | -0.201 | -0.303 | -0.094 | 2.722 × 10⁻⁰⁴ | 1.433 × 10⁻⁰² | 1.078 × 10⁻⁰² |
| *OR9G9* | -0.173 | -0.277 | -0.066 | 1.735 × 10⁻⁰³ | 3.448 × 10⁻⁰² | 1.086 × 10⁻⁰² |
| *OR6N1* | -0.206 | -0.308 | -0.099 | 1.902 × 10⁻⁰⁴ | 1.194 × 10⁻⁰² | 1.104 × 10⁻⁰² |
| *NAALAD2* | -0.171 | -0.275 | -0.063 | 1.989 × 10⁻⁰³ | 3.754 × 10⁻⁰² | 1.202 × 10⁻⁰² |
| *MS4A10* | -0.277 | -0.375 | -0.173 | 4.117 × 10⁻⁰⁷ | 3.332 × 10⁻⁰⁴ | 1.225 × 10⁻⁰² |
| *PRSS37* | -0.173 | -0.276 | -0.065 | 1.818 × 10⁻⁰³ | 3.536 × 10⁻⁰² | 1.234 × 10⁻⁰² |
| *LOC286135* | -0.187 | -0.290 | -0.079 | 7.312 × 10⁻⁰⁴ | 2.129 × 10⁻⁰² | 1.264 × 10⁻⁰² |
| *LOC644669* | -0.243 | -0.343 | -0.138 | 9.502 × 10⁻⁰⁶ | 2.409 × 10⁻⁰³ | 1.278 × 10⁻⁰² |
| *C9orf131* | -0.181 | -0.285 | -0.074 | 1.039 × 10⁻⁰³ | 2.562 × 10⁻⁰² | 1.290 × 10⁻⁰² |
| *KRTAP4.4* | -0.199 | -0.301 | -0.092 | 3.129 × 10⁻⁰⁴ | 1.433 × 10⁻⁰² | 1.314 × 10⁻⁰² |
| *KRTAP12.2* | -0.173 | -0.276 | -0.065 | 1.810 × 10⁻⁰³ | 3.525 × 10⁻⁰² | 1.441 × 10⁻⁰² |
| *IQCF2* | -0.186 | -0.289 | -0.079 | 7.732 × 10⁻⁰⁴ | 2.207 × 10⁻⁰² | 1.504 × 10⁻⁰² |
| *PRAMEF22* | -0.207 | -0.309 | -0.100 | 1.800 × 10⁻⁰⁴ | 1.167 × 10⁻⁰² | 1.517 × 10⁻⁰² |
| *TTTY18* | -0.229 | -0.329 | -0.123 | 3.291 × 10⁻⁰⁵ | 4.541 × 10⁻⁰³ | 1.643 × 10⁻⁰² |
| *EIF4E1B* | -0.167 | -0.271 | -0.059 | 2.617 × 10⁻⁰³ | 4.441 × 10⁻⁰² | 1.652 × 10⁻⁰² |
| *OR10C1* | -0.200 | -0.302 | -0.093 | 3.008 × 10⁻⁰⁴ | 1.433 × 10⁻⁰² | 1.741 × 10⁻⁰² |
| *C17orf102* | -0.340 | -0.433 | -0.240 | 3.278 × 10⁻¹⁰ | 1.658 × 10⁻⁰⁶ | 1.751 × 10⁻⁰² |
| *OR6C3* | -0.176 | -0.279 | -0.068 | 1.498 × 10⁻⁰³ | 3.145 × 10⁻⁰² | 1.754 × 10⁻⁰² |
| *OLIG2* | -0.181 | -0.284 | -0.074 | 1.062 × 10⁻⁰³ | 2.588 × 10⁻⁰² | 1.792 × 10⁻⁰² |
| *OR51A4* | -0.177 | -0.281 | -0.069 | 1.375 × 10⁻⁰³ | 3.038 × 10⁻⁰² | 1.797 × 10⁻⁰² |
| *OR7A17* | -0.194 | -0.297 | -0.087 | 4.446 × 10⁻⁰⁴ | 1.654 × 10⁻⁰² | 1.823 × 10⁻⁰² |
| *MMD2* | -0.189 | -0.292 | -0.082 | 6.206 × 10⁻⁰⁴ | 1.930 × 10⁻⁰² | 2.000 × 10⁻⁰² |
| *LOC144742* | -0.205 | -0.307 | -0.098 | 2.079 × 10⁻⁰⁴ | 1.241 × 10⁻⁰² | 2.091 × 10⁻⁰² |
| *SNORD116.4* | -0.170 | -0.274 | -0.062 | 2.125 × 10⁻⁰³ | 3.898 × 10⁻⁰² | 2.104 × 10⁻⁰² |
| *GAGE1* | -0.176 | -0.279 | -0.068 | 1.516 × 10⁻⁰³ | 3.168 × 10⁻⁰² | 2.115 × 10⁻⁰² |
| *DUXA* | -0.234 | -0.335 | -0.129 | 2.033 × 10⁻⁰⁵ | 3.486 × 10⁻⁰³ | 2.127 × 10⁻⁰² |
| *ZNF681* | -0.167 | -0.271 | -0.059 | 2.520 × 10⁻⁰³ | 4.338 × 10⁻⁰² | 2.160 × 10⁻⁰² |
| *OR2AT4* | -0.166 | -0.270 | -0.058 | 2.755 × 10⁻⁰³ | 4.579 × 10⁻⁰² | 2.197 × 10⁻⁰² |
| *GUCA2B* | -0.211 | -0.312 | -0.104 | 1.350 × 10⁻⁰⁴ | 1.017 × 10⁻⁰² | 2.221 × 10⁻⁰² |
| *SEMA4G* | -0.178 | -0.282 | -0.071 | 1.267 × 10⁻⁰³ | 2.875 × 10⁻⁰² | 2.257 × 10⁻⁰² |
| *C1orf105* | -0.173 | -0.277 | -0.065 | 1.765 × 10⁻⁰³ | 3.467 × 10⁻⁰² | 2.294 × 10⁻⁰² |
| *PRLHR* | -0.166 | -0.270 | -0.058 | 2.685 × 10⁻⁰³ | 4.518 × 10⁻⁰² | 2.325 × 10⁻⁰² |
| *TMEM174* | -0.242 | -0.342 | -0.136 | 1.097 × 10⁻⁰⁵ | 2.466 × 10⁻⁰³ | 2.398 × 10⁻⁰² |
| *OR4A16* | -0.186 | -0.289 | -0.078 | 7.852 × 10⁻⁰⁴ | 2.210 × 10⁻⁰² | 2.430 × 10⁻⁰² |
| *CHRNE* | -0.225 | -0.326 | -0.118 | 4.534 × 10⁻⁰⁵ | 5.521 × 10⁻⁰³ | 2.536 × 10⁻⁰² |
| *GABRR3* | -0.177 | -0.281 | -0.070 | 1.366 × 10⁻⁰³ | 3.031 × 10⁻⁰² | 2.539 × 10⁻⁰² |
| *NOD2* | 0.200 | 0.093 | 0.303 | 2.882 × 10⁻⁰⁴ | 1.433 × 10⁻⁰² | 2.540 × 10⁻⁰² |
| *FCER1A* | 0.165 | 0.057 | 0.269 | 2.884 × 10⁻⁰³ | 4.693 × 10⁻⁰² | 2.577 × 10⁻⁰² |
| *IMMP1L* | -0.170 | -0.274 | -0.062 | 2.164 × 10⁻⁰³ | 3.945 × 10⁻⁰² | 2.583 × 10⁻⁰² |
| *PGLYRP1* | -0.168 | -0.272 | -0.060 | 2.454 × 10⁻⁰³ | 4.281 × 10⁻⁰² | 2.799 × 10⁻⁰² |
| *LOC285692* | -0.163 | -0.268 | -0.055 | 3.183 × 10⁻⁰³ | 4.982 × 10⁻⁰² | 2.916 × 10⁻⁰² |
| *BRE* | -0.174 | -0.278 | -0.066 | 1.689 × 10⁻⁰³ | 3.386 × 10⁻⁰² | 2.925 × 10⁻⁰² |
| *NEUROD4* | -0.180 | -0.283 | -0.072 | 1.174 × 10⁻⁰³ | 2.756 × 10⁻⁰² | 2.984 × 10⁻⁰² |
| *C21orf71* | -0.167 | -0.271 | -0.059 | 2.548 × 10⁻⁰³ | 4.363 × 10⁻⁰² | 3.031 × 10⁻⁰² |
| *OR10A4* | -0.206 | -0.308 | -0.099 | 1.953 × 10⁻⁰⁴ | 1.200 × 10⁻⁰² | 3.070 × 10⁻⁰² |
| *OR1L1* | -0.199 | -0.301 | -0.092 | 3.175 × 10⁻⁰⁴ | 1.433 × 10⁻⁰² | 3.081 × 10⁻⁰² |
| *KCNK18* | -0.226 | -0.327 | -0.120 | 3.962 × 10⁻⁰⁵ | 5.106 × 10⁻⁰³ | 3.090 × 10⁻⁰² |
| *GLYCAM1* | -0.187 | -0.290 | -0.080 | 6.951 × 10⁻⁰⁴ | 2.069 × 10⁻⁰² | 3.117 × 10⁻⁰² |
| *F2RL2* | 0.167 | 0.059 | 0.271 | 2.559 × 10⁻⁰³ | 4.374 × 10⁻⁰² | 3.175 × 10⁻⁰² |
| *MYH7* | -0.198 | -0.301 | -0.091 | 3.287 × 10⁻⁰⁴ | 1.433 × 10⁻⁰² | 3.281 × 10⁻⁰² |
| *ISM2* | -0.169 | -0.273 | -0.061 | 2.262 × 10⁻⁰³ | 4.079 × 10⁻⁰² | 3.304 × 10⁻⁰² |
| *OR2AG1* | -0.218 | -0.319 | -0.112 | 7.572 × 10⁻⁰⁵ | 7.392 × 10⁻⁰³ | 3.333 × 10⁻⁰² |
| *OR5M1* | -0.190 | -0.293 | -0.083 | 5.808 × 10⁻⁰⁴ | 1.868 × 10⁻⁰² | 3.365 × 10⁻⁰² |
| *C14orf70* | -0.210 | -0.311 | -0.103 | 1.439 × 10⁻⁰⁴ | 1.044 × 10⁻⁰² | 3.426 × 10⁻⁰² |
| *PLA2G4E* | 0.194 | 0.087 | 0.297 | 4.399 × 10⁻⁰⁴ | 1.641 × 10⁻⁰² | 3.498 × 10⁻⁰² |
| *HAS3* | 0.165 | 0.057 | 0.269 | 2.894 × 10⁻⁰³ | 4.693 × 10⁻⁰² | 3.529 × 10⁻⁰² |
| *MORC3* | 0.188 | 0.081 | 0.291 | 6.546 × 10⁻⁰⁴ | 1.992 × 10⁻⁰² | 3.545 × 10⁻⁰² |
| *SLC32A1* | -0.230 | -0.331 | -0.124 | 2.870 × 10⁻⁰⁵ | 4.262 × 10⁻⁰³ | 3.619 × 10⁻⁰² |
| *FLOT1* | -0.171 | -0.274 | -0.063 | 2.069 × 10⁻⁰³ | 3.841 × 10⁻⁰² | 3.653 × 10⁻⁰² |
| *FAM75A3* | -0.260 | -0.359 | -0.156 | 2.043 × 10⁻⁰⁶ | 8.792 × 10⁻⁰⁴ | 3.755 × 10⁻⁰² |
| *DKFZp434L192* | -0.212 | -0.314 | -0.106 | 1.196 × 10⁻⁰⁴ | 9.418 × 10⁻⁰³ | 3.886 × 10⁻⁰² |
| *LECT2* | -0.180 | -0.283 | -0.072 | 1.152 × 10⁻⁰³ | 2.736 × 10⁻⁰² | 3.887 × 10⁻⁰² |
| *GYPA* | -0.272 | -0.370 | -0.168 | 6.749 × 10⁻⁰⁷ | 4.878 × 10⁻⁰⁴ | 3.927 × 10⁻⁰² |
| *LELP1* | -0.227 | -0.328 | -0.121 | 3.659 × 10⁻⁰⁵ | 4.840 × 10⁻⁰³ | 3.949 × 10⁻⁰² |
| *STAT3* | -0.164 | -0.268 | -0.056 | 3.125 × 10⁻⁰³ | 4.924 × 10⁻⁰² | 3.954 × 10⁻⁰² |
| *DPF2* | -0.192 | -0.294 | -0.084 | 5.230 × 10⁻⁰⁴ | 1.768 × 10⁻⁰² | 4.109 × 10⁻⁰² |
| *OR1N2* | -0.171 | -0.275 | -0.063 | 1.997 × 10⁻⁰³ | 3.762 × 10⁻⁰² | 4.152 × 10⁻⁰² |
| *ADAM30* | -0.187 | -0.290 | -0.079 | 7.252 × 10⁻⁰⁴ | 2.122 × 10⁻⁰² | 4.203 × 10⁻⁰² |
| *SLC26A8* | -0.182 | -0.285 | -0.074 | 1.017 × 10⁻⁰³ | 2.531 × 10⁻⁰² | 4.205 × 10⁻⁰² |
| *TAAR6* | -0.171 | -0.274 | -0.063 | 2.064 × 10⁻⁰³ | 3.837 × 10⁻⁰² | 4.355 × 10⁻⁰² |
| *FAM161A* | -0.177 | -0.280 | -0.069 | 1.406 × 10⁻⁰³ | 3.065 × 10⁻⁰² | 4.400 × 10⁻⁰² |
| *OR13C8* | -0.174 | -0.278 | -0.066 | 1.652 × 10⁻⁰³ | 3.340 × 10⁻⁰² | 4.431 × 10⁻⁰² |
| *OR7E156P* | -0.296 | -0.392 | -0.193 | 5.744 × 10⁻⁰⁸ | 7.265 × 10⁻⁰⁵ | 4.431 × 10⁻⁰² |
| *OR2M4* | -0.195 | -0.298 | -0.088 | 4.066 × 10⁻⁰⁴ | 1.561 × 10⁻⁰² | 4.469 × 10⁻⁰² |
| *LOC340357* | -0.190 | -0.293 | -0.082 | 6.016 × 10⁻⁰⁴ | 1.911 × 10⁻⁰² | 4.534 × 10⁻⁰² |
| *RAC2* | 0.181 | 0.073 | 0.284 | 1.091 × 10⁻⁰³ | 2.631 × 10⁻⁰² | 4.606 × 10⁻⁰² |
| *LRP5* | -0.168 | -0.272 | -0.061 | 2.359 × 10⁻⁰³ | 4.195 × 10⁻⁰² | 4.677 × 10⁻⁰² |
| *PCIF1* | -0.239 | -0.339 | -0.134 | 1.353 × 10⁻⁰⁵ | 2.685 × 10⁻⁰³ | 4.761 × 10⁻⁰² |
| *SLC6A19* | -0.179 | -0.282 | -0.071 | 1.211 × 10⁻⁰³ | 2.804 × 10⁻⁰² | 4.807 × 10⁻⁰² |
| *AKAP4* | -0.183 | -0.286 | -0.076 | 9.218 × 10⁻⁰⁴ | 2.384 × 10⁻⁰² | 4.863 × 10⁻⁰² |
| *USP27X* | -0.171 | -0.274 | -0.063 | 2.065 × 10⁻⁰³ | 3.837 × 10⁻⁰² | 4.979 × 10⁻⁰² |
| *ZW10* | -0.179 | -0.282 | -0.071 | 1.226 × 10⁻⁰³ | 2.820 × 10⁻⁰² | 5.097 × 10⁻⁰² |
| *GALNT9* | -0.169 | -0.273 | -0.061 | 2.307 × 10⁻⁰³ | 4.125 × 10⁻⁰² | 5.106 × 10⁻⁰² |
| *RBMXL3* | -0.224 | -0.325 | -0.117 | 4.926 × 10⁻⁰⁵ | 5.829 × 10⁻⁰³ | 5.160 × 10⁻⁰² |
| *CALCA* | -0.168 | -0.272 | -0.060 | 2.444 × 10⁻⁰³ | 4.273 × 10⁻⁰² | 5.212 × 10⁻⁰² |
| *IFNW1* | -0.164 | -0.268 | -0.056 | 3.139 × 10⁻⁰³ | 4.935 × 10⁻⁰² | 5.254 × 10⁻⁰² |
| *PATE2* | -0.184 | -0.287 | -0.076 | 8.834 × 10⁻⁰⁴ | 2.312 × 10⁻⁰² | 5.346 × 10⁻⁰² |
| *HNRNPU* | -0.182 | -0.285 | -0.075 | 9.868 × 10⁻⁰⁴ | 2.493 × 10⁻⁰² | 5.375 × 10⁻⁰² |
| *HPCAL4* | -0.192 | -0.295 | -0.085 | 5.054 × 10⁻⁰⁴ | 1.751 × 10⁻⁰² | 5.409 × 10⁻⁰² |
| *SLC17A2* | -0.241 | -0.341 | -0.135 | 1.188 × 10⁻⁰⁵ | 2.478 × 10⁻⁰³ | 5.424 × 10⁻⁰² |
| *PAR5* | -0.202 | -0.304 | -0.095 | 2.585 × 10⁻⁰⁴ | 1.410 × 10⁻⁰² | 5.434 × 10⁻⁰² |
| *RBMX* | -0.203 | -0.305 | -0.096 | 2.345 × 10⁻⁰⁴ | 1.324 × 10⁻⁰² | 5.448 × 10⁻⁰² |
| *NAT10* | -0.181 | -0.285 | -0.074 | 1.048 × 10⁻⁰³ | 2.570 × 10⁻⁰² | 5.474 × 10⁻⁰² |
| *LOC284798* | -0.213 | -0.315 | -0.106 | 1.129 × 10⁻⁰⁴ | 9.178 × 10⁻⁰³ | 5.625 × 10⁻⁰² |
| *SPSB1* | 0.173 | 0.065 | 0.277 | 1.797 × 10⁻⁰³ | 3.511 × 10⁻⁰² | 5.720 × 10⁻⁰² |
| *CD28* | 0.166 | 0.058 | 0.270 | 2.741 × 10⁻⁰³ | 4.573 × 10⁻⁰² | 5.903 × 10⁻⁰² |
| *OR1D4* | -0.208 | -0.310 | -0.101 | 1.661 × 10⁻⁰⁴ | 1.130 × 10⁻⁰² | 5.914 × 10⁻⁰² |
| *APLNR* | 0.174 | 0.066 | 0.277 | 1.714 × 10⁻⁰³ | 3.421 × 10⁻⁰² | 6.009 × 10⁻⁰² |
| *CYLC2* | -0.265 | -0.363 | -0.161 | 1.312 × 10⁻⁰⁶ | 6.852 × 10⁻⁰⁴ | 6.072 × 10⁻⁰² |
| *ZCCHC16* | -0.204 | -0.306 | -0.097 | 2.227 × 10⁻⁰⁴ | 1.303 × 10⁻⁰² | 6.092 × 10⁻⁰² |
| *RIN2* | 0.175 | 0.067 | 0.278 | 1.594 × 10⁻⁰³ | 3.262 × 10⁻⁰² | 6.181 × 10⁻⁰² |
| *C6orf146* | -0.218 | -0.319 | -0.111 | 7.846 × 10⁻⁰⁵ | 7.453 × 10⁻⁰³ | 6.223 × 10⁻⁰² |
| *DEFB125* | -0.202 | -0.305 | -0.095 | 2.465 × 10⁻⁰⁴ | 1.363 × 10⁻⁰² | 6.241 × 10⁻⁰² |
| *C17orf88* | -0.165 | -0.269 | -0.057 | 2.965 × 10⁻⁰³ | 4.780 × 10⁻⁰² | 6.276 × 10⁻⁰² |
| *DSCR4* | -0.214 | -0.316 | -0.107 | 1.040 × 10⁻⁰⁴ | 8.766 × 10⁻⁰³ | 6.351 × 10⁻⁰² |
| *NAV1* | 0.176 | 0.068 | 0.279 | 1.486 × 10⁻⁰³ | 3.135 × 10⁻⁰² | 6.378 × 10⁻⁰² |
| *FAM69A* | 0.218 | 0.112 | 0.319 | 7.548 × 10⁻⁰⁵ | 7.392 × 10⁻⁰³ | 6.429 × 10⁻⁰² |
| *GRIA4* | -0.234 | -0.334 | -0.128 | 2.175 × 10⁻⁰⁵ | 3.514 × 10⁻⁰³ | 6.469 × 10⁻⁰² |
| *CXXC4* | -0.189 | -0.292 | -0.082 | 6.180 × 10⁻⁰⁴ | 1.927 × 10⁻⁰² | 6.485 × 10⁻⁰² |
| *LOC284232* | -0.178 | -0.281 | -0.070 | 1.306 × 10⁻⁰³ | 2.943 × 10⁻⁰² | 6.572 × 10⁻⁰² |
| *STMN1* | -0.178 | -0.282 | -0.070 | 1.289 × 10⁻⁰³ | 2.914 × 10⁻⁰² | 6.638 × 10⁻⁰² |
| *TMEM108* | -0.170 | -0.274 | -0.062 | 2.122 × 10⁻⁰³ | 3.898 × 10⁻⁰² | 6.815 × 10⁻⁰² |
| *FAM75A2* | -0.217 | -0.319 | -0.111 | 8.162 × 10⁻⁰⁵ | 7.680 × 10⁻⁰³ | 6.822 × 10⁻⁰² |
| *MYF5* | -0.196 | -0.299 | -0.089 | 3.815 × 10⁻⁰⁴ | 1.517 × 10⁻⁰² | 6.822 × 10⁻⁰² |
| *MAGEB10* | -0.173 | -0.277 | -0.065 | 1.753 × 10⁻⁰³ | 3.459 × 10⁻⁰² | 6.908 × 10⁻⁰² |
| *OR6C68* | -0.193 | -0.296 | -0.086 | 4.716 × 10⁻⁰⁴ | 1.690 × 10⁻⁰² | 7.310 × 10⁻⁰² |
| *LOC723972* | -0.192 | -0.295 | -0.085 | 5.187 × 10⁻⁰⁴ | 1.768 × 10⁻⁰² | 7.416 × 10⁻⁰² |
| *DSCR8* | -0.206 | -0.308 | -0.099 | 1.914 × 10⁻⁰⁴ | 1.194 × 10⁻⁰² | 7.423 × 10⁻⁰² |
| *POLA1* | -0.189 | -0.292 | -0.081 | 6.442 × 10⁻⁰⁴ | 1.979 × 10⁻⁰² | 7.493 × 10⁻⁰² |
| *ZNF84* | -0.198 | -0.300 | -0.091 | 3.361 × 10⁻⁰⁴ | 1.433 × 10⁻⁰² | 7.703 × 10⁻⁰² |
| *RPAIN* | -0.183 | -0.286 | -0.075 | 9.613 × 10⁻⁰⁴ | 2.447 × 10⁻⁰² | 7.726 × 10⁻⁰² |
| *GAS2* | -0.193 | -0.296 | -0.086 | 4.758 × 10⁻⁰⁴ | 1.691 × 10⁻⁰² | 7.739 × 10⁻⁰² |
| *SMARCD1* | -0.179 | -0.282 | -0.071 | 1.242 × 10⁻⁰³ | 2.849 × 10⁻⁰² | 7.803 × 10⁻⁰² |
| *C1orf54* | 0.169 | 0.061 | 0.273 | 2.310 × 10⁻⁰³ | 4.125 × 10⁻⁰² | 7.935 × 10⁻⁰² |
| *STEAP3* | 0.185 | 0.078 | 0.288 | 8.006 × 10⁻⁰⁴ | 2.225 × 10⁻⁰² | 7.994 × 10⁻⁰² |
| *HMGB4* | -0.208 | -0.310 | -0.101 | 1.644 × 10⁻⁰⁴ | 1.130 × 10⁻⁰² | 8.295 × 10⁻⁰² |
| *TBCD* | -0.175 | -0.279 | -0.068 | 1.530 × 10⁻⁰³ | 3.178 × 10⁻⁰² | 8.342 × 10⁻⁰² |
| *PRR5L* | 0.164 | 0.056 | 0.268 | 3.045 × 10⁻⁰³ | 4.836 × 10⁻⁰² | 8.373 × 10⁻⁰² |
| *GPR151* | -0.165 | -0.269 | -0.057 | 2.946 × 10⁻⁰³ | 4.758 × 10⁻⁰² | 8.418 × 10⁻⁰² |
| *LRP4* | -0.164 | -0.268 | -0.056 | 3.034 × 10⁻⁰³ | 4.830 × 10⁻⁰² | 8.538 × 10⁻⁰² |
| *ZKSCAN3* | -0.185 | -0.288 | -0.077 | 8.473 × 10⁻⁰⁴ | 2.280 × 10⁻⁰² | 8.850 × 10⁻⁰² |
| *OR5H6* | -0.184 | -0.287 | -0.077 | 8.589 × 10⁻⁰⁴ | 2.292 × 10⁻⁰² | 8.999 × 10⁻⁰² |
| *LOC643955* | -0.182 | -0.285 | -0.075 | 9.927 × 10⁻⁰⁴ | 2.499 × 10⁻⁰² | 9.017 × 10⁻⁰² |
| *ZNF879* | -0.187 | -0.290 | -0.080 | 7.022 × 10⁻⁰⁴ | 2.078 × 10⁻⁰² | 9.022 × 10⁻⁰² |
| *DDX1* | -0.172 | -0.276 | -0.064 | 1.921 × 10⁻⁰³ | 3.660 × 10⁻⁰² | 9.027 × 10⁻⁰² |
| *ZIM3* | -0.268 | -0.366 | -0.164 | 9.700 × 10⁻⁰⁷ | 5.428 × 10⁻⁰⁴ | 9.076 × 10⁻⁰² |
| *MT1G* | -0.164 | -0.268 | -0.056 | 3.063 × 10⁻⁰³ | 4.853 × 10⁻⁰² | 9.143 × 10⁻⁰² |
| *RWDD2B* | 0.188 | 0.080 | 0.291 | 6.909 × 10⁻⁰⁴ | 2.069 × 10⁻⁰² | 9.293 × 10⁻⁰² |
| *ZIC3* | -0.175 | -0.279 | -0.067 | 1.575 × 10⁻⁰³ | 3.233 × 10⁻⁰² | 9.464 × 10⁻⁰² |
| *FTSJ3* | -0.190 | -0.292 | -0.082 | 6.034 × 10⁻⁰⁴ | 1.911 × 10⁻⁰² | 9.478 × 10⁻⁰² |
| *CCL22* | 0.180 | 0.072 | 0.283 | 1.152 × 10⁻⁰³ | 2.736 × 10⁻⁰² | 9.673 × 10⁻⁰² |
| *PIWIL3* | -0.180 | -0.283 | -0.072 | 1.141 × 10⁻⁰³ | 2.721 × 10⁻⁰² | 9.718 × 10⁻⁰² |
| *RGS20* | 0.175 | 0.068 | 0.279 | 1.526 × 10⁻⁰³ | 3.177 × 10⁻⁰² | 9.843 × 10⁻⁰² |
| *ZC3H10* | -0.201 | -0.303 | -0.094 | 2.743 × 10⁻⁰⁴ | 1.433 × 10⁻⁰² | 9.923 × 10⁻⁰² |
| *PSPC1* | -0.219 | -0.320 | -0.113 | 7.013 × 10⁻⁰⁵ | 7.131 × 10⁻⁰³ | 1.006 × 10⁻⁰¹ |
| *LOC100190940* | -0.181 | -0.284 | -0.073 | 1.067 × 10⁻⁰³ | 2.598 × 10⁻⁰² | 1.006 × 10⁻⁰¹ |
| *PAX2* | -0.177 | -0.280 | -0.069 | 1.396 × 10⁻⁰³ | 3.055 × 10⁻⁰² | 1.025 × 10⁻⁰¹ |
| *DDI1* | -0.176 | -0.280 | -0.069 | 1.432 × 10⁻⁰³ | 3.078 × 10⁻⁰² | 1.028 × 10⁻⁰¹ |
| *VN1R4* | -0.198 | -0.300 | -0.091 | 3.439 × 10⁻⁰⁴ | 1.433 × 10⁻⁰² | 1.028 × 10⁻⁰¹ |
| *USP17L2* | -0.171 | -0.275 | -0.064 | 1.949 × 10⁻⁰³ | 3.702 × 10⁻⁰² | 1.033 × 10⁻⁰¹ |
| *FOXG1* | -0.167 | -0.271 | -0.059 | 2.560 × 10⁻⁰³ | 4.374 × 10⁻⁰² | 1.037 × 10⁻⁰¹ |
| *SLC17A1* | -0.184 | -0.287 | -0.077 | 8.578 × 10⁻⁰⁴ | 2.292 × 10⁻⁰² | 1.042 × 10⁻⁰¹ |
| *LMOD2* | -0.173 | -0.277 | -0.065 | 1.765 × 10⁻⁰³ | 3.467 × 10⁻⁰² | 1.074 × 10⁻⁰¹ |
| *C15orf2* | -0.208 | -0.310 | -0.101 | 1.625 × 10⁻⁰⁴ | 1.130 × 10⁻⁰² | 1.083 × 10⁻⁰¹ |
| *SQRDL* | 0.169 | 0.061 | 0.273 | 2.229 × 10⁻⁰³ | 4.034 × 10⁻⁰² | 1.113 × 10⁻⁰¹ |
| *C22orf25* | 0.166 | 0.058 | 0.270 | 2.697 × 10⁻⁰³ | 4.532 × 10⁻⁰² | 1.117 × 10⁻⁰¹ |
| *HNRNPL* | -0.164 | -0.269 | -0.056 | 3.006 × 10⁻⁰³ | 4.813 × 10⁻⁰² | 1.119 × 10⁻⁰¹ |
| *SHOX* | -0.185 | -0.288 | -0.077 | 8.416 × 10⁻⁰⁴ | 2.274 × 10⁻⁰² | 1.122 × 10⁻⁰¹ |
| *LASS1* | -0.178 | -0.282 | -0.070 | 1.290 × 10⁻⁰³ | 2.914 × 10⁻⁰² | 1.126 × 10⁻⁰¹ |
| *DLGAP1* | -0.177 | -0.280 | -0.069 | 1.403 × 10⁻⁰³ | 3.063 × 10⁻⁰² | 1.130 × 10⁻⁰¹ |
| *HIATL1* | 0.174 | 0.067 | 0.278 | 1.646 × 10⁻⁰³ | 3.331 × 10⁻⁰² | 1.162 × 10⁻⁰¹ |
| *KRT85* | -0.178 | -0.282 | -0.071 | 1.267 × 10⁻⁰³ | 2.875 × 10⁻⁰² | 1.168 × 10⁻⁰¹ |
| *RBMXL2* | -0.173 | -0.277 | -0.066 | 1.745 × 10⁻⁰³ | 3.451 × 10⁻⁰² | 1.177 × 10⁻⁰¹ |
| *PDHA2* | -0.239 | -0.339 | -0.134 | 1.372 × 10⁻⁰⁵ | 2.695 × 10⁻⁰³ | 1.196 × 10⁻⁰¹ |
| *CTAGE5* | 0.168 | 0.060 | 0.272 | 2.464 × 10⁻⁰³ | 4.289 × 10⁻⁰² | 1.204 × 10⁻⁰¹ |
| *CBR1* | 0.174 | 0.066 | 0.278 | 1.674 × 10⁻⁰³ | 3.373 × 10⁻⁰² | 1.204 × 10⁻⁰¹ |
| *TSGA13* | -0.210 | -0.312 | -0.104 | 1.379 × 10⁻⁰⁴ | 1.022 × 10⁻⁰² | 1.226 × 10⁻⁰¹ |
| *DEPDC4* | -0.206 | -0.308 | -0.099 | 1.937 × 10⁻⁰⁴ | 1.195 × 10⁻⁰² | 1.229 × 10⁻⁰¹ |
| *RCOR2* | -0.181 | -0.284 | -0.073 | 1.072 × 10⁻⁰³ | 2.606 × 10⁻⁰² | 1.274 × 10⁻⁰¹ |
| *ACRV1* | -0.174 | -0.278 | -0.067 | 1.645 × 10⁻⁰³ | 3.331 × 10⁻⁰² | 1.278 × 10⁻⁰¹ |
| *CALCB* | -0.170 | -0.273 | -0.062 | 2.201 × 10⁻⁰³ | 3.994 × 10⁻⁰² | 1.278 × 10⁻⁰¹ |
| *TMEM196* | -0.214 | -0.316 | -0.108 | 1.022 × 10⁻⁰⁴ | 8.755 × 10⁻⁰³ | 1.284 × 10⁻⁰¹ |
| *SOX4* | -0.186 | -0.289 | -0.078 | 7.817 × 10⁻⁰⁴ | 2.210 × 10⁻⁰² | 1.284 × 10⁻⁰¹ |
| *HMOX1* | 0.193 | 0.086 | 0.296 | 4.740 × 10⁻⁰⁴ | 1.690 × 10⁻⁰² | 1.307 × 10⁻⁰¹ |
| *LOC284661* | -0.236 | -0.336 | -0.130 | 1.824 × 10⁻⁰⁵ | 3.284 × 10⁻⁰³ | 1.317 × 10⁻⁰¹ |
| *C17orf107* | -0.222 | -0.323 | -0.115 | 5.722 × 10⁻⁰⁵ | 6.362 × 10⁻⁰³ | 1.320 × 10⁻⁰¹ |
| *MRGPRX4* | -0.168 | -0.272 | -0.060 | 2.466 × 10⁻⁰³ | 4.289 × 10⁻⁰² | 1.321 × 10⁻⁰¹ |
| *LIN28B* | -0.192 | -0.295 | -0.085 | 5.172 × 10⁻⁰⁴ | 1.768 × 10⁻⁰² | 1.323 × 10⁻⁰¹ |
| *SYNPR* | -0.166 | -0.270 | -0.058 | 2.680 × 10⁻⁰³ | 4.515 × 10⁻⁰² | 1.326 × 10⁻⁰¹ |
| *PAX6* | -0.171 | -0.275 | -0.063 | 2.007 × 10⁻⁰³ | 3.774 × 10⁻⁰² | 1.333 × 10⁻⁰¹ |
| *USP28* | -0.196 | -0.298 | -0.089 | 3.895 × 10⁻⁰⁴ | 1.525 × 10⁻⁰² | 1.334 × 10⁻⁰¹ |
| *CDX2* | -0.187 | -0.290 | -0.079 | 7.254 × 10⁻⁰⁴ | 2.122 × 10⁻⁰² | 1.341 × 10⁻⁰¹ |
| *PAR1* | -0.192 | -0.294 | -0.084 | 5.221 × 10⁻⁰⁴ | 1.768 × 10⁻⁰² | 1.349 × 10⁻⁰¹ |
| *ZNF660* | -0.175 | -0.279 | -0.067 | 1.560 × 10⁻⁰³ | 3.214 × 10⁻⁰² | 1.367 × 10⁻⁰¹ |
| *PFN3* | -0.186 | -0.289 | -0.078 | 7.790 × 10⁻⁰⁴ | 2.210 × 10⁻⁰² | 1.383 × 10⁻⁰¹ |
| *PRPF6* | -0.235 | -0.336 | -0.130 | 1.891 × 10⁻⁰⁵ | 3.327 × 10⁻⁰³ | 1.391 × 10⁻⁰¹ |
| *LOC348021* | -0.196 | -0.299 | -0.089 | 3.781 × 10⁻⁰⁴ | 1.511 × 10⁻⁰² | 1.422 × 10⁻⁰¹ |
| *ASB10* | -0.177 | -0.280 | -0.069 | 1.391 × 10⁻⁰³ | 3.055 × 10⁻⁰² | 1.447 × 10⁻⁰¹ |
| *AGBL4* | -0.171 | -0.275 | -0.063 | 2.009 × 10⁻⁰³ | 3.774 × 10⁻⁰² | 1.450 × 10⁻⁰¹ |
| *EXOC7* | -0.180 | -0.283 | -0.072 | 1.171 × 10⁻⁰³ | 2.751 × 10⁻⁰² | 1.450 × 10⁻⁰¹ |
| *ZNF397* | -0.207 | -0.309 | -0.100 | 1.778 × 10⁻⁰⁴ | 1.167 × 10⁻⁰² | 1.455 × 10⁻⁰¹ |
| *PSD4* | 0.178 | 0.071 | 0.282 | 1.265 × 10⁻⁰³ | 2.875 × 10⁻⁰² | 1.465 × 10⁻⁰¹ |
| *SLC30A2* | -0.180 | -0.284 | -0.073 | 1.107 × 10⁻⁰³ | 2.658 × 10⁻⁰² | 1.466 × 10⁻⁰¹ |
| *SLC35F1* | -0.170 | -0.274 | -0.062 | 2.141 × 10⁻⁰³ | 3.914 × 10⁻⁰² | 1.510 × 10⁻⁰¹ |
| *CARD6* | 0.197 | 0.090 | 0.299 | 3.699 × 10⁻⁰⁴ | 1.488 × 10⁻⁰² | 1.522 × 10⁻⁰¹ |
| *HDAC2* | -0.196 | -0.299 | -0.089 | 3.853 × 10⁻⁰⁴ | 1.520 × 10⁻⁰² | 1.536 × 10⁻⁰¹ |
| *C9orf79* | -0.193 | -0.296 | -0.086 | 4.698 × 10⁻⁰⁴ | 1.690 × 10⁻⁰² | 1.549 × 10⁻⁰¹ |
| *ACTR5* | -0.183 | -0.287 | -0.076 | 9.130 × 10⁻⁰⁴ | 2.366 × 10⁻⁰² | 1.552 × 10⁻⁰¹ |
| *FGF23* | -0.194 | -0.297 | -0.087 | 4.506 × 10⁻⁰⁴ | 1.664 × 10⁻⁰² | 1.571 × 10⁻⁰¹ |
| *NBEA* | -0.170 | -0.274 | -0.063 | 2.082 × 10⁻⁰³ | 3.854 × 10⁻⁰² | 1.590 × 10⁻⁰¹ |
| *ADAM11* | -0.212 | -0.314 | -0.106 | 1.165 × 10⁻⁰⁴ | 9.279 × 10⁻⁰³ | 1.598 × 10⁻⁰¹ |
| *CRYAA* | -0.168 | -0.272 | -0.060 | 2.381 × 10⁻⁰³ | 4.222 × 10⁻⁰² | 1.603 × 10⁻⁰¹ |
| *DUSP21* | -0.169 | -0.273 | -0.061 | 2.300 × 10⁻⁰³ | 4.123 × 10⁻⁰² | 1.644 × 10⁻⁰¹ |
| *GPX6* | -0.325 | -0.419 | -0.224 | 2.151 × 10⁻⁰⁹ | 6.219 × 10⁻⁰⁶ | 1.663 × 10⁻⁰¹ |
| *THBD* | 0.201 | 0.094 | 0.303 | 2.733 × 10⁻⁰⁴ | 1.433 × 10⁻⁰² | 1.665 × 10⁻⁰¹ |
| *ZBED3* | -0.163 | -0.267 | -0.055 | 3.201 × 10⁻⁰³ | 4.989 × 10⁻⁰² | 1.668 × 10⁻⁰¹ |
| *SSX3* | -0.177 | -0.281 | -0.070 | 1.358 × 10⁻⁰³ | 3.016 × 10⁻⁰² | 1.689 × 10⁻⁰¹ |
| *KRTAP10.3* | -0.173 | -0.277 | -0.066 | 1.741 × 10⁻⁰³ | 3.448 × 10⁻⁰² | 1.697 × 10⁻⁰¹ |
| *ZNF498* | -0.170 | -0.274 | -0.063 | 2.086 × 10⁻⁰³ | 3.855 × 10⁻⁰² | 1.705 × 10⁻⁰¹ |
| *SUV420H1* | -0.211 | -0.312 | -0.104 | 1.340 × 10⁻⁰⁴ | 1.015 × 10⁻⁰² | 1.708 × 10⁻⁰¹ |
| *NONO* | -0.186 | -0.289 | -0.079 | 7.494 × 10⁻⁰⁴ | 2.163 × 10⁻⁰² | 1.724 × 10⁻⁰¹ |
| *BUD13* | -0.260 | -0.358 | -0.155 | 2.173 × 10⁻⁰⁶ | 8.792 × 10⁻⁰⁴ | 1.726 × 10⁻⁰¹ |
| *HOXD12* | -0.188 | -0.291 | -0.081 | 6.760 × 10⁻⁰⁴ | 2.042 × 10⁻⁰² | 1.747 × 10⁻⁰¹ |
| *PARP12* | 0.179 | 0.072 | 0.283 | 1.181 × 10⁻⁰³ | 2.759 × 10⁻⁰² | 1.750 × 10⁻⁰¹ |
| *C8orf22* | -0.221 | -0.322 | -0.115 | 6.005 × 10⁻⁰⁵ | 6.533 × 10⁻⁰³ | 1.762 × 10⁻⁰¹ |
| *ZNF34* | -0.174 | -0.278 | -0.067 | 1.640 × 10⁻⁰³ | 3.325 × 10⁻⁰² | 1.763 × 10⁻⁰¹ |
| *OR4D1* | -0.208 | -0.310 | -0.101 | 1.664 × 10⁻⁰⁴ | 1.130 × 10⁻⁰² | 1.776 × 10⁻⁰¹ |
| *NARF* | -0.185 | -0.288 | -0.077 | 8.336 × 10⁻⁰⁴ | 2.272 × 10⁻⁰² | 1.777 × 10⁻⁰¹ |
| *C14orf23* | -0.164 | -0.269 | -0.056 | 2.995 × 10⁻⁰³ | 4.803 × 10⁻⁰² | 1.784 × 10⁻⁰¹ |
| *DKFZP434K028* | -0.269 | -0.367 | -0.165 | 9.144 × 10⁻⁰⁷ | 5.428 × 10⁻⁰⁴ | 1.787 × 10⁻⁰¹ |
| *RNFT2* | -0.203 | -0.305 | -0.096 | 2.338 × 10⁻⁰⁴ | 1.324 × 10⁻⁰² | 1.817 × 10⁻⁰¹ |
| *YTHDF2* | -0.181 | -0.284 | -0.073 | 1.081 × 10⁻⁰³ | 2.622 × 10⁻⁰² | 1.858 × 10⁻⁰¹ |
| *TTC17* | -0.192 | -0.295 | -0.085 | 5.122 × 10⁻⁰⁴ | 1.760 × 10⁻⁰² | 1.862 × 10⁻⁰¹ |
| *OR52W1* | -0.193 | -0.296 | -0.086 | 4.764 × 10⁻⁰⁴ | 1.691 × 10⁻⁰² | 1.869 × 10⁻⁰¹ |
| *KIAA0195* | -0.172 | -0.276 | -0.065 | 1.853 × 10⁻⁰³ | 3.575 × 10⁻⁰² | 1.886 × 10⁻⁰¹ |
| *OR1L4* | -0.182 | -0.285 | -0.074 | 1.030 × 10⁻⁰³ | 2.555 × 10⁻⁰² | 1.892 × 10⁻⁰¹ |
| *DMRTB1* | -0.227 | -0.328 | -0.121 | 3.736 × 10⁻⁰⁵ | 4.878 × 10⁻⁰³ | 1.895 × 10⁻⁰¹ |
| *CCDC62* | -0.194 | -0.296 | -0.086 | 4.569 × 10⁻⁰⁴ | 1.666 × 10⁻⁰² | 1.903 × 10⁻⁰¹ |
| *RBM4* | -0.217 | -0.318 | -0.110 | 8.358 × 10⁻⁰⁵ | 7.793 × 10⁻⁰³ | 1.906 × 10⁻⁰¹ |
| *POLE* | -0.174 | -0.277 | -0.066 | 1.693 × 10⁻⁰³ | 3.386 × 10⁻⁰² | 1.909 × 10⁻⁰¹ |
| *CTSL3* | -0.167 | -0.271 | -0.059 | 2.618 × 10⁻⁰³ | 4.441 × 10⁻⁰² | 1.916 × 10⁻⁰¹ |
| *MYST2* | -0.173 | -0.277 | -0.065 | 1.754 × 10⁻⁰³ | 3.459 × 10⁻⁰² | 1.920 × 10⁻⁰¹ |
| *BEST3* | -0.169 | -0.273 | -0.061 | 2.272 × 10⁻⁰³ | 4.090 × 10⁻⁰² | 1.920 × 10⁻⁰¹ |
| *ZNF184* | -0.186 | -0.289 | -0.079 | 7.646 × 10⁻⁰⁴ | 2.194 × 10⁻⁰² | 1.929 × 10⁻⁰¹ |
| *SART3* | -0.211 | -0.312 | -0.104 | 1.339 × 10⁻⁰⁴ | 1.015 × 10⁻⁰² | 1.933 × 10⁻⁰¹ |
| *LOC100133985* | -0.168 | -0.272 | -0.060 | 2.384 × 10⁻⁰³ | 4.222 × 10⁻⁰² | 1.934 × 10⁻⁰¹ |
| *GABRG1* | -0.224 | -0.325 | -0.118 | 4.611 × 10⁻⁰⁵ | 5.521 × 10⁻⁰³ | 1.952 × 10⁻⁰¹ |
| *ARRDC4* | 0.203 | 0.096 | 0.305 | 2.349 × 10⁻⁰⁴ | 1.324 × 10⁻⁰² | 1.952 × 10⁻⁰¹ |
| *KCNJ11* | -0.181 | -0.285 | -0.074 | 1.032 × 10⁻⁰³ | 2.555 × 10⁻⁰² | 1.973 × 10⁻⁰¹ |
| *OR13F1* | -0.215 | -0.317 | -0.109 | 9.270 × 10⁻⁰⁵ | 8.401 × 10⁻⁰³ | 1.980 × 10⁻⁰¹ |
| *BRD8* | -0.165 | -0.269 | -0.057 | 2.865 × 10⁻⁰³ | 4.683 × 10⁻⁰² | 1.991 × 10⁻⁰¹ |
| *BCAS3* | -0.169 | -0.273 | -0.061 | 2.289 × 10⁻⁰³ | 4.116 × 10⁻⁰² | 2.022 × 10⁻⁰¹ |
| *KCNC2* | -0.242 | -0.342 | -0.137 | 1.054 × 10⁻⁰⁵ | 2.454 × 10⁻⁰³ | 2.023 × 10⁻⁰¹ |
| *RALB* | 0.209 | 0.102 | 0.311 | 1.508 × 10⁻⁰⁴ | 1.082 × 10⁻⁰² | 2.024 × 10⁻⁰¹ |
| *PAGE4* | -0.199 | -0.302 | -0.092 | 3.051 × 10⁻⁰⁴ | 1.433 × 10⁻⁰² | 2.033 × 10⁻⁰¹ |
| *IGFBP7* | 0.185 | 0.078 | 0.288 | 8.228 × 10⁻⁰⁴ | 2.256 × 10⁻⁰² | 2.036 × 10⁻⁰¹ |
| *OR11H12* | -0.213 | -0.315 | -0.107 | 1.098 × 10⁻⁰⁴ | 8.996 × 10⁻⁰³ | 2.062 × 10⁻⁰¹ |
| *VN1R2* | -0.209 | -0.311 | -0.102 | 1.542 × 10⁻⁰⁴ | 1.095 × 10⁻⁰² | 2.067 × 10⁻⁰¹ |
| *IQCF1* | -0.225 | -0.326 | -0.119 | 4.435 × 10⁻⁰⁵ | 5.465 × 10⁻⁰³ | 2.083 × 10⁻⁰¹ |
| *LOC285401* | -0.192 | -0.295 | -0.085 | 5.061 × 10⁻⁰⁴ | 1.751 × 10⁻⁰² | 2.087 × 10⁻⁰¹ |
| *CDHR5* | -0.177 | -0.281 | -0.070 | 1.345 × 10⁻⁰³ | 2.991 × 10⁻⁰² | 2.103 × 10⁻⁰¹ |
| *SI* | -0.179 | -0.282 | -0.071 | 1.216 × 10⁻⁰³ | 2.804 × 10⁻⁰² | 2.106 × 10⁻⁰¹ |
| *GPR81* | -0.172 | -0.276 | -0.065 | 1.837 × 10⁻⁰³ | 3.555 × 10⁻⁰² | 2.111 × 10⁻⁰¹ |
| *C17orf80* | -0.168 | -0.272 | -0.060 | 2.438 × 10⁻⁰³ | 4.269 × 10⁻⁰² | 2.114 × 10⁻⁰¹ |
| *PHGDH* | -0.191 | -0.294 | -0.084 | 5.511 × 10⁻⁰⁴ | 1.813 × 10⁻⁰² | 2.118 × 10⁻⁰¹ |
| *PAR.SN* | -0.164 | -0.269 | -0.057 | 2.981 × 10⁻⁰³ | 4.784 × 10⁻⁰² | 2.132 × 10⁻⁰¹ |
| *PRDM9* | -0.328 | -0.421 | -0.227 | 1.535 × 10⁻⁰⁹ | 5.358 × 10⁻⁰⁶ | 2.134 × 10⁻⁰¹ |
| *NEUROD1* | -0.218 | -0.319 | -0.112 | 7.618 × 10⁻⁰⁵ | 7.392 × 10⁻⁰³ | 2.137 × 10⁻⁰¹ |
| *C12orf39* | -0.234 | -0.334 | -0.128 | 2.143 × 10⁻⁰⁵ | 3.514 × 10⁻⁰³ | 2.142 × 10⁻⁰¹ |
| *GRID2IP* | -0.176 | -0.280 | -0.068 | 1.473 × 10⁻⁰³ | 3.122 × 10⁻⁰² | 2.160 × 10⁻⁰¹ |
| *NUDC* | -0.215 | -0.316 | -0.108 | 9.877 × 10⁻⁰⁵ | 8.652 × 10⁻⁰³ | 2.162 × 10⁻⁰¹ |
| *MRGPRX2* | -0.185 | -0.288 | -0.077 | 8.310 × 10⁻⁰⁴ | 2.269 × 10⁻⁰² | 2.169 × 10⁻⁰¹ |
| *MPI* | -0.168 | -0.272 | -0.060 | 2.484 × 10⁻⁰³ | 4.289 × 10⁻⁰² | 2.169 × 10⁻⁰¹ |
| *SLC7A14* | -0.230 | -0.331 | -0.124 | 2.885 × 10⁻⁰⁵ | 4.262 × 10⁻⁰³ | 2.188 × 10⁻⁰¹ |
| *OR8D4* | -0.308 | -0.403 | -0.206 | 1.565 × 10⁻⁰⁸ | 2.880 × 10⁻⁰⁵ | 2.201 × 10⁻⁰¹ |
| *SF3A1* | -0.195 | -0.298 | -0.088 | 4.072 × 10⁻⁰⁴ | 1.561 × 10⁻⁰² | 2.205 × 10⁻⁰¹ |
| *DMRTC2* | -0.184 | -0.287 | -0.076 | 8.868 × 10⁻⁰⁴ | 2.318 × 10⁻⁰² | 2.218 × 10⁻⁰¹ |
| *LYSMD2* | 0.168 | 0.060 | 0.272 | 2.395 × 10⁻⁰³ | 4.236 × 10⁻⁰² | 2.238 × 10⁻⁰¹ |
| *OR52E6* | -0.181 | -0.284 | -0.073 | 1.076 × 10⁻⁰³ | 2.613 × 10⁻⁰² | 2.241 × 10⁻⁰¹ |
| *SFPQ* | -0.205 | -0.307 | -0.098 | 2.055 × 10⁻⁰⁴ | 1.234 × 10⁻⁰² | 2.247 × 10⁻⁰¹ |
| *OR2T11* | -0.177 | -0.280 | -0.069 | 1.392 × 10⁻⁰³ | 3.055 × 10⁻⁰² | 2.251 × 10⁻⁰¹ |
| *PEA15* | 0.167 | 0.059 | 0.271 | 2.521 × 10⁻⁰³ | 4.338 × 10⁻⁰² | 2.256 × 10⁻⁰¹ |
| *CHRFAM7A* | -0.183 | -0.286 | -0.076 | 9.353 × 10⁻⁰⁴ | 2.402 × 10⁻⁰² | 2.261 × 10⁻⁰¹ |
| *HAPLN4* | -0.183 | -0.286 | -0.075 | 9.608 × 10⁻⁰⁴ | 2.447 × 10⁻⁰² | 2.273 × 10⁻⁰¹ |
| *C1orf185* | -0.170 | -0.274 | -0.062 | 2.189 × 10⁻⁰³ | 3.984 × 10⁻⁰² | 2.284 × 10⁻⁰¹ |
| *OR2H2* | -0.216 | -0.317 | -0.109 | 8.985 × 10⁻⁰⁵ | 8.227 × 10⁻⁰³ | 2.294 × 10⁻⁰¹ |
| *MKS1* | -0.186 | -0.289 | -0.078 | 7.928 × 10⁻⁰⁴ | 2.222 × 10⁻⁰² | 2.311 × 10⁻⁰¹ |
| *SLC2A4* | -0.171 | -0.275 | -0.064 | 1.955 × 10⁻⁰³ | 3.710 × 10⁻⁰² | 2.344 × 10⁻⁰¹ |
| *LOC440040* | -0.164 | -0.268 | -0.056 | 3.148 × 10⁻⁰³ | 4.943 × 10⁻⁰² | 2.346 × 10⁻⁰¹ |
| *RASGEF1C* | -0.169 | -0.273 | -0.061 | 2.255 × 10⁻⁰³ | 4.070 × 10⁻⁰² | 2.350 × 10⁻⁰¹ |
| *HDDC3* | -0.166 | -0.270 | -0.058 | 2.780 × 10⁻⁰³ | 4.603 × 10⁻⁰² | 2.351 × 10⁻⁰¹ |
| *MBTD1* | -0.173 | -0.277 | -0.066 | 1.733 × 10⁻⁰³ | 3.448 × 10⁻⁰² | 2.352 × 10⁻⁰¹ |
| *CPEB2* | 0.203 | 0.096 | 0.305 | 2.311 × 10⁻⁰⁴ | 1.319 × 10⁻⁰² | 2.361 × 10⁻⁰¹ |
| *TFAMP1* | -0.189 | -0.292 | -0.082 | 6.209 × 10⁻⁰⁴ | 1.930 × 10⁻⁰² | 2.369 × 10⁻⁰¹ |
| *PRDM7* | -0.218 | -0.319 | -0.111 | 7.810 × 10⁻⁰⁵ | 7.453 × 10⁻⁰³ | 2.385 × 10⁻⁰¹ |
| *AKIRIN1* | -0.166 | -0.270 | -0.058 | 2.793 × 10⁻⁰³ | 4.607 × 10⁻⁰² | 2.392 × 10⁻⁰¹ |
| *OR6B1* | -0.167 | -0.271 | -0.059 | 2.630 × 10⁻⁰³ | 4.453 × 10⁻⁰² | 2.396 × 10⁻⁰¹ |
| *KCTD9* | 0.202 | 0.095 | 0.304 | 2.490 × 10⁻⁰⁴ | 1.366 × 10⁻⁰² | 2.396 × 10⁻⁰¹ |
| *LOC149134* | -0.171 | -0.275 | -0.063 | 1.984 × 10⁻⁰³ | 3.748 × 10⁻⁰² | 2.399 × 10⁻⁰¹ |
| *GLYATL1* | -0.208 | -0.310 | -0.101 | 1.656 × 10⁻⁰⁴ | 1.130 × 10⁻⁰² | 2.414 × 10⁻⁰¹ |
| *C11orf36* | -0.298 | -0.394 | -0.195 | 4.554 × 10⁻⁰⁸ | 6.221 × 10⁻⁰⁵ | 2.414 × 10⁻⁰¹ |
| *RAD21L1* | -0.166 | -0.270 | -0.058 | 2.679 × 10⁻⁰³ | 4.515 × 10⁻⁰² | 2.423 × 10⁻⁰¹ |
| *OR2J3* | -0.233 | -0.334 | -0.128 | 2.188 × 10⁻⁰⁵ | 3.514 × 10⁻⁰³ | 2.427 × 10⁻⁰¹ |
| *UGT2B11* | -0.179 | -0.283 | -0.072 | 1.198 × 10⁻⁰³ | 2.784 × 10⁻⁰² | 2.434 × 10⁻⁰¹ |
| *CLDN19* | -0.234 | -0.334 | -0.128 | 2.112 × 10⁻⁰⁵ | 3.514 × 10⁻⁰³ | 2.435 × 10⁻⁰¹ |
| *GPHA2* | -0.176 | -0.279 | -0.068 | 1.503 × 10⁻⁰³ | 3.150 × 10⁻⁰² | 2.438 × 10⁻⁰¹ |
| *PROKR1* | -0.263 | -0.362 | -0.159 | 1.538 × 10⁻⁰⁶ | 7.318 × 10⁻⁰⁴ | 2.453 × 10⁻⁰¹ |
| *CAPRIN1* | -0.174 | -0.278 | -0.066 | 1.668 × 10⁻⁰³ | 3.364 × 10⁻⁰² | 2.456 × 10⁻⁰¹ |
| *HCN1* | -0.206 | -0.308 | -0.099 | 1.931 × 10⁻⁰⁴ | 1.195 × 10⁻⁰² | 2.467 × 10⁻⁰¹ |
| *TMEM201* | -0.175 | -0.279 | -0.068 | 1.529 × 10⁻⁰³ | 3.178 × 10⁻⁰² | 2.469 × 10⁻⁰¹ |
| *TFAP2B* | -0.211 | -0.313 | -0.104 | 1.296 × 10⁻⁰⁴ | 9.975 × 10⁻⁰³ | 2.487 × 10⁻⁰¹ |
| *HRH1* | 0.186 | 0.078 | 0.289 | 7.768 × 10⁻⁰⁴ | 2.210 × 10⁻⁰² | 2.494 × 10⁻⁰¹ |
| *OR3A3* | -0.169 | -0.273 | -0.061 | 2.334 × 10⁻⁰³ | 4.158 × 10⁻⁰² | 2.495 × 10⁻⁰¹ |
| *FAM138B* | -0.184 | -0.287 | -0.077 | 8.674 × 10⁻⁰⁴ | 2.301 × 10⁻⁰² | 2.495 × 10⁻⁰¹ |
| *FUS* | -0.201 | -0.303 | -0.094 | 2.741 × 10⁻⁰⁴ | 1.433 × 10⁻⁰² | 2.532 × 10⁻⁰¹ |
| *BRSK2* | -0.172 | -0.276 | -0.064 | 1.881 × 10⁻⁰³ | 3.605 × 10⁻⁰² | 2.554 × 10⁻⁰¹ |
| *MYOD1* | -0.199 | -0.301 | -0.092 | 3.208 × 10⁻⁰⁴ | 1.433 × 10⁻⁰² | 2.572 × 10⁻⁰¹ |
| *MAP4K2* | -0.185 | -0.288 | -0.078 | 8.193 × 10⁻⁰⁴ | 2.252 × 10⁻⁰² | 2.581 × 10⁻⁰¹ |
| *MUC6* | -0.208 | -0.310 | -0.102 | 1.599 × 10⁻⁰⁴ | 1.127 × 10⁻⁰² | 2.584 × 10⁻⁰¹ |
| *MFSD1* | 0.214 | 0.108 | 0.316 | 1.017 × 10⁻⁰⁴ | 8.755 × 10⁻⁰³ | 2.586 × 10⁻⁰¹ |
| *KRTAP10.2* | -0.190 | -0.292 | -0.082 | 6.045 × 10⁻⁰⁴ | 1.911 × 10⁻⁰² | 2.600 × 10⁻⁰¹ |
| *TRPC7* | -0.187 | -0.290 | -0.080 | 7.178 × 10⁻⁰⁴ | 2.111 × 10⁻⁰² | 2.605 × 10⁻⁰¹ |
| *SLMAP* | 0.195 | 0.088 | 0.298 | 4.105 × 10⁻⁰⁴ | 1.564 × 10⁻⁰² | 2.631 × 10⁻⁰¹ |
| *SLC27A6* | -0.170 | -0.274 | -0.062 | 2.144 × 10⁻⁰³ | 3.915 × 10⁻⁰² | 2.635 × 10⁻⁰¹ |
| *GNA15* | 0.265 | 0.161 | 0.363 | 1.321 × 10⁻⁰⁶ | 6.852 × 10⁻⁰⁴ | 2.638 × 10⁻⁰¹ |
| *GAL3ST3* | -0.257 | -0.356 | -0.152 | 2.848 × 10⁻⁰⁶ | 1.067 × 10⁻⁰³ | 2.644 × 10⁻⁰¹ |
| *GABRA4* | -0.298 | -0.394 | -0.195 | 4.612 × 10⁻⁰⁸ | 6.221 × 10⁻⁰⁵ | 2.655 × 10⁻⁰¹ |
| *ZMAT4* | -0.187 | -0.290 | -0.080 | 6.964 × 10⁻⁰⁴ | 2.069 × 10⁻⁰² | 2.668 × 10⁻⁰¹ |
| *ANKRD33* | -0.281 | -0.379 | -0.178 | 2.583 × 10⁻⁰⁷ | 2.375 × 10⁻⁰⁴ | 2.672 × 10⁻⁰¹ |
| *PHF11* | 0.183 | 0.075 | 0.286 | 9.549 × 10⁻⁰⁴ | 2.437 × 10⁻⁰² | 2.679 × 10⁻⁰¹ |
| *FKSG83* | -0.207 | -0.309 | -0.100 | 1.737 × 10⁻⁰⁴ | 1.149 × 10⁻⁰² | 2.698 × 10⁻⁰¹ |
| *CYLC1* | -0.258 | -0.357 | -0.154 | 2.457 × 10⁻⁰⁶ | 9.748 × 10⁻⁰⁴ | 2.717 × 10⁻⁰¹ |
| *RIT2* | -0.197 | -0.300 | -0.090 | 3.541 × 10⁻⁰⁴ | 1.454 × 10⁻⁰² | 2.719 × 10⁻⁰¹ |
| *GHRH* | -0.213 | -0.315 | -0.107 | 1.081 × 10⁻⁰⁴ | 8.931 × 10⁻⁰³ | 2.733 × 10⁻⁰¹ |
| *PALMD* | 0.168 | 0.060 | 0.272 | 2.378 × 10⁻⁰³ | 4.220 × 10⁻⁰² | 2.751 × 10⁻⁰¹ |
| *SLC2A9* | 0.178 | 0.070 | 0.281 | 1.309 × 10⁻⁰³ | 2.943 × 10⁻⁰² | 2.754 × 10⁻⁰¹ |
| *DBX1* | -0.195 | -0.297 | -0.088 | 4.246 × 10⁻⁰⁴ | 1.603 × 10⁻⁰² | 2.762 × 10⁻⁰¹ |
| *KHDRBS1* | -0.235 | -0.335 | -0.129 | 1.956 × 10⁻⁰⁵ | 3.411 × 10⁻⁰³ | 2.765 × 10⁻⁰¹ |
| *ATCAY* | -0.258 | -0.357 | -0.153 | 2.549 × 10⁻⁰⁶ | 9.753 × 10⁻⁰⁴ | 2.768 × 10⁻⁰¹ |
| *PRR3* | -0.182 | -0.285 | -0.074 | 1.003 × 10⁻⁰³ | 2.511 × 10⁻⁰² | 2.775 × 10⁻⁰¹ |
| *AMOT* | -0.172 | -0.275 | -0.064 | 1.931 × 10⁻⁰³ | 3.676 × 10⁻⁰² | 2.780 × 10⁻⁰¹ |
| *LOC441666* | -0.165 | -0.269 | -0.057 | 2.941 × 10⁻⁰³ | 4.753 × 10⁻⁰² | 2.793 × 10⁻⁰¹ |
| *LSM14B* | -0.250 | -0.349 | -0.145 | 5.315 × 10⁻⁰⁶ | 1.707 × 10⁻⁰³ | 2.811 × 10⁻⁰¹ |
| *RGAG1* | -0.181 | -0.285 | -0.074 | 1.049 × 10⁻⁰³ | 2.570 × 10⁻⁰² | 2.828 × 10⁻⁰¹ |
| *MAPRE3* | 0.165 | 0.057 | 0.269 | 2.961 × 10⁻⁰³ | 4.779 × 10⁻⁰² | 2.831 × 10⁻⁰¹ |
| *RAX* | -0.213 | -0.315 | -0.106 | 1.124 × 10⁻⁰⁴ | 9.171 × 10⁻⁰³ | 2.844 × 10⁻⁰¹ |
| *LECT1* | -0.190 | -0.293 | -0.083 | 5.840 × 10⁻⁰⁴ | 1.873 × 10⁻⁰² | 2.854 × 10⁻⁰¹ |
| *EIF2C1* | -0.209 | -0.311 | -0.102 | 1.516 × 10⁻⁰⁴ | 1.084 × 10⁻⁰² | 2.878 × 10⁻⁰¹ |
| *TEX13B* | -0.268 | -0.367 | -0.164 | 9.528 × 10⁻⁰⁷ | 5.428 × 10⁻⁰⁴ | 2.887 × 10⁻⁰¹ |
| *AKR7A3* | -0.173 | -0.277 | -0.066 | 1.741 × 10⁻⁰³ | 3.448 × 10⁻⁰² | 2.901 × 10⁻⁰¹ |
| *UBTFL1* | -0.200 | -0.303 | -0.093 | 2.832 × 10⁻⁰⁴ | 1.433 × 10⁻⁰² | 2.902 × 10⁻⁰¹ |
| *NAIF1* | -0.202 | -0.305 | -0.095 | 2.473 × 10⁻⁰⁴ | 1.363 × 10⁻⁰² | 2.908 × 10⁻⁰¹ |
| *HNRNPUL1* | -0.178 | -0.282 | -0.071 | 1.266 × 10⁻⁰³ | 2.875 × 10⁻⁰² | 2.912 × 10⁻⁰¹ |
| *NASP* | -0.227 | -0.328 | -0.121 | 3.707 × 10⁻⁰⁵ | 4.872 × 10⁻⁰³ | 2.924 × 10⁻⁰¹ |
| *OLFM1* | 0.179 | 0.071 | 0.282 | 1.218 × 10⁻⁰³ | 2.804 × 10⁻⁰² | 2.951 × 10⁻⁰¹ |
| *SNORD116.28* | -0.165 | -0.269 | -0.057 | 2.867 × 10⁻⁰³ | 4.683 × 10⁻⁰² | 2.959 × 10⁻⁰¹ |
| *LOC100128076* | -0.241 | -0.341 | -0.135 | 1.183 × 10⁻⁰⁵ | 2.478 × 10⁻⁰³ | 2.972 × 10⁻⁰¹ |
| *ANKRD34B* | -0.164 | -0.268 | -0.056 | 3.087 × 10⁻⁰³ | 4.876 × 10⁻⁰² | 2.981 × 10⁻⁰¹ |
| *USP6* | -0.190 | -0.293 | -0.082 | 5.959 × 10⁻⁰⁴ | 1.901 × 10⁻⁰² | 2.986 × 10⁻⁰¹ |
| *OPN5* | -0.168 | -0.272 | -0.060 | 2.445 × 10⁻⁰³ | 4.273 × 10⁻⁰² | 2.990 × 10⁻⁰¹ |
| *GPR6* | -0.218 | -0.319 | -0.112 | 7.550 × 10⁻⁰⁵ | 7.392 × 10⁻⁰³ | 2.994 × 10⁻⁰¹ |
| *TRPC5* | -0.218 | -0.320 | -0.112 | 7.482 × 10⁻⁰⁵ | 7.392 × 10⁻⁰³ | 3.013 × 10⁻⁰¹ |
| *FEZF1* | -0.184 | -0.287 | -0.076 | 8.824 × 10⁻⁰⁴ | 2.312 × 10⁻⁰² | 3.017 × 10⁻⁰¹ |
| *FAM66A* | -0.167 | -0.271 | -0.059 | 2.534 × 10⁻⁰³ | 4.348 × 10⁻⁰² | 3.024 × 10⁻⁰¹ |
| *DVL2* | -0.181 | -0.284 | -0.073 | 1.085 × 10⁻⁰³ | 2.628 × 10⁻⁰² | 3.030 × 10⁻⁰¹ |
| *OR6K6* | -0.171 | -0.275 | -0.063 | 1.995 × 10⁻⁰³ | 3.762 × 10⁻⁰² | 3.038 × 10⁻⁰¹ |
| *TAS2R9* | -0.172 | -0.276 | -0.064 | 1.902 × 10⁻⁰³ | 3.631 × 10⁻⁰² | 3.052 × 10⁻⁰¹ |
| *C6orf134* | -0.197 | -0.299 | -0.090 | 3.622 × 10⁻⁰⁴ | 1.476 × 10⁻⁰² | 3.060 × 10⁻⁰¹ |
| *WNT10B* | -0.185 | -0.288 | -0.078 | 8.150 × 10⁻⁰⁴ | 2.247 × 10⁻⁰² | 3.073 × 10⁻⁰¹ |
| *CREG1* | 0.187 | 0.079 | 0.290 | 7.298 × 10⁻⁰⁴ | 2.129 × 10⁻⁰² | 3.084 × 10⁻⁰¹ |
| *OTP* | -0.182 | -0.285 | -0.075 | 9.991 × 10⁻⁰⁴ | 2.505 × 10⁻⁰² | 3.085 × 10⁻⁰¹ |
| *KBTBD5* | -0.196 | -0.298 | -0.088 | 3.987 × 10⁻⁰⁴ | 1.542 × 10⁻⁰² | 3.122 × 10⁻⁰¹ |
| *ZBTB39* | -0.194 | -0.297 | -0.087 | 4.323 × 10⁻⁰⁴ | 1.626 × 10⁻⁰² | 3.153 × 10⁻⁰¹ |
| *AIPL1* | -0.179 | -0.282 | -0.071 | 1.247 × 10⁻⁰³ | 2.856 × 10⁻⁰² | 3.154 × 10⁻⁰¹ |
| *SLC17A6* | -0.191 | -0.294 | -0.084 | 5.357 × 10⁻⁰⁴ | 1.783 × 10⁻⁰² | 3.162 × 10⁻⁰¹ |
| *ALDOC* | -0.176 | -0.280 | -0.069 | 1.430 × 10⁻⁰³ | 3.078 × 10⁻⁰² | 3.167 × 10⁻⁰¹ |
| *CNTROB* | -0.185 | -0.288 | -0.078 | 8.232 × 10⁻⁰⁴ | 2.256 × 10⁻⁰² | 3.186 × 10⁻⁰¹ |
| *FGFBP3* | -0.181 | -0.285 | -0.074 | 1.044 × 10⁻⁰³ | 2.570 × 10⁻⁰² | 3.197 × 10⁻⁰¹ |
| *STOX1* | -0.163 | -0.268 | -0.055 | 3.192 × 10⁻⁰³ | 4.984 × 10⁻⁰² | 3.208 × 10⁻⁰¹ |
| *ACTR6* | -0.212 | -0.314 | -0.106 | 1.170 × 10⁻⁰⁴ | 9.280 × 10⁻⁰³ | 3.233 × 10⁻⁰¹ |
| *KRT12* | -0.240 | -0.340 | -0.134 | 1.268 × 10⁻⁰⁵ | 2.593 × 10⁻⁰³ | 3.243 × 10⁻⁰¹ |
| *NXF4* | -0.231 | -0.332 | -0.125 | 2.694 × 10⁻⁰⁵ | 4.099 × 10⁻⁰³ | 3.287 × 10⁻⁰¹ |
| *FUT2* | 0.186 | 0.078 | 0.289 | 7.765 × 10⁻⁰⁴ | 2.210 × 10⁻⁰² | 3.292 × 10⁻⁰¹ |
| *USP11* | -0.219 | -0.321 | -0.113 | 6.846 × 10⁻⁰⁵ | 7.131 × 10⁻⁰³ | 3.303 × 10⁻⁰¹ |
| *OR5E1P* | -0.215 | -0.317 | -0.109 | 9.320 × 10⁻⁰⁵ | 8.401 × 10⁻⁰³ | 3.308 × 10⁻⁰¹ |
| *DDX25* | -0.242 | -0.342 | -0.136 | 1.086 × 10⁻⁰⁵ | 2.466 × 10⁻⁰³ | 3.350 × 10⁻⁰¹ |
| *PODXL2* | -0.189 | -0.292 | -0.081 | 6.415 × 10⁻⁰⁴ | 1.976 × 10⁻⁰² | 3.355 × 10⁻⁰¹ |
| *LOC340094* | -0.166 | -0.270 | -0.058 | 2.686 × 10⁻⁰³ | 4.518 × 10⁻⁰² | 3.358 × 10⁻⁰¹ |
| *GH2* | -0.250 | -0.349 | -0.145 | 5.285 × 10⁻⁰⁶ | 1.707 × 10⁻⁰³ | 3.363 × 10⁻⁰¹ |
| *BAT3* | -0.247 | -0.347 | -0.142 | 6.813 × 10⁻⁰⁶ | 2.006 × 10⁻⁰³ | 3.375 × 10⁻⁰¹ |
| *MYF6* | -0.167 | -0.271 | -0.059 | 2.536 × 10⁻⁰³ | 4.348 × 10⁻⁰² | 3.379 × 10⁻⁰¹ |
| *C2orf52* | -0.192 | -0.295 | -0.085 | 5.207 × 10⁻⁰⁴ | 1.768 × 10⁻⁰² | 3.383 × 10⁻⁰¹ |
| *FANCF* | -0.203 | -0.305 | -0.096 | 2.433 × 10⁻⁰⁴ | 1.349 × 10⁻⁰² | 3.384 × 10⁻⁰¹ |
| *GABRB1* | -0.193 | -0.295 | -0.085 | 4.928 × 10⁻⁰⁴ | 1.726 × 10⁻⁰² | 3.385 × 10⁻⁰¹ |
| *RBM10* | -0.203 | -0.305 | -0.096 | 2.312 × 10⁻⁰⁴ | 1.319 × 10⁻⁰² | 3.388 × 10⁻⁰¹ |
| *SETD7* | 0.225 | 0.119 | 0.326 | 4.422 × 10⁻⁰⁵ | 5.465 × 10⁻⁰³ | 3.392 × 10⁻⁰¹ |
| *ATXN7L3B* | -0.185 | -0.288 | -0.078 | 8.157 × 10⁻⁰⁴ | 2.247 × 10⁻⁰² | 3.418 × 10⁻⁰¹ |
| *AGXT* | -0.213 | -0.314 | -0.106 | 1.149 × 10⁻⁰⁴ | 9.232 × 10⁻⁰³ | 3.441 × 10⁻⁰¹ |
| *LOC645752* | -0.191 | -0.294 | -0.084 | 5.572 × 10⁻⁰⁴ | 1.821 × 10⁻⁰² | 3.448 × 10⁻⁰¹ |
| *LOC284788* | -0.166 | -0.270 | -0.058 | 2.721 × 10⁻⁰³ | 4.553 × 10⁻⁰² | 3.488 × 10⁻⁰¹ |
| *UBQLN3* | -0.191 | -0.294 | -0.084 | 5.408 × 10⁻⁰⁴ | 1.794 × 10⁻⁰² | 3.499 × 10⁻⁰¹ |
| *NCL* | -0.176 | -0.279 | -0.068 | 1.493 × 10⁻⁰³ | 3.140 × 10⁻⁰² | 3.508 × 10⁻⁰¹ |
| *TRIM39* | -0.197 | -0.299 | -0.090 | 3.640 × 10⁻⁰⁴ | 1.476 × 10⁻⁰² | 3.509 × 10⁻⁰¹ |
| *C3orf20* | -0.166 | -0.270 | -0.058 | 2.662 × 10⁻⁰³ | 4.500 × 10⁻⁰² | 3.515 × 10⁻⁰¹ |
| *RFPL4B* | -0.212 | -0.313 | -0.105 | 1.229 × 10⁻⁰⁴ | 9.632 × 10⁻⁰³ | 3.521 × 10⁻⁰¹ |
| *PCDHB19P* | -0.165 | -0.269 | -0.057 | 2.970 × 10⁻⁰³ | 4.781 × 10⁻⁰² | 3.557 × 10⁻⁰¹ |
| *KIAA1467* | -0.182 | -0.285 | -0.074 | 1.031 × 10⁻⁰³ | 2.555 × 10⁻⁰² | 3.561 × 10⁻⁰¹ |
| *SLC14A2* | -0.166 | -0.270 | -0.058 | 2.716 × 10⁻⁰³ | 4.549 × 10⁻⁰² | 3.563 × 10⁻⁰¹ |
| *ACTL6B* | -0.170 | -0.274 | -0.062 | 2.133 × 10⁻⁰³ | 3.907 × 10⁻⁰² | 3.563 × 10⁻⁰¹ |
| *KRTAP5.4* | -0.220 | -0.322 | -0.114 | 6.280 × 10⁻⁰⁵ | 6.724 × 10⁻⁰³ | 3.568 × 10⁻⁰¹ |
| *PREP* | -0.223 | -0.324 | -0.117 | 5.105 × 10⁻⁰⁵ | 5.903 × 10⁻⁰³ | 3.573 × 10⁻⁰¹ |
| *COL11A2* | -0.173 | -0.277 | -0.065 | 1.758 × 10⁻⁰³ | 3.463 × 10⁻⁰² | 3.582 × 10⁻⁰¹ |
| *C19orf10* | 0.174 | 0.066 | 0.278 | 1.677 × 10⁻⁰³ | 3.374 × 10⁻⁰² | 3.627 × 10⁻⁰¹ |
| *SMARCE1* | -0.192 | -0.295 | -0.085 | 4.968 × 10⁻⁰⁴ | 1.727 × 10⁻⁰² | 3.636 × 10⁻⁰¹ |
| *TYMP* | 0.188 | 0.081 | 0.291 | 6.494 × 10⁻⁰⁴ | 1.988 × 10⁻⁰² | 3.641 × 10⁻⁰¹ |
| *TAS2R30* | -0.171 | -0.275 | -0.063 | 1.984 × 10⁻⁰³ | 3.748 × 10⁻⁰² | 3.644 × 10⁻⁰¹ |
| *CCDC23* | -0.169 | -0.273 | -0.061 | 2.248 × 10⁻⁰³ | 4.064 × 10⁻⁰² | 3.654 × 10⁻⁰¹ |
| *OR1E1* | -0.229 | -0.330 | -0.124 | 3.055 × 10⁻⁰⁵ | 4.415 × 10⁻⁰³ | 3.663 × 10⁻⁰¹ |
| *CWC27* | -0.166 | -0.270 | -0.058 | 2.754 × 10⁻⁰³ | 4.579 × 10⁻⁰² | 3.694 × 10⁻⁰¹ |
| *NAA20* | 0.226 | 0.120 | 0.327 | 4.153 × 10⁻⁰⁵ | 5.230 × 10⁻⁰³ | 3.704 × 10⁻⁰¹ |
| *HRK* | -0.184 | -0.287 | -0.077 | 8.632 × 10⁻⁰⁴ | 2.298 × 10⁻⁰² | 3.706 × 10⁻⁰¹ |
| *ARF4* | 0.178 | 0.070 | 0.281 | 1.331 × 10⁻⁰³ | 2.966 × 10⁻⁰² | 3.719 × 10⁻⁰¹ |
| *TMEM39B* | -0.200 | -0.302 | -0.093 | 2.933 × 10⁻⁰⁴ | 1.433 × 10⁻⁰² | 3.745 × 10⁻⁰¹ |
| *ITFG2* | -0.188 | -0.291 | -0.080 | 6.935 × 10⁻⁰⁴ | 2.069 × 10⁻⁰² | 3.754 × 10⁻⁰¹ |
| *NUDT18* | 0.175 | 0.067 | 0.279 | 1.554 × 10⁻⁰³ | 3.206 × 10⁻⁰² | 3.767 × 10⁻⁰¹ |
| *TBC1D26* | -0.181 | -0.285 | -0.074 | 1.037 × 10⁻⁰³ | 2.562 × 10⁻⁰² | 3.786 × 10⁻⁰¹ |
| *CT47B1* | -0.167 | -0.271 | -0.059 | 2.591 × 10⁻⁰³ | 4.416 × 10⁻⁰² | 3.794 × 10⁻⁰¹ |
| *KRTAP5.3* | -0.219 | -0.321 | -0.113 | 6.901 × 10⁻⁰⁵ | 7.131 × 10⁻⁰³ | 3.808 × 10⁻⁰¹ |
| *GRIK5* | -0.181 | -0.284 | -0.073 | 1.088 × 10⁻⁰³ | 2.629 × 10⁻⁰² | 3.835 × 10⁻⁰¹ |
| *DPH1* | -0.180 | -0.283 | -0.072 | 1.140 × 10⁻⁰³ | 2.721 × 10⁻⁰² | 3.852 × 10⁻⁰¹ |
| *ZCCHC12* | -0.228 | -0.329 | -0.122 | 3.456 × 10⁻⁰⁵ | 4.662 × 10⁻⁰³ | 3.858 × 10⁻⁰¹ |
| *CSH1* | -0.215 | -0.316 | -0.109 | 9.639 × 10⁻⁰⁵ | 8.517 × 10⁻⁰³ | 3.866 × 10⁻⁰¹ |
| *GJA8* | -0.165 | -0.269 | -0.057 | 2.968 × 10⁻⁰³ | 4.781 × 10⁻⁰² | 3.876 × 10⁻⁰¹ |
| *COX6A2* | -0.174 | -0.278 | -0.067 | 1.621 × 10⁻⁰³ | 3.300 × 10⁻⁰² | 3.882 × 10⁻⁰¹ |
| *HCRTR2* | -0.258 | -0.357 | -0.153 | 2.555 × 10⁻⁰⁶ | 9.753 × 10⁻⁰⁴ | 3.883 × 10⁻⁰¹ |
| *PCYT2* | -0.179 | -0.283 | -0.072 | 1.200 × 10⁻⁰³ | 2.785 × 10⁻⁰² | 3.886 × 10⁻⁰¹ |
| *FAM75C1* | -0.179 | -0.283 | -0.072 | 1.180 × 10⁻⁰³ | 2.759 × 10⁻⁰² | 3.892 × 10⁻⁰¹ |
| *OR5P3* | -0.172 | -0.276 | -0.065 | 1.835 × 10⁻⁰³ | 3.555 × 10⁻⁰² | 3.898 × 10⁻⁰¹ |
| *LOC541471* | 0.207 | 0.100 | 0.309 | 1.737 × 10⁻⁰⁴ | 1.149 × 10⁻⁰² | 3.901 × 10⁻⁰¹ |
| *OSBPL3* | 0.236 | 0.131 | 0.337 | 1.723 × 10⁻⁰⁵ | 3.199 × 10⁻⁰³ | 3.927 × 10⁻⁰¹ |
| *DPPA5* | -0.164 | -0.268 | -0.056 | 3.080 × 10⁻⁰³ | 4.869 × 10⁻⁰² | 3.968 × 10⁻⁰¹ |
| *ATP2B3* | -0.206 | -0.308 | -0.099 | 1.884 × 10⁻⁰⁴ | 1.194 × 10⁻⁰² | 4.001 × 10⁻⁰¹ |
| *PRM2* | -0.234 | -0.334 | -0.128 | 2.109 × 10⁻⁰⁵ | 3.514 × 10⁻⁰³ | 4.023 × 10⁻⁰¹ |
| *TMEM154* | 0.184 | 0.076 | 0.287 | 8.925 × 10⁻⁰⁴ | 2.327 × 10⁻⁰² | 4.028 × 10⁻⁰¹ |
| *YIPF7* | -0.172 | -0.276 | -0.064 | 1.866 × 10⁻⁰³ | 3.589 × 10⁻⁰² | 4.034 × 10⁻⁰¹ |
| *CCDC163P* | -0.168 | -0.272 | -0.060 | 2.418 × 10⁻⁰³ | 4.260 × 10⁻⁰² | 4.036 × 10⁻⁰¹ |
| *ATP7B* | -0.179 | -0.283 | -0.072 | 1.197 × 10⁻⁰³ | 2.784 × 10⁻⁰² | 4.038 × 10⁻⁰¹ |
| *ZNF516* | -0.200 | -0.302 | -0.093 | 2.919 × 10⁻⁰⁴ | 1.433 × 10⁻⁰² | 4.044 × 10⁻⁰¹ |
| *UGT3A1* | -0.313 | -0.408 | -0.212 | 8.290 × 10⁻⁰⁹ | 1.866 × 10⁻⁰⁵ | 4.055 × 10⁻⁰¹ |
| *UBE2Q2* | 0.228 | 0.122 | 0.329 | 3.321 × 10⁻⁰⁵ | 4.541 × 10⁻⁰³ | 4.103 × 10⁻⁰¹ |
| *MEN1* | -0.187 | -0.290 | -0.080 | 7.024 × 10⁻⁰⁴ | 2.078 × 10⁻⁰² | 4.107 × 10⁻⁰¹ |
| *SLC25A33* | -0.182 | -0.285 | -0.075 | 9.956 × 10⁻⁰⁴ | 2.499 × 10⁻⁰² | 4.118 × 10⁻⁰¹ |
| *GGT7* | -0.186 | -0.289 | -0.079 | 7.484 × 10⁻⁰⁴ | 2.163 × 10⁻⁰² | 4.132 × 10⁻⁰¹ |
| *PHF3* | -0.172 | -0.276 | -0.064 | 1.866 × 10⁻⁰³ | 3.589 × 10⁻⁰² | 4.134 × 10⁻⁰¹ |
| *RBM16* | -0.187 | -0.290 | -0.080 | 6.958 × 10⁻⁰⁴ | 2.069 × 10⁻⁰² | 4.149 × 10⁻⁰¹ |
| *ESX1* | -0.214 | -0.316 | -0.108 | 9.985 × 10⁻⁰⁵ | 8.672 × 10⁻⁰³ | 4.150 × 10⁻⁰¹ |
| *C19orf41* | -0.209 | -0.311 | -0.103 | 1.462 × 10⁻⁰⁴ | 1.055 × 10⁻⁰² | 4.154 × 10⁻⁰¹ |
| *NCRNA00164* | -0.230 | -0.330 | -0.124 | 2.980 × 10⁻⁰⁵ | 4.370 × 10⁻⁰³ | 4.168 × 10⁻⁰¹ |
| *NUP153* | -0.167 | -0.271 | -0.059 | 2.525 × 10⁻⁰³ | 4.340 × 10⁻⁰² | 4.174 × 10⁻⁰¹ |
| *X..14* | -0.209 | -0.311 | -0.102 | 1.532 × 10⁻⁰⁴ | 1.092 × 10⁻⁰² | 4.193 × 10⁻⁰¹ |
| *MYL1* | -0.197 | -0.299 | -0.090 | 3.635 × 10⁻⁰⁴ | 1.476 × 10⁻⁰² | 4.200 × 10⁻⁰¹ |
| *GCM2* | -0.273 | -0.371 | -0.169 | 6.149 × 10⁻⁰⁷ | 4.608 × 10⁻⁰⁴ | 4.204 × 10⁻⁰¹ |
| *GDAP1L1* | -0.196 | -0.298 | -0.089 | 3.919 × 10⁻⁰⁴ | 1.528 × 10⁻⁰² | 4.210 × 10⁻⁰¹ |
| *EML4* | -0.178 | -0.282 | -0.071 | 1.253 × 10⁻⁰³ | 2.865 × 10⁻⁰² | 4.224 × 10⁻⁰¹ |
| *PTBP2* | -0.187 | -0.290 | -0.080 | 6.973 × 10⁻⁰⁴ | 2.069 × 10⁻⁰² | 4.227 × 10⁻⁰¹ |
| *LOC400752* | -0.174 | -0.277 | -0.066 | 1.693 × 10⁻⁰³ | 3.386 × 10⁻⁰² | 4.238 × 10⁻⁰¹ |
| *TPM4* | 0.168 | 0.061 | 0.272 | 2.359 × 10⁻⁰³ | 4.195 × 10⁻⁰² | 4.261 × 10⁻⁰¹ |
| *TPPP2* | -0.194 | -0.296 | -0.087 | 4.525 × 10⁻⁰⁴ | 1.664 × 10⁻⁰² | 4.267 × 10⁻⁰¹ |
| *CSNK1D* | -0.175 | -0.279 | -0.068 | 1.525 × 10⁻⁰³ | 3.177 × 10⁻⁰² | 4.272 × 10⁻⁰¹ |
| *ACTRT1* | -0.217 | -0.318 | -0.110 | 8.421 × 10⁻⁰⁵ | 7.817 × 10⁻⁰³ | 4.273 × 10⁻⁰¹ |
| *KCNH2* | -0.167 | -0.271 | -0.059 | 2.599 × 10⁻⁰³ | 4.419 × 10⁻⁰² | 4.273 × 10⁻⁰¹ |
| *SLC38A6* | 0.173 | 0.065 | 0.276 | 1.821 × 10⁻⁰³ | 3.536 × 10⁻⁰² | 4.274 × 10⁻⁰¹ |
| *CCHCR1* | -0.203 | -0.305 | -0.096 | 2.382 × 10⁻⁰⁴ | 1.334 × 10⁻⁰² | 4.288 × 10⁻⁰¹ |
| *SIM1* | -0.244 | -0.344 | -0.139 | 8.737 × 10⁻⁰⁶ | 2.319 × 10⁻⁰³ | 4.291 × 10⁻⁰¹ |
| *CHRNA7* | -0.168 | -0.272 | -0.060 | 2.464 × 10⁻⁰³ | 4.289 × 10⁻⁰² | 4.295 × 10⁻⁰¹ |
| *KCNA2* | -0.186 | -0.289 | -0.078 | 7.868 × 10⁻⁰⁴ | 2.210 × 10⁻⁰² | 4.299 × 10⁻⁰¹ |
| *PMCHL1* | -0.191 | -0.294 | -0.084 | 5.349 × 10⁻⁰⁴ | 1.783 × 10⁻⁰² | 4.321 × 10⁻⁰¹ |
| *FOXJ3* | -0.193 | -0.296 | -0.086 | 4.741 × 10⁻⁰⁴ | 1.690 × 10⁻⁰² | 4.323 × 10⁻⁰¹ |
| *SEL1L3* | 0.177 | 0.069 | 0.280 | 1.384 × 10⁻⁰³ | 3.052 × 10⁻⁰² | 4.326 × 10⁻⁰¹ |
| *TMEM151B* | -0.193 | -0.296 | -0.086 | 4.743 × 10⁻⁰⁴ | 1.690 × 10⁻⁰² | 4.356 × 10⁻⁰¹ |
| *SLC37A4* | -0.226 | -0.327 | -0.120 | 4.008 × 10⁻⁰⁵ | 5.133 × 10⁻⁰³ | 4.365 × 10⁻⁰¹ |
| *KCNC1* | -0.176 | -0.280 | -0.069 | 1.433 × 10⁻⁰³ | 3.078 × 10⁻⁰² | 4.385 × 10⁻⁰¹ |
| *TCHP* | -0.175 | -0.279 | -0.067 | 1.566 × 10⁻⁰³ | 3.225 × 10⁻⁰² | 4.387 × 10⁻⁰¹ |
| *FAM123A* | -0.187 | -0.290 | -0.079 | 7.309 × 10⁻⁰⁴ | 2.129 × 10⁻⁰² | 4.416 × 10⁻⁰¹ |
| *SLC7A13* | -0.165 | -0.269 | -0.057 | 2.974 × 10⁻⁰³ | 4.781 × 10⁻⁰² | 4.426 × 10⁻⁰¹ |
| *UGT3A2* | -0.247 | -0.347 | -0.142 | 6.841 × 10⁻⁰⁶ | 2.006 × 10⁻⁰³ | 4.428 × 10⁻⁰¹ |
| *TPTE2P3* | -0.164 | -0.268 | -0.056 | 3.127 × 10⁻⁰³ | 4.924 × 10⁻⁰² | 4.429 × 10⁻⁰¹ |
| *MYO5A* | 0.179 | 0.072 | 0.283 | 1.180 × 10⁻⁰³ | 2.759 × 10⁻⁰² | 4.480 × 10⁻⁰¹ |
| *GNAL* | 0.167 | 0.059 | 0.271 | 2.625 × 10⁻⁰³ | 4.448 × 10⁻⁰² | 4.481 × 10⁻⁰¹ |
| *LOC285627* | -0.170 | -0.274 | -0.062 | 2.100 × 10⁻⁰³ | 3.873 × 10⁻⁰² | 4.497 × 10⁻⁰¹ |
| *DEFB129* | -0.172 | -0.276 | -0.065 | 1.832 × 10⁻⁰³ | 3.554 × 10⁻⁰² | 4.506 × 10⁻⁰¹ |
| *NOS2* | -0.188 | -0.291 | -0.081 | 6.720 × 10⁻⁰⁴ | 2.039 × 10⁻⁰² | 4.515 × 10⁻⁰¹ |
| *MRPS18B* | -0.178 | -0.281 | -0.070 | 1.319 × 10⁻⁰³ | 2.951 × 10⁻⁰² | 4.518 × 10⁻⁰¹ |
| *ZNF643* | -0.181 | -0.285 | -0.074 | 1.038 × 10⁻⁰³ | 2.562 × 10⁻⁰² | 4.525 × 10⁻⁰¹ |
| *ABCC3* | 0.203 | 0.097 | 0.306 | 2.267 × 10⁻⁰⁴ | 1.315 × 10⁻⁰² | 4.528 × 10⁻⁰¹ |
| *LNP1* | -0.164 | -0.268 | -0.056 | 3.149 × 10⁻⁰³ | 4.943 × 10⁻⁰² | 4.546 × 10⁻⁰¹ |
| *ARPC5* | 0.171 | 0.063 | 0.274 | 2.056 × 10⁻⁰³ | 3.837 × 10⁻⁰² | 4.547 × 10⁻⁰¹ |
| *TIGD4* | -0.207 | -0.309 | -0.100 | 1.794 × 10⁻⁰⁴ | 1.167 × 10⁻⁰² | 4.553 × 10⁻⁰¹ |
| *RBM14* | -0.168 | -0.272 | -0.060 | 2.423 × 10⁻⁰³ | 4.260 × 10⁻⁰² | 4.567 × 10⁻⁰¹ |
| *FBN3* | -0.190 | -0.293 | -0.083 | 5.699 × 10⁻⁰⁴ | 1.848 × 10⁻⁰² | 4.572 × 10⁻⁰¹ |
| *SOX3* | -0.193 | -0.295 | -0.086 | 4.856 × 10⁻⁰⁴ | 1.712 × 10⁻⁰² | 4.589 × 10⁻⁰¹ |
| *ING5* | -0.166 | -0.270 | -0.058 | 2.775 × 10⁻⁰³ | 4.602 × 10⁻⁰² | 4.609 × 10⁻⁰¹ |
| *GPR26* | -0.190 | -0.293 | -0.083 | 5.694 × 10⁻⁰⁴ | 1.848 × 10⁻⁰² | 4.628 × 10⁻⁰¹ |
| *C9orf71* | -0.166 | -0.270 | -0.058 | 2.732 × 10⁻⁰³ | 4.561 × 10⁻⁰² | 4.654 × 10⁻⁰¹ |
| *TCEAL5* | -0.165 | -0.269 | -0.057 | 2.824 × 10⁻⁰³ | 4.639 × 10⁻⁰² | 4.654 × 10⁻⁰¹ |
| *KIAA1267* | -0.175 | -0.279 | -0.067 | 1.554 × 10⁻⁰³ | 3.206 × 10⁻⁰² | 4.658 × 10⁻⁰¹ |
| *CBX8* | -0.189 | -0.292 | -0.082 | 6.359 × 10⁻⁰⁴ | 1.962 × 10⁻⁰² | 4.686 × 10⁻⁰¹ |
| *LOC100134368* | -0.166 | -0.270 | -0.058 | 2.800 × 10⁻⁰³ | 4.611 × 10⁻⁰² | 4.690 × 10⁻⁰¹ |
| *ACTL7A* | -0.218 | -0.320 | -0.112 | 7.487 × 10⁻⁰⁵ | 7.392 × 10⁻⁰³ | 4.712 × 10⁻⁰¹ |
| *KRCC1* | 0.219 | 0.113 | 0.321 | 6.798 × 10⁻⁰⁵ | 7.131 × 10⁻⁰³ | 4.715 × 10⁻⁰¹ |
| *IQCF3* | -0.170 | -0.274 | -0.063 | 2.085 × 10⁻⁰³ | 3.855 × 10⁻⁰² | 4.717 × 10⁻⁰¹ |
| *FAM194B* | -0.246 | -0.345 | -0.140 | 7.798 × 10⁻⁰⁶ | 2.162 × 10⁻⁰³ | 4.743 × 10⁻⁰¹ |
| *NFKBIL1* | -0.201 | -0.303 | -0.094 | 2.724 × 10⁻⁰⁴ | 1.433 × 10⁻⁰² | 4.748 × 10⁻⁰¹ |
| *ZNF821* | -0.176 | -0.279 | -0.068 | 1.504 × 10⁻⁰³ | 3.150 × 10⁻⁰² | 4.768 × 10⁻⁰¹ |
| *OR52A4* | -0.206 | -0.308 | -0.100 | 1.836 × 10⁻⁰⁴ | 1.179 × 10⁻⁰² | 4.778 × 10⁻⁰¹ |
| *PKHD1* | -0.165 | -0.269 | -0.057 | 2.857 × 10⁻⁰³ | 4.683 × 10⁻⁰² | 4.783 × 10⁻⁰¹ |
| *MSH4* | -0.167 | -0.271 | -0.059 | 2.595 × 10⁻⁰³ | 4.416 × 10⁻⁰² | 4.794 × 10⁻⁰¹ |
| *GSG1L* | -0.191 | -0.294 | -0.084 | 5.478 × 10⁻⁰⁴ | 1.805 × 10⁻⁰² | 4.801 × 10⁻⁰¹ |
| *PELP1* | -0.196 | -0.299 | -0.089 | 3.849 × 10⁻⁰⁴ | 1.520 × 10⁻⁰² | 4.828 × 10⁻⁰¹ |
| *RNF186* | -0.246 | -0.345 | -0.140 | 7.718 × 10⁻⁰⁶ | 2.162 × 10⁻⁰³ | 4.849 × 10⁻⁰¹ |
| *HNRNPA1L2* | -0.180 | -0.283 | -0.072 | 1.164 × 10⁻⁰³ | 2.742 × 10⁻⁰² | 4.850 × 10⁻⁰¹ |
| *DIS3L2* | -0.184 | -0.287 | -0.077 | 8.726 × 10⁻⁰⁴ | 2.305 × 10⁻⁰² | 4.852 × 10⁻⁰¹ |
| *TADA2A* | -0.200 | -0.303 | -0.093 | 2.832 × 10⁻⁰⁴ | 1.433 × 10⁻⁰² | 4.863 × 10⁻⁰¹ |
| *CHKA* | -0.194 | -0.296 | -0.087 | 4.515 × 10⁻⁰⁴ | 1.664 × 10⁻⁰² | 4.871 × 10⁻⁰¹ |
| *LOC728024* | -0.172 | -0.276 | -0.065 | 1.853 × 10⁻⁰³ | 3.575 × 10⁻⁰² | 4.875 × 10⁻⁰¹ |
| *KRTAP5.5* | -0.208 | -0.310 | -0.101 | 1.613 × 10⁻⁰⁴ | 1.129 × 10⁻⁰² | 4.875 × 10⁻⁰¹ |
| *LOC100130148* | -0.261 | -0.359 | -0.156 | 2.002 × 10⁻⁰⁶ | 8.792 × 10⁻⁰⁴ | 4.884 × 10⁻⁰¹ |
| *AMBN* | -0.208 | -0.310 | -0.101 | 1.648 × 10⁻⁰⁴ | 1.130 × 10⁻⁰² | 4.945 × 10⁻⁰¹ |
| *OR13G1* | -0.180 | -0.283 | -0.072 | 1.155 × 10⁻⁰³ | 2.736 × 10⁻⁰² | 4.947 × 10⁻⁰¹ |
| *EVX2* | -0.260 | -0.358 | -0.155 | 2.162 × 10⁻⁰⁶ | 8.792 × 10⁻⁰⁴ | 4.950 × 10⁻⁰¹ |
| *CPPED1* | 0.180 | 0.072 | 0.283 | 1.157 × 10⁻⁰³ | 2.736 × 10⁻⁰² | 4.978 × 10⁻⁰¹ |
| *AGXT2* | -0.244 | -0.344 | -0.139 | 8.825 × 10⁻⁰⁶ | 2.319 × 10⁻⁰³ | 4.986 × 10⁻⁰¹ |
| *SBK1* | -0.173 | -0.277 | -0.065 | 1.766 × 10⁻⁰³ | 3.467 × 10⁻⁰² | 5.034 × 10⁻⁰¹ |
| *RTL1* | -0.173 | -0.277 | -0.065 | 1.783 × 10⁻⁰³ | 3.495 × 10⁻⁰² | 5.038 × 10⁻⁰¹ |
| *PDXP* | -0.184 | -0.287 | -0.077 | 8.786 × 10⁻⁰⁴ | 2.312 × 10⁻⁰² | 5.067 × 10⁻⁰¹ |
| *UNK* | -0.176 | -0.280 | -0.069 | 1.448 × 10⁻⁰³ | 3.095 × 10⁻⁰² | 5.072 × 10⁻⁰¹ |
| *LRRC8E* | 0.180 | 0.073 | 0.284 | 1.111 × 10⁻⁰³ | 2.663 × 10⁻⁰² | 5.078 × 10⁻⁰¹ |
| *PYGL* | 0.185 | 0.077 | 0.288 | 8.358 × 10⁻⁰⁴ | 2.272 × 10⁻⁰² | 5.085 × 10⁻⁰¹ |
| *PEX5* | -0.176 | -0.280 | -0.068 | 1.474 × 10⁻⁰³ | 3.122 × 10⁻⁰² | 5.121 × 10⁻⁰¹ |
| *GCNT7* | -0.234 | -0.334 | -0.128 | 2.134 × 10⁻⁰⁵ | 3.514 × 10⁻⁰³ | 5.132 × 10⁻⁰¹ |
| *RBM15B* | -0.189 | -0.292 | -0.082 | 6.079 × 10⁻⁰⁴ | 1.916 × 10⁻⁰² | 5.147 × 10⁻⁰¹ |
| *FAM9C* | -0.241 | -0.341 | -0.136 | 1.148 × 10⁻⁰⁵ | 2.478 × 10⁻⁰³ | 5.159 × 10⁻⁰¹ |
| *UBL4B* | -0.192 | -0.295 | -0.085 | 5.055 × 10⁻⁰⁴ | 1.751 × 10⁻⁰² | 5.168 × 10⁻⁰¹ |
| *OR7C2* | -0.191 | -0.294 | -0.084 | 5.429 × 10⁻⁰⁴ | 1.795 × 10⁻⁰² | 5.170 × 10⁻⁰¹ |
| *GCAT* | -0.193 | -0.296 | -0.086 | 4.823 × 10⁻⁰⁴ | 1.706 × 10⁻⁰² | 5.173 × 10⁻⁰¹ |
| *TIGD3* | -0.186 | -0.289 | -0.079 | 7.593 × 10⁻⁰⁴ | 2.186 × 10⁻⁰² | 5.184 × 10⁻⁰¹ |
| *OR3A1* | -0.180 | -0.283 | -0.072 | 1.156 × 10⁻⁰³ | 2.736 × 10⁻⁰² | 5.185 × 10⁻⁰¹ |
| *HFM1* | -0.213 | -0.314 | -0.106 | 1.154 × 10⁻⁰⁴ | 9.232 × 10⁻⁰³ | 5.206 × 10⁻⁰¹ |
| *PRKCSH* | -0.184 | -0.287 | -0.076 | 8.949 × 10⁻⁰⁴ | 2.331 × 10⁻⁰² | 5.210 × 10⁻⁰¹ |
| *TUBB* | -0.186 | -0.289 | -0.079 | 7.674 × 10⁻⁰⁴ | 2.200 × 10⁻⁰² | 5.221 × 10⁻⁰¹ |
| *USH2A* | -0.164 | -0.268 | -0.056 | 3.018 × 10⁻⁰³ | 4.816 × 10⁻⁰² | 5.229 × 10⁻⁰¹ |
| *MARCH11* | -0.171 | -0.274 | -0.063 | 2.064 × 10⁻⁰³ | 3.837 × 10⁻⁰² | 5.239 × 10⁻⁰¹ |
| *OR3A2* | -0.202 | -0.304 | -0.095 | 2.491 × 10⁻⁰⁴ | 1.366 × 10⁻⁰² | 5.243 × 10⁻⁰¹ |
| *ABLIM2* | 0.165 | 0.057 | 0.269 | 2.918 × 10⁻⁰³ | 4.727 × 10⁻⁰² | 5.249 × 10⁻⁰¹ |
| *ARPP21* | -0.184 | -0.287 | -0.076 | 8.966 × 10⁻⁰⁴ | 2.332 × 10⁻⁰² | 5.257 × 10⁻⁰¹ |
| *MAFB* | 0.170 | 0.063 | 0.274 | 2.077 × 10⁻⁰³ | 3.853 × 10⁻⁰² | 5.335 × 10⁻⁰¹ |
| *OR2AK2* | -0.206 | -0.308 | -0.099 | 1.936 × 10⁻⁰⁴ | 1.195 × 10⁻⁰² | 5.358 × 10⁻⁰¹ |
| *U2AF2* | -0.177 | -0.280 | -0.069 | 1.403 × 10⁻⁰³ | 3.063 × 10⁻⁰² | 5.362 × 10⁻⁰¹ |
| *GTF3C2* | -0.182 | -0.286 | -0.075 | 9.817 × 10⁻⁰⁴ | 2.483 × 10⁻⁰² | 5.368 × 10⁻⁰¹ |
| *BCORL1* | -0.189 | -0.292 | -0.082 | 6.346 × 10⁻⁰⁴ | 1.960 × 10⁻⁰² | 5.370 × 10⁻⁰¹ |
| *BACH1* | 0.182 | 0.075 | 0.285 | 9.920 × 10⁻⁰⁴ | 2.499 × 10⁻⁰² | 5.402 × 10⁻⁰¹ |
| *FAM186B* | -0.177 | -0.280 | -0.069 | 1.408 × 10⁻⁰³ | 3.066 × 10⁻⁰² | 5.404 × 10⁻⁰¹ |
| *MYH13* | -0.168 | -0.272 | -0.060 | 2.484 × 10⁻⁰³ | 4.289 × 10⁻⁰² | 5.409 × 10⁻⁰¹ |
| *CDH22* | -0.219 | -0.320 | -0.112 | 7.211 × 10⁻⁰⁵ | 7.296 × 10⁻⁰³ | 5.410 × 10⁻⁰¹ |
| *AGBL5* | -0.187 | -0.290 | -0.080 | 6.966 × 10⁻⁰⁴ | 2.069 × 10⁻⁰² | 5.421 × 10⁻⁰¹ |
| *S100A6* | 0.180 | 0.073 | 0.284 | 1.113 × 10⁻⁰³ | 2.666 × 10⁻⁰² | 5.445 × 10⁻⁰¹ |
| *PLAC1L* | -0.224 | -0.325 | -0.118 | 4.586 × 10⁻⁰⁵ | 5.521 × 10⁻⁰³ | 5.452 × 10⁻⁰¹ |
| *MS4A12* | -0.175 | -0.279 | -0.068 | 1.545 × 10⁻⁰³ | 3.196 × 10⁻⁰² | 5.494 × 10⁻⁰¹ |
| *GDF9* | -0.185 | -0.289 | -0.078 | 7.972 × 10⁻⁰⁴ | 2.224 × 10⁻⁰² | 5.498 × 10⁻⁰¹ |
| *TAGLN2* | 0.168 | 0.060 | 0.272 | 2.439 × 10⁻⁰³ | 4.269 × 10⁻⁰² | 5.500 × 10⁻⁰¹ |
| *POLR3B* | -0.214 | -0.315 | -0.107 | 1.061 × 10⁻⁰⁴ | 8.869 × 10⁻⁰³ | 5.507 × 10⁻⁰¹ |
| *SNTG1* | -0.187 | -0.290 | -0.079 | 7.392 × 10⁻⁰⁴ | 2.148 × 10⁻⁰² | 5.511 × 10⁻⁰¹ |
| *TAF8* | -0.163 | -0.268 | -0.055 | 3.191 × 10⁻⁰³ | 4.984 × 10⁻⁰² | 5.513 × 10⁻⁰¹ |
| *FRG2B* | -0.170 | -0.273 | -0.062 | 2.199 × 10⁻⁰³ | 3.994 × 10⁻⁰² | 5.540 × 10⁻⁰¹ |
| *LOC84931* | -0.179 | -0.282 | -0.071 | 1.214 × 10⁻⁰³ | 2.804 × 10⁻⁰² | 5.552 × 10⁻⁰¹ |
| *RFX6* | -0.177 | -0.280 | -0.069 | 1.416 × 10⁻⁰³ | 3.071 × 10⁻⁰² | 5.564 × 10⁻⁰¹ |
| *SPINT3* | -0.194 | -0.297 | -0.087 | 4.460 × 10⁻⁰⁴ | 1.656 × 10⁻⁰² | 5.572 × 10⁻⁰¹ |
| *NPY2R* | -0.228 | -0.329 | -0.123 | 3.299 × 10⁻⁰⁵ | 4.541 × 10⁻⁰³ | 5.588 × 10⁻⁰¹ |
| *TMSB15A* | -0.188 | -0.291 | -0.081 | 6.581 × 10⁻⁰⁴ | 1.999 × 10⁻⁰² | 5.602 × 10⁻⁰¹ |
| *CTCFL* | -0.166 | -0.270 | -0.058 | 2.755 × 10⁻⁰³ | 4.579 × 10⁻⁰² | 5.607 × 10⁻⁰¹ |
| *NRXN1* | -0.187 | -0.290 | -0.080 | 7.142 × 10⁻⁰⁴ | 2.104 × 10⁻⁰² | 5.608 × 10⁻⁰¹ |
| *RSAD1* | -0.187 | -0.290 | -0.080 | 7.053 × 10⁻⁰⁴ | 2.081 × 10⁻⁰² | 5.615 × 10⁻⁰¹ |
| *DPYSL5* | -0.187 | -0.290 | -0.080 | 7.035 × 10⁻⁰⁴ | 2.078 × 10⁻⁰² | 5.618 × 10⁻⁰¹ |
| *INTS4* | -0.164 | -0.268 | -0.056 | 3.066 × 10⁻⁰³ | 4.855 × 10⁻⁰² | 5.620 × 10⁻⁰¹ |
| *GPN3* | -0.182 | -0.285 | -0.074 | 1.014 × 10⁻⁰³ | 2.531 × 10⁻⁰² | 5.660 × 10⁻⁰¹ |
| *MYT1* | -0.206 | -0.308 | -0.099 | 1.857 × 10⁻⁰⁴ | 1.185 × 10⁻⁰² | 5.668 × 10⁻⁰¹ |
| *HSP90AB4P* | -0.163 | -0.268 | -0.055 | 3.183 × 10⁻⁰³ | 4.982 × 10⁻⁰² | 5.709 × 10⁻⁰¹ |
| *CNTNAP5* | -0.171 | -0.275 | -0.063 | 2.049 × 10⁻⁰³ | 3.828 × 10⁻⁰² | 5.722 × 10⁻⁰¹ |
| *OR5H2* | -0.200 | -0.303 | -0.093 | 2.885 × 10⁻⁰⁴ | 1.433 × 10⁻⁰² | 5.723 × 10⁻⁰¹ |
| *PRAMEF17* | -0.177 | -0.281 | -0.069 | 1.371 × 10⁻⁰³ | 3.038 × 10⁻⁰² | 5.777 × 10⁻⁰¹ |
| *CASC3* | -0.188 | -0.291 | -0.081 | 6.519 × 10⁻⁰⁴ | 1.990 × 10⁻⁰² | 5.787 × 10⁻⁰¹ |
| *DHRS13* | -0.221 | -0.322 | -0.115 | 5.925 × 10⁻⁰⁵ | 6.480 × 10⁻⁰³ | 5.789 × 10⁻⁰¹ |
| *OR56B4* | -0.195 | -0.297 | -0.088 | 4.211 × 10⁻⁰⁴ | 1.593 × 10⁻⁰² | 5.816 × 10⁻⁰¹ |
| *MAGEC3* | -0.231 | -0.332 | -0.125 | 2.663 × 10⁻⁰⁵ | 4.082 × 10⁻⁰³ | 5.822 × 10⁻⁰¹ |
| *MOGAT3* | -0.170 | -0.274 | -0.062 | 2.131 × 10⁻⁰³ | 3.906 × 10⁻⁰² | 5.825 × 10⁻⁰¹ |
| *GBX1* | -0.210 | -0.312 | -0.103 | 1.411 × 10⁻⁰⁴ | 1.035 × 10⁻⁰² | 5.834 × 10⁻⁰¹ |
| *VSX1* | -0.171 | -0.275 | -0.064 | 1.970 × 10⁻⁰³ | 3.732 × 10⁻⁰² | 5.869 × 10⁻⁰¹ |
| *DDX42* | -0.191 | -0.294 | -0.084 | 5.309 × 10⁻⁰⁴ | 1.782 × 10⁻⁰² | 5.922 × 10⁻⁰¹ |
| *GOLGA7B* | 0.178 | 0.070 | 0.282 | 1.286 × 10⁻⁰³ | 2.914 × 10⁻⁰² | 5.924 × 10⁻⁰¹ |
| *KRT40* | -0.199 | -0.301 | -0.092 | 3.155 × 10⁻⁰⁴ | 1.433 × 10⁻⁰² | 5.944 × 10⁻⁰¹ |
| *AAAS* | -0.184 | -0.287 | -0.077 | 8.657 × 10⁻⁰⁴ | 2.301 × 10⁻⁰² | 5.947 × 10⁻⁰¹ |
| *RDBP* | -0.210 | -0.312 | -0.103 | 1.397 × 10⁻⁰⁴ | 1.032 × 10⁻⁰² | 5.961 × 10⁻⁰¹ |
| *OR56A5* | -0.233 | -0.334 | -0.127 | 2.267 × 10⁻⁰⁵ | 3.584 × 10⁻⁰³ | 5.989 × 10⁻⁰¹ |
| *TYR* | -0.167 | -0.271 | -0.059 | 2.608 × 10⁻⁰³ | 4.431 × 10⁻⁰² | 6.022 × 10⁻⁰¹ |
| *OR6C65* | -0.184 | -0.287 | -0.077 | 8.748 × 10⁻⁰⁴ | 2.305 × 10⁻⁰² | 6.026 × 10⁻⁰¹ |
| *ZMYM3* | -0.203 | -0.306 | -0.097 | 2.265 × 10⁻⁰⁴ | 1.315 × 10⁻⁰² | 6.028 × 10⁻⁰¹ |
| *REXO1L1* | -0.174 | -0.278 | -0.066 | 1.675 × 10⁻⁰³ | 3.373 × 10⁻⁰² | 6.053 × 10⁻⁰¹ |
| *PLIN3* | 0.172 | 0.065 | 0.276 | 1.853 × 10⁻⁰³ | 3.575 × 10⁻⁰² | 6.071 × 10⁻⁰¹ |
| *EIF2B4* | -0.229 | -0.330 | -0.123 | 3.159 × 10⁻⁰⁵ | 4.439 × 10⁻⁰³ | 6.088 × 10⁻⁰¹ |
| *ADAMTS19* | -0.243 | -0.343 | -0.137 | 1.001 × 10⁻⁰⁵ | 2.454 × 10⁻⁰³ | 6.089 × 10⁻⁰¹ |
| *SVOP* | -0.168 | -0.272 | -0.060 | 2.448 × 10⁻⁰³ | 4.274 × 10⁻⁰² | 6.116 × 10⁻⁰¹ |
| *SELV* | -0.214 | -0.315 | -0.107 | 1.044 × 10⁻⁰⁴ | 8.766 × 10⁻⁰³ | 6.119 × 10⁻⁰¹ |
| *TAS2R16* | -0.192 | -0.295 | -0.085 | 4.955 × 10⁻⁰⁴ | 1.726 × 10⁻⁰² | 6.136 × 10⁻⁰¹ |
| *UBA1* | -0.177 | -0.280 | -0.069 | 1.415 × 10⁻⁰³ | 3.071 × 10⁻⁰² | 6.141 × 10⁻⁰¹ |
| *LIG3* | -0.174 | -0.277 | -0.066 | 1.716 × 10⁻⁰³ | 3.421 × 10⁻⁰² | 6.158 × 10⁻⁰¹ |
| *TBX22* | -0.264 | -0.363 | -0.160 | 1.424 × 10⁻⁰⁶ | 7.202 × 10⁻⁰⁴ | 6.160 × 10⁻⁰¹ |
| *KIF1A* | -0.186 | -0.289 | -0.078 | 7.838 × 10⁻⁰⁴ | 2.210 × 10⁻⁰² | 6.163 × 10⁻⁰¹ |
| *NKX6.2* | -0.201 | -0.303 | -0.094 | 2.806 × 10⁻⁰⁴ | 1.433 × 10⁻⁰² | 6.202 × 10⁻⁰¹ |
| *IMP5* | -0.184 | -0.288 | -0.077 | 8.484 × 10⁻⁰⁴ | 2.280 × 10⁻⁰² | 6.202 × 10⁻⁰¹ |
| *OAZ1* | 0.235 | 0.130 | 0.336 | 1.862 × 10⁻⁰⁵ | 3.305 × 10⁻⁰³ | 6.204 × 10⁻⁰¹ |
| *GPKOW* | -0.193 | -0.296 | -0.086 | 4.745 × 10⁻⁰⁴ | 1.690 × 10⁻⁰² | 6.206 × 10⁻⁰¹ |
| *SF1* | -0.217 | -0.319 | -0.111 | 8.167 × 10⁻⁰⁵ | 7.680 × 10⁻⁰³ | 6.208 × 10⁻⁰¹ |
| *TGIF2* | -0.219 | -0.320 | -0.113 | 7.001 × 10⁻⁰⁵ | 7.131 × 10⁻⁰³ | 6.218 × 10⁻⁰¹ |
| *WBP11* | -0.239 | -0.340 | -0.134 | 1.323 × 10⁻⁰⁵ | 2.651 × 10⁻⁰³ | 6.230 × 10⁻⁰¹ |
| *LOC642597* | -0.192 | -0.295 | -0.085 | 5.201 × 10⁻⁰⁴ | 1.768 × 10⁻⁰² | 6.242 × 10⁻⁰¹ |
| *KRTAP3.3* | -0.170 | -0.274 | -0.063 | 2.081 × 10⁻⁰³ | 3.854 × 10⁻⁰² | 6.253 × 10⁻⁰¹ |
| *OR52N1* | -0.277 | -0.375 | -0.173 | 4.008 × 10⁻⁰⁷ | 3.332 × 10⁻⁰⁴ | 6.257 × 10⁻⁰¹ |
| *GDF1* | -0.185 | -0.288 | -0.078 | 8.162 × 10⁻⁰⁴ | 2.247 × 10⁻⁰² | 6.268 × 10⁻⁰¹ |
| *SLC7A3* | -0.218 | -0.319 | -0.112 | 7.588 × 10⁻⁰⁵ | 7.392 × 10⁻⁰³ | 6.293 × 10⁻⁰¹ |
| *TMEM165* | 0.183 | 0.076 | 0.286 | 9.274 × 10⁻⁰⁴ | 2.394 × 10⁻⁰² | 6.302 × 10⁻⁰¹ |
| *DBN1* | -0.167 | -0.271 | -0.059 | 2.563 × 10⁻⁰³ | 4.377 × 10⁻⁰² | 6.312 × 10⁻⁰¹ |
| *VARS2* | -0.173 | -0.276 | -0.065 | 1.810 × 10⁻⁰³ | 3.525 × 10⁻⁰² | 6.336 × 10⁻⁰¹ |
| *UBE2CBP* | -0.232 | -0.333 | -0.127 | 2.411 × 10⁻⁰⁵ | 3.782 × 10⁻⁰³ | 6.339 × 10⁻⁰¹ |
| *DNAJC5G* | -0.271 | -0.369 | -0.167 | 7.296 × 10⁻⁰⁷ | 4.997 × 10⁻⁰⁴ | 6.341 × 10⁻⁰¹ |
| *SLC36A3* | -0.210 | -0.312 | -0.104 | 1.362 × 10⁻⁰⁴ | 1.018 × 10⁻⁰² | 6.353 × 10⁻⁰¹ |
| *HNRNPM* | -0.168 | -0.272 | -0.060 | 2.366 × 10⁻⁰³ | 4.203 × 10⁻⁰² | 6.367 × 10⁻⁰¹ |
| *CLVS2* | -0.196 | -0.299 | -0.089 | 3.846 × 10⁻⁰⁴ | 1.520 × 10⁻⁰² | 6.374 × 10⁻⁰¹ |
| *IGDCC3* | -0.176 | -0.280 | -0.068 | 1.462 × 10⁻⁰³ | 3.115 × 10⁻⁰² | 6.375 × 10⁻⁰¹ |
| *NIPAL2* | 0.192 | 0.084 | 0.294 | 5.234 × 10⁻⁰⁴ | 1.768 × 10⁻⁰² | 6.375 × 10⁻⁰¹ |
| *ZNF362* | -0.175 | -0.278 | -0.067 | 1.593 × 10⁻⁰³ | 3.262 × 10⁻⁰² | 6.377 × 10⁻⁰¹ |
| *OR6V1* | -0.214 | -0.315 | -0.107 | 1.068 × 10⁻⁰⁴ | 8.893 × 10⁻⁰³ | 6.379 × 10⁻⁰¹ |
| *RNF5* | -0.195 | -0.298 | -0.088 | 4.189 × 10⁻⁰⁴ | 1.590 × 10⁻⁰² | 6.386 × 10⁻⁰¹ |
| *GPRC6A* | -0.219 | -0.321 | -0.113 | 6.898 × 10⁻⁰⁵ | 7.131 × 10⁻⁰³ | 6.419 × 10⁻⁰¹ |
| *C22orf33* | -0.181 | -0.285 | -0.074 | 1.055 × 10⁻⁰³ | 2.576 × 10⁻⁰² | 6.427 × 10⁻⁰¹ |
| *RCAN1* | 0.165 | 0.057 | 0.269 | 2.860 × 10⁻⁰³ | 4.683 × 10⁻⁰² | 6.454 × 10⁻⁰¹ |
| *XRCC5* | -0.175 | -0.278 | -0.067 | 1.586 × 10⁻⁰³ | 3.253 × 10⁻⁰² | 6.465 × 10⁻⁰¹ |
| *OR1L6* | -0.172 | -0.276 | -0.064 | 1.870 × 10⁻⁰³ | 3.590 × 10⁻⁰² | 6.486 × 10⁻⁰¹ |
| *RSPH6A* | -0.210 | -0.312 | -0.103 | 1.423 × 10⁻⁰⁴ | 1.035 × 10⁻⁰² | 6.487 × 10⁻⁰¹ |
| *C19orf28* | 0.201 | 0.094 | 0.304 | 2.664 × 10⁻⁰⁴ | 1.430 × 10⁻⁰² | 6.491 × 10⁻⁰¹ |
| *PRSS48* | -0.241 | -0.341 | -0.136 | 1.125 × 10⁻⁰⁵ | 2.478 × 10⁻⁰³ | 6.492 × 10⁻⁰¹ |
| *SF3B2* | -0.200 | -0.303 | -0.093 | 2.877 × 10⁻⁰⁴ | 1.433 × 10⁻⁰² | 6.501 × 10⁻⁰¹ |
| *RING1* | -0.208 | -0.310 | -0.101 | 1.659 × 10⁻⁰⁴ | 1.130 × 10⁻⁰² | 6.518 × 10⁻⁰¹ |
| *LOC126536* | -0.227 | -0.328 | -0.121 | 3.818 × 10⁻⁰⁵ | 4.952 × 10⁻⁰³ | 6.520 × 10⁻⁰¹ |
| *FAM113B* | 0.172 | 0.064 | 0.276 | 1.867 × 10⁻⁰³ | 3.589 × 10⁻⁰² | 6.537 × 10⁻⁰¹ |
| *MLNR* | -0.202 | -0.304 | -0.095 | 2.616 × 10⁻⁰⁴ | 1.415 × 10⁻⁰² | 6.549 × 10⁻⁰¹ |
| *RBMXL1* | -0.171 | -0.275 | -0.063 | 2.029 × 10⁻⁰³ | 3.803 × 10⁻⁰² | 6.558 × 10⁻⁰¹ |
| *PPP3CC* | 0.214 | 0.107 | 0.315 | 1.072 × 10⁻⁰⁴ | 8.893 × 10⁻⁰³ | 6.558 × 10⁻⁰¹ |
| *FLJ37201* | -0.165 | -0.269 | -0.057 | 2.854 × 10⁻⁰³ | 4.683 × 10⁻⁰² | 6.559 × 10⁻⁰¹ |
| *P2RX3* | -0.270 | -0.368 | -0.166 | 8.181 × 10⁻⁰⁷ | 5.340 × 10⁻⁰⁴ | 6.574 × 10⁻⁰¹ |
| *CCKBR* | -0.196 | -0.299 | -0.089 | 3.889 × 10⁻⁰⁴ | 1.525 × 10⁻⁰² | 6.574 × 10⁻⁰¹ |
| *FAM123C* | -0.198 | -0.300 | -0.091 | 3.441 × 10⁻⁰⁴ | 1.433 × 10⁻⁰² | 6.576 × 10⁻⁰¹ |
| *ZBTB8B* | -0.166 | -0.270 | -0.058 | 2.774 × 10⁻⁰³ | 4.602 × 10⁻⁰² | 6.579 × 10⁻⁰¹ |
| *ABCG8* | -0.192 | -0.294 | -0.084 | 5.272 × 10⁻⁰⁴ | 1.772 × 10⁻⁰² | 6.597 × 10⁻⁰¹ |
| *OR2AG2* | -0.176 | -0.279 | -0.068 | 1.483 × 10⁻⁰³ | 3.133 × 10⁻⁰² | 6.637 × 10⁻⁰¹ |
| *ZNF232* | -0.219 | -0.320 | -0.113 | 6.955 × 10⁻⁰⁵ | 7.131 × 10⁻⁰³ | 6.645 × 10⁻⁰¹ |
| *BAT2* | -0.205 | -0.307 | -0.099 | 1.971 × 10⁻⁰⁴ | 1.200 × 10⁻⁰² | 6.649 × 10⁻⁰¹ |
| *WDR45L* | -0.194 | -0.297 | -0.087 | 4.505 × 10⁻⁰⁴ | 1.664 × 10⁻⁰² | 6.680 × 10⁻⁰¹ |
| *TPH2* | -0.234 | -0.334 | -0.128 | 2.087 × 10⁻⁰⁵ | 3.514 × 10⁻⁰³ | 6.684 × 10⁻⁰¹ |
| *C7orf52* | -0.197 | -0.300 | -0.090 | 3.551 × 10⁻⁰⁴ | 1.454 × 10⁻⁰² | 6.698 × 10⁻⁰¹ |
| *LAIR2* | 0.176 | 0.068 | 0.279 | 1.495 × 10⁻⁰³ | 3.141 × 10⁻⁰² | 6.702 × 10⁻⁰¹ |
| *MYH6* | -0.186 | -0.289 | -0.079 | 7.730 × 10⁻⁰⁴ | 2.207 × 10⁻⁰² | 6.703 × 10⁻⁰¹ |
| *LOC100133545* | -0.172 | -0.276 | -0.065 | 1.838 × 10⁻⁰³ | 3.555 × 10⁻⁰² | 6.707 × 10⁻⁰¹ |
| *ZMYM2* | -0.200 | -0.302 | -0.093 | 2.943 × 10⁻⁰⁴ | 1.433 × 10⁻⁰² | 6.711 × 10⁻⁰¹ |
| *GPS1* | -0.174 | -0.278 | -0.066 | 1.687 × 10⁻⁰³ | 3.386 × 10⁻⁰² | 6.721 × 10⁻⁰¹ |
| *PCDH8* | -0.177 | -0.280 | -0.069 | 1.395 × 10⁻⁰³ | 3.055 × 10⁻⁰² | 6.721 × 10⁻⁰¹ |
| *SF3A3* | -0.196 | -0.299 | -0.089 | 3.825 × 10⁻⁰⁴ | 1.518 × 10⁻⁰² | 6.750 × 10⁻⁰¹ |
| *LRP6* | -0.200 | -0.303 | -0.094 | 2.823 × 10⁻⁰⁴ | 1.433 × 10⁻⁰² | 6.754 × 10⁻⁰¹ |
| *C21orf7* | 0.215 | 0.109 | 0.317 | 9.342 × 10⁻⁰⁵ | 8.401 × 10⁻⁰³ | 6.765 × 10⁻⁰¹ |
| *SEZ6* | -0.223 | -0.324 | -0.117 | 5.061 × 10⁻⁰⁵ | 5.903 × 10⁻⁰³ | 6.769 × 10⁻⁰¹ |
| *OR5M8* | -0.185 | -0.288 | -0.078 | 8.060 × 10⁻⁰⁴ | 2.231 × 10⁻⁰² | 6.792 × 10⁻⁰¹ |
| *MUC17* | -0.246 | -0.345 | -0.140 | 7.767 × 10⁻⁰⁶ | 2.162 × 10⁻⁰³ | 6.820 × 10⁻⁰¹ |
| *CSNK1A1P* | -0.166 | -0.270 | -0.058 | 2.669 × 10⁻⁰³ | 4.509 × 10⁻⁰² | 6.822 × 10⁻⁰¹ |
| *HACE1* | -0.214 | -0.316 | -0.108 | 1.036 × 10⁻⁰⁴ | 8.766 × 10⁻⁰³ | 6.841 × 10⁻⁰¹ |
| *KCNJ3* | -0.185 | -0.288 | -0.077 | 8.274 × 10⁻⁰⁴ | 2.262 × 10⁻⁰² | 6.851 × 10⁻⁰¹ |
| *ZSWIM2* | -0.225 | -0.326 | -0.119 | 4.456 × 10⁻⁰⁵ | 5.465 × 10⁻⁰³ | 6.857 × 10⁻⁰¹ |
| *FRMD4B* | 0.178 | 0.070 | 0.281 | 1.302 × 10⁻⁰³ | 2.936 × 10⁻⁰² | 6.860 × 10⁻⁰¹ |
| *MATR3* | -0.168 | -0.272 | -0.060 | 2.418 × 10⁻⁰³ | 4.260 × 10⁻⁰² | 6.880 × 10⁻⁰¹ |
| *ARHGAP36* | -0.172 | -0.276 | -0.065 | 1.858 × 10⁻⁰³ | 3.580 × 10⁻⁰² | 6.905 × 10⁻⁰¹ |
| *PTK2B* | 0.205 | 0.098 | 0.307 | 2.089 × 10⁻⁰⁴ | 1.244 × 10⁻⁰² | 6.911 × 10⁻⁰¹ |
| *TRIM10* | -0.180 | -0.283 | -0.072 | 1.163 × 10⁻⁰³ | 2.742 × 10⁻⁰² | 6.934 × 10⁻⁰¹ |
| *PNPLA5* | -0.182 | -0.286 | -0.075 | 9.751 × 10⁻⁰⁴ | 2.470 × 10⁻⁰² | 6.936 × 10⁻⁰¹ |
| *COG1* | -0.178 | -0.281 | -0.070 | 1.321 × 10⁻⁰³ | 2.953 × 10⁻⁰² | 6.936 × 10⁻⁰¹ |
| *PER4* | -0.260 | -0.359 | -0.155 | 2.137 × 10⁻⁰⁶ | 8.792 × 10⁻⁰⁴ | 6.941 × 10⁻⁰¹ |
| *BAT4* | -0.222 | -0.323 | -0.116 | 5.526 × 10⁻⁰⁵ | 6.189 × 10⁻⁰³ | 6.941 × 10⁻⁰¹ |
| *PRDM14* | -0.165 | -0.269 | -0.057 | 2.888 × 10⁻⁰³ | 4.693 × 10⁻⁰² | 6.943 × 10⁻⁰¹ |
| *SLC10A6* | 0.183 | 0.076 | 0.286 | 9.370 × 10⁻⁰⁴ | 2.403 × 10⁻⁰² | 6.951 × 10⁻⁰¹ |
| *AHR* | 0.179 | 0.072 | 0.283 | 1.197 × 10⁻⁰³ | 2.784 × 10⁻⁰² | 6.970 × 10⁻⁰¹ |
| *GANAB* | -0.175 | -0.279 | -0.068 | 1.524 × 10⁻⁰³ | 3.177 × 10⁻⁰² | 6.973 × 10⁻⁰¹ |
| *AQP12A* | -0.165 | -0.270 | -0.058 | 2.814 × 10⁻⁰³ | 4.625 × 10⁻⁰² | 6.978 × 10⁻⁰¹ |
| *KRTAP3.1* | -0.168 | -0.272 | -0.060 | 2.412 × 10⁻⁰³ | 4.258 × 10⁻⁰² | 6.992 × 10⁻⁰¹ |
| *KCNN1* | -0.168 | -0.272 | -0.060 | 2.473 × 10⁻⁰³ | 4.289 × 10⁻⁰² | 6.997 × 10⁻⁰¹ |
| *MPP2* | -0.178 | -0.282 | -0.071 | 1.267 × 10⁻⁰³ | 2.875 × 10⁻⁰² | 7.048 × 10⁻⁰¹ |
| *PAGE2B* | -0.196 | -0.299 | -0.089 | 3.801 × 10⁻⁰⁴ | 1.514 × 10⁻⁰² | 7.066 × 10⁻⁰¹ |
| *TNRC6C* | -0.177 | -0.281 | -0.069 | 1.373 × 10⁻⁰³ | 3.038 × 10⁻⁰² | 7.067 × 10⁻⁰¹ |
| *DKFZP434L187* | -0.244 | -0.344 | -0.139 | 8.779 × 10⁻⁰⁶ | 2.319 × 10⁻⁰³ | 7.073 × 10⁻⁰¹ |
| *NRF1* | -0.168 | -0.272 | -0.060 | 2.385 × 10⁻⁰³ | 4.222 × 10⁻⁰² | 7.107 × 10⁻⁰¹ |
| *ZMYND8* | -0.182 | -0.285 | -0.075 | 9.947 × 10⁻⁰⁴ | 2.499 × 10⁻⁰² | 7.117 × 10⁻⁰¹ |
| *BPHL* | -0.174 | -0.278 | -0.067 | 1.636 × 10⁻⁰³ | 3.322 × 10⁻⁰² | 7.121 × 10⁻⁰¹ |
| *COX7B2* | -0.176 | -0.280 | -0.069 | 1.431 × 10⁻⁰³ | 3.078 × 10⁻⁰² | 7.136 × 10⁻⁰¹ |
| *UBTF* | -0.203 | -0.306 | -0.096 | 2.296 × 10⁻⁰⁴ | 1.319 × 10⁻⁰² | 7.138 × 10⁻⁰¹ |
| *LOC100128977* | -0.181 | -0.284 | -0.073 | 1.089 × 10⁻⁰³ | 2.629 × 10⁻⁰² | 7.162 × 10⁻⁰¹ |
| *LOH12CR2* | -0.183 | -0.286 | -0.075 | 9.432 × 10⁻⁰⁴ | 2.410 × 10⁻⁰² | 7.169 × 10⁻⁰¹ |
| *PHOX2A* | -0.185 | -0.288 | -0.077 | 8.380 × 10⁻⁰⁴ | 2.273 × 10⁻⁰² | 7.188 × 10⁻⁰¹ |
| *RAG2* | -0.172 | -0.276 | -0.064 | 1.887 × 10⁻⁰³ | 3.608 × 10⁻⁰² | 7.195 × 10⁻⁰¹ |
| *PMP2* | -0.184 | -0.287 | -0.076 | 8.900 × 10⁻⁰⁴ | 2.324 × 10⁻⁰² | 7.220 × 10⁻⁰¹ |
| *USP30* | -0.205 | -0.308 | -0.099 | 1.965 × 10⁻⁰⁴ | 1.200 × 10⁻⁰² | 7.225 × 10⁻⁰¹ |
| *HCFC1* | -0.164 | -0.268 | -0.056 | 3.026 × 10⁻⁰³ | 4.822 × 10⁻⁰² | 7.227 × 10⁻⁰¹ |
| *MAGEB18* | -0.229 | -0.330 | -0.124 | 3.044 × 10⁻⁰⁵ | 4.415 × 10⁻⁰³ | 7.228 × 10⁻⁰¹ |
| *ARGFX* | -0.229 | -0.330 | -0.123 | 3.127 × 10⁻⁰⁵ | 4.439 × 10⁻⁰³ | 7.237 × 10⁻⁰¹ |
| *CCDC109B* | 0.164 | 0.056 | 0.268 | 3.037 × 10⁻⁰³ | 4.831 × 10⁻⁰² | 7.241 × 10⁻⁰¹ |
| *MDC1* | -0.271 | -0.369 | -0.167 | 7.408 × 10⁻⁰⁷ | 4.997 × 10⁻⁰⁴ | 7.248 × 10⁻⁰¹ |
| *BRD2* | -0.174 | -0.277 | -0.066 | 1.693 × 10⁻⁰³ | 3.386 × 10⁻⁰² | 7.249 × 10⁻⁰¹ |
| *LOC283999* | -0.167 | -0.271 | -0.059 | 2.548 × 10⁻⁰³ | 4.363 × 10⁻⁰² | 7.253 × 10⁻⁰¹ |
| *C7orf71* | -0.185 | -0.288 | -0.077 | 8.350 × 10⁻⁰⁴ | 2.272 × 10⁻⁰² | 7.285 × 10⁻⁰¹ |
| *VENTXP1* | -0.418 | -0.504 | -0.323 | 4.225 × 10⁻¹⁵ | 8.549 × 10⁻¹¹ | 7.295 × 10⁻⁰¹ |
| *PCGF2* | -0.176 | -0.280 | -0.069 | 1.435 × 10⁻⁰³ | 3.079 × 10⁻⁰² | 7.301 × 10⁻⁰¹ |
| *PSG11* | -0.261 | -0.359 | -0.156 | 1.996 × 10⁻⁰⁶ | 8.792 × 10⁻⁰⁴ | 7.307 × 10⁻⁰¹ |
| *TRIT1* | -0.183 | -0.286 | -0.075 | 9.402 × 10⁻⁰⁴ | 2.405 × 10⁻⁰² | 7.320 × 10⁻⁰¹ |
| *OR11G2* | -0.210 | -0.312 | -0.104 | 1.364 × 10⁻⁰⁴ | 1.018 × 10⁻⁰² | 7.327 × 10⁻⁰¹ |
| *DACH2* | -0.180 | -0.283 | -0.072 | 1.160 × 10⁻⁰³ | 2.739 × 10⁻⁰² | 7.335 × 10⁻⁰¹ |
| *SEPHS1* | -0.164 | -0.268 | -0.056 | 3.013 × 10⁻⁰³ | 4.816 × 10⁻⁰² | 7.355 × 10⁻⁰¹ |
| *C6orf195* | -0.176 | -0.280 | -0.068 | 1.467 × 10⁻⁰³ | 3.117 × 10⁻⁰² | 7.384 × 10⁻⁰¹ |
| *ZNF192* | -0.177 | -0.280 | -0.069 | 1.395 × 10⁻⁰³ | 3.055 × 10⁻⁰² | 7.388 × 10⁻⁰¹ |
| *PFAS* | -0.171 | -0.275 | -0.063 | 2.040 × 10⁻⁰³ | 3.815 × 10⁻⁰² | 7.390 × 10⁻⁰¹ |
| *OR52M1* | -0.165 | -0.269 | -0.057 | 2.886 × 10⁻⁰³ | 4.693 × 10⁻⁰² | 7.393 × 10⁻⁰¹ |
| *HTR1A* | -0.174 | -0.277 | -0.066 | 1.700 × 10⁻⁰³ | 3.395 × 10⁻⁰² | 7.405 × 10⁻⁰¹ |
| *GABRA6* | -0.277 | -0.375 | -0.173 | 4.027 × 10⁻⁰⁷ | 3.332 × 10⁻⁰⁴ | 7.410 × 10⁻⁰¹ |
| *OR56A4* | -0.191 | -0.294 | -0.084 | 5.528 × 10⁻⁰⁴ | 1.816 × 10⁻⁰² | 7.428 × 10⁻⁰¹ |
| *ZNF193* | -0.171 | -0.275 | -0.064 | 1.975 × 10⁻⁰³ | 3.739 × 10⁻⁰² | 7.449 × 10⁻⁰¹ |
| *ZNF479* | -0.189 | -0.292 | -0.082 | 6.149 × 10⁻⁰⁴ | 1.926 × 10⁻⁰² | 7.476 × 10⁻⁰¹ |
| *OR5T2* | -0.196 | -0.299 | -0.089 | 3.870 × 10⁻⁰⁴ | 1.521 × 10⁻⁰² | 7.479 × 10⁻⁰¹ |
| *DNMT3A* | -0.208 | -0.310 | -0.101 | 1.661 × 10⁻⁰⁴ | 1.130 × 10⁻⁰² | 7.480 × 10⁻⁰¹ |
| *CHD6* | -0.169 | -0.273 | -0.062 | 2.216 × 10⁻⁰³ | 4.017 × 10⁻⁰² | 7.513 × 10⁻⁰¹ |
| *TSR2* | -0.196 | -0.298 | -0.089 | 3.951 × 10⁻⁰⁴ | 1.532 × 10⁻⁰² | 7.519 × 10⁻⁰¹ |
| *TRIM15* | -0.169 | -0.273 | -0.061 | 2.296 × 10⁻⁰³ | 4.120 × 10⁻⁰² | 7.521 × 10⁻⁰¹ |
| *LOC222699* | -0.206 | -0.308 | -0.099 | 1.917 × 10⁻⁰⁴ | 1.194 × 10⁻⁰² | 7.524 × 10⁻⁰¹ |
| *C1orf95* | -0.211 | -0.313 | -0.105 | 1.253 × 10⁻⁰⁴ | 9.753 × 10⁻⁰³ | 7.535 × 10⁻⁰¹ |
| *NANOS2* | -0.173 | -0.277 | -0.065 | 1.798 × 10⁻⁰³ | 3.511 × 10⁻⁰² | 7.566 × 10⁻⁰¹ |
| *PIP5K1P1* | -0.192 | -0.295 | -0.085 | 4.937 × 10⁻⁰⁴ | 1.726 × 10⁻⁰² | 7.595 × 10⁻⁰¹ |
| *MOS* | -0.234 | -0.334 | -0.128 | 2.154 × 10⁻⁰⁵ | 3.514 × 10⁻⁰³ | 7.597 × 10⁻⁰¹ |
| *PPM1E* | -0.193 | -0.296 | -0.086 | 4.653 × 10⁻⁰⁴ | 1.690 × 10⁻⁰² | 7.600 × 10⁻⁰¹ |
| *ZIC1* | -0.165 | -0.269 | -0.057 | 2.889 × 10⁻⁰³ | 4.693 × 10⁻⁰² | 7.623 × 10⁻⁰¹ |
| *CD58* | 0.169 | 0.061 | 0.273 | 2.312 × 10⁻⁰³ | 4.125 × 10⁻⁰² | 7.632 × 10⁻⁰¹ |
| *REEP2* | -0.172 | -0.275 | -0.064 | 1.938 × 10⁻⁰³ | 3.686 × 10⁻⁰² | 7.660 × 10⁻⁰¹ |
| *SLC37A2* | 0.201 | 0.094 | 0.303 | 2.752 × 10⁻⁰⁴ | 1.433 × 10⁻⁰² | 7.663 × 10⁻⁰¹ |
| *FAM98B* | -0.170 | -0.274 | -0.062 | 2.113 × 10⁻⁰³ | 3.891 × 10⁻⁰² | 7.666 × 10⁻⁰¹ |
| *GPR32* | -0.203 | -0.305 | -0.096 | 2.303 × 10⁻⁰⁴ | 1.319 × 10⁻⁰² | 7.669 × 10⁻⁰¹ |
| *NANOS3* | -0.173 | -0.276 | -0.065 | 1.807 × 10⁻⁰³ | 3.525 × 10⁻⁰² | 7.681 × 10⁻⁰¹ |
| *DHTKD1* | -0.164 | -0.268 | -0.056 | 3.125 × 10⁻⁰³ | 4.924 × 10⁻⁰² | 7.682 × 10⁻⁰¹ |
| *OR2M1P* | -0.169 | -0.273 | -0.061 | 2.326 × 10⁻⁰³ | 4.146 × 10⁻⁰² | 7.687 × 10⁻⁰¹ |
| *HNRNPA0* | -0.242 | -0.342 | -0.137 | 1.055 × 10⁻⁰⁵ | 2.454 × 10⁻⁰³ | 7.692 × 10⁻⁰¹ |
| *ABCF1* | -0.203 | -0.305 | -0.096 | 2.377 × 10⁻⁰⁴ | 1.334 × 10⁻⁰² | 7.713 × 10⁻⁰¹ |
| *SLC35D3* | -0.170 | -0.274 | -0.063 | 2.096 × 10⁻⁰³ | 3.870 × 10⁻⁰² | 7.719 × 10⁻⁰¹ |
| *CD274* | 0.184 | 0.076 | 0.287 | 9.030 × 10⁻⁰⁴ | 2.346 × 10⁻⁰² | 7.726 × 10⁻⁰¹ |
| *ZNF496* | -0.177 | -0.280 | -0.069 | 1.423 × 10⁻⁰³ | 3.073 × 10⁻⁰² | 7.731 × 10⁻⁰¹ |
| *MGST2* | 0.202 | 0.095 | 0.304 | 2.543 × 10⁻⁰⁴ | 1.391 × 10⁻⁰² | 7.737 × 10⁻⁰¹ |
| *OR2D3* | -0.198 | -0.300 | -0.091 | 3.459 × 10⁻⁰⁴ | 1.436 × 10⁻⁰² | 7.741 × 10⁻⁰¹ |
| *LOC100287227* | -0.165 | -0.269 | -0.057 | 2.977 × 10⁻⁰³ | 4.782 × 10⁻⁰² | 7.741 × 10⁻⁰¹ |
| *SRPX2* | 0.164 | 0.056 | 0.268 | 3.015 × 10⁻⁰³ | 4.816 × 10⁻⁰² | 7.745 × 10⁻⁰¹ |
| *LEMD2* | -0.176 | -0.280 | -0.068 | 1.468 × 10⁻⁰³ | 3.117 × 10⁻⁰² | 7.750 × 10⁻⁰¹ |
| *PAGE2* | -0.183 | -0.286 | -0.075 | 9.639 × 10⁻⁰⁴ | 2.450 × 10⁻⁰² | 7.757 × 10⁻⁰¹ |
| *ZNF735* | -0.195 | -0.298 | -0.088 | 4.073 × 10⁻⁰⁴ | 1.561 × 10⁻⁰² | 7.801 × 10⁻⁰¹ |
| *ELAVL3* | -0.166 | -0.270 | -0.058 | 2.756 × 10⁻⁰³ | 4.579 × 10⁻⁰² | 7.809 × 10⁻⁰¹ |
| *CABP7* | -0.214 | -0.316 | -0.108 | 1.017 × 10⁻⁰⁴ | 8.755 × 10⁻⁰³ | 7.825 × 10⁻⁰¹ |
| *C10orf140* | -0.204 | -0.306 | -0.097 | 2.209 × 10⁻⁰⁴ | 1.296 × 10⁻⁰² | 7.826 × 10⁻⁰¹ |
| *TICAM1* | 0.241 | 0.135 | 0.341 | 1.169 × 10⁻⁰⁵ | 2.478 × 10⁻⁰³ | 7.847 × 10⁻⁰¹ |
| *ALG9* | -0.215 | -0.316 | -0.109 | 9.639 × 10⁻⁰⁵ | 8.517 × 10⁻⁰³ | 7.862 × 10⁻⁰¹ |
| *C13orf31* | 0.171 | 0.063 | 0.274 | 2.058 × 10⁻⁰³ | 3.837 × 10⁻⁰² | 7.864 × 10⁻⁰¹ |
| *ATXN7L3* | -0.191 | -0.293 | -0.083 | 5.656 × 10⁻⁰⁴ | 1.840 × 10⁻⁰² | 7.866 × 10⁻⁰¹ |
| *HMGN5* | -0.166 | -0.270 | -0.058 | 2.778 × 10⁻⁰³ | 4.603 × 10⁻⁰² | 7.873 × 10⁻⁰¹ |
| *HBQ1* | -0.167 | -0.271 | -0.059 | 2.594 × 10⁻⁰³ | 4.416 × 10⁻⁰² | 7.876 × 10⁻⁰¹ |
| *TNFAIP8L1* | 0.173 | 0.065 | 0.277 | 1.794 × 10⁻⁰³ | 3.511 × 10⁻⁰² | 7.882 × 10⁻⁰¹ |
| *CHST10* | -0.186 | -0.289 | -0.079 | 7.470 × 10⁻⁰⁴ | 2.163 × 10⁻⁰² | 7.889 × 10⁻⁰¹ |
| *TAC3* | -0.165 | -0.269 | -0.057 | 2.864 × 10⁻⁰³ | 4.683 × 10⁻⁰² | 7.890 × 10⁻⁰¹ |
| *KRTAP1.3* | -0.263 | -0.362 | -0.159 | 1.525 × 10⁻⁰⁶ | 7.318 × 10⁻⁰⁴ | 7.898 × 10⁻⁰¹ |
| *MTUS2* | -0.169 | -0.273 | -0.061 | 2.297 × 10⁻⁰³ | 4.120 × 10⁻⁰² | 7.899 × 10⁻⁰¹ |
| *NCRNA00152* | 0.166 | 0.058 | 0.270 | 2.796 × 10⁻⁰³ | 4.607 × 10⁻⁰² | 7.906 × 10⁻⁰¹ |
| *PAX3* | -0.185 | -0.288 | -0.078 | 8.161 × 10⁻⁰⁴ | 2.247 × 10⁻⁰² | 7.910 × 10⁻⁰¹ |
| *USH1C* | -0.168 | -0.272 | -0.060 | 2.434 × 10⁻⁰³ | 4.268 × 10⁻⁰² | 7.931 × 10⁻⁰¹ |
| *PRKCG* | -0.205 | -0.307 | -0.098 | 2.061 × 10⁻⁰⁴ | 1.234 × 10⁻⁰² | 7.936 × 10⁻⁰¹ |
| *TMEM74* | -0.187 | -0.290 | -0.079 | 7.256 × 10⁻⁰⁴ | 2.122 × 10⁻⁰² | 7.939 × 10⁻⁰¹ |
| *DCTD* | 0.183 | 0.076 | 0.287 | 9.100 × 10⁻⁰⁴ | 2.361 × 10⁻⁰² | 7.944 × 10⁻⁰¹ |
| *ZBTB7A* | 0.195 | 0.088 | 0.298 | 4.059 × 10⁻⁰⁴ | 1.561 × 10⁻⁰² | 7.946 × 10⁻⁰¹ |
| *ALX3* | -0.190 | -0.293 | -0.083 | 5.934 × 10⁻⁰⁴ | 1.897 × 10⁻⁰² | 7.961 × 10⁻⁰¹ |
| *XAGE5* | -0.186 | -0.289 | -0.079 | 7.482 × 10⁻⁰⁴ | 2.163 × 10⁻⁰² | 7.970 × 10⁻⁰¹ |
| *OR51B6* | -0.175 | -0.279 | -0.068 | 1.546 × 10⁻⁰³ | 3.196 × 10⁻⁰² | 7.975 × 10⁻⁰¹ |
| *C19orf54* | -0.166 | -0.270 | -0.058 | 2.705 × 10⁻⁰³ | 4.539 × 10⁻⁰² | 7.976 × 10⁻⁰¹ |
| *UCMA* | -0.191 | -0.294 | -0.084 | 5.563 × 10⁻⁰⁴ | 1.821 × 10⁻⁰² | 7.978 × 10⁻⁰¹ |
| *NEURL4* | -0.183 | -0.286 | -0.076 | 9.224 × 10⁻⁰⁴ | 2.384 × 10⁻⁰² | 7.996 × 10⁻⁰¹ |
| *AKAP1* | -0.170 | -0.274 | -0.062 | 2.167 × 10⁻⁰³ | 3.947 × 10⁻⁰² | 7.998 × 10⁻⁰¹ |
| *ASB18* | -0.206 | -0.308 | -0.099 | 1.866 × 10⁻⁰⁴ | 1.187 × 10⁻⁰² | 8.015 × 10⁻⁰¹ |
| *NLE1* | -0.173 | -0.277 | -0.065 | 1.764 × 10⁻⁰³ | 3.467 × 10⁻⁰² | 8.050 × 10⁻⁰¹ |
| *FAM114A1* | 0.207 | 0.100 | 0.309 | 1.729 × 10⁻⁰⁴ | 1.149 × 10⁻⁰² | 8.050 × 10⁻⁰¹ |
| *PRNP* | 0.177 | 0.069 | 0.280 | 1.420 × 10⁻⁰³ | 3.071 × 10⁻⁰² | 8.053 × 10⁻⁰¹ |
| *CDKAL1* | -0.178 | -0.282 | -0.071 | 1.258 × 10⁻⁰³ | 2.873 × 10⁻⁰² | 8.101 × 10⁻⁰¹ |
| *OR10A5* | -0.197 | -0.300 | -0.090 | 3.507 × 10⁻⁰⁴ | 1.447 × 10⁻⁰² | 8.104 × 10⁻⁰¹ |
| *GNAT1* | -0.313 | -0.408 | -0.212 | 8.298 × 10⁻⁰⁹ | 1.866 × 10⁻⁰⁵ | 8.123 × 10⁻⁰¹ |
| *DNAJB7* | -0.191 | -0.294 | -0.084 | 5.427 × 10⁻⁰⁴ | 1.795 × 10⁻⁰² | 8.130 × 10⁻⁰¹ |
| *ZSCAN10* | -0.185 | -0.289 | -0.078 | 7.989 × 10⁻⁰⁴ | 2.224 × 10⁻⁰² | 8.135 × 10⁻⁰¹ |
| *FAM47C* | -0.206 | -0.308 | -0.099 | 1.918 × 10⁻⁰⁴ | 1.194 × 10⁻⁰² | 8.138 × 10⁻⁰¹ |
| *IFNA5* | -0.200 | -0.302 | -0.093 | 3.013 × 10⁻⁰⁴ | 1.433 × 10⁻⁰² | 8.140 × 10⁻⁰¹ |
| *SAPS3* | -0.193 | -0.296 | -0.086 | 4.675 × 10⁻⁰⁴ | 1.690 × 10⁻⁰² | 8.145 × 10⁻⁰¹ |
| *GJD4* | -0.164 | -0.268 | -0.056 | 3.056 × 10⁻⁰³ | 4.846 × 10⁻⁰² | 8.148 × 10⁻⁰¹ |
| *AMIGO2* | 0.200 | 0.093 | 0.302 | 2.996 × 10⁻⁰⁴ | 1.433 × 10⁻⁰² | 8.157 × 10⁻⁰¹ |
| *EWSR1* | -0.168 | -0.272 | -0.060 | 2.434 × 10⁻⁰³ | 4.268 × 10⁻⁰² | 8.170 × 10⁻⁰¹ |
| *LOC402644* | -0.165 | -0.269 | -0.057 | 2.865 × 10⁻⁰³ | 4.683 × 10⁻⁰² | 8.175 × 10⁻⁰¹ |
| *RAB9BP1* | -0.179 | -0.282 | -0.071 | 1.213 × 10⁻⁰³ | 2.804 × 10⁻⁰² | 8.200 × 10⁻⁰¹ |
| *LOC100286844* | -0.175 | -0.279 | -0.068 | 1.544 × 10⁻⁰³ | 3.196 × 10⁻⁰² | 8.206 × 10⁻⁰¹ |
| *CAMK2B* | -0.179 | -0.282 | -0.071 | 1.218 × 10⁻⁰³ | 2.804 × 10⁻⁰² | 8.208 × 10⁻⁰¹ |
| *AAMP* | -0.166 | -0.270 | -0.058 | 2.723 × 10⁻⁰³ | 4.553 × 10⁻⁰² | 8.212 × 10⁻⁰¹ |
| *DNAJC14* | -0.213 | -0.315 | -0.107 | 1.094 × 10⁻⁰⁴ | 8.996 × 10⁻⁰³ | 8.222 × 10⁻⁰¹ |
| *PCGEM1* | -0.182 | -0.286 | -0.075 | 9.680 × 10⁻⁰⁴ | 2.458 × 10⁻⁰² | 8.227 × 10⁻⁰¹ |
| *POU3F3* | -0.218 | -0.319 | -0.111 | 7.843 × 10⁻⁰⁵ | 7.453 × 10⁻⁰³ | 8.237 × 10⁻⁰¹ |
| *MYADML* | -0.226 | -0.327 | -0.120 | 4.157 × 10⁻⁰⁵ | 5.230 × 10⁻⁰³ | 8.237 × 10⁻⁰¹ |
| *OR4D5* | -0.197 | -0.299 | -0.090 | 3.629 × 10⁻⁰⁴ | 1.476 × 10⁻⁰² | 8.251 × 10⁻⁰¹ |
| *DNAJB8* | -0.215 | -0.316 | -0.108 | 9.843 × 10⁻⁰⁵ | 8.652 × 10⁻⁰³ | 8.256 × 10⁻⁰¹ |
| *SDCBP2* | 0.182 | 0.074 | 0.285 | 1.016 × 10⁻⁰³ | 2.531 × 10⁻⁰² | 8.260 × 10⁻⁰¹ |
| *MAP2K6* | -0.193 | -0.296 | -0.086 | 4.677 × 10⁻⁰⁴ | 1.690 × 10⁻⁰² | 8.263 × 10⁻⁰¹ |
| *PROP1* | -0.180 | -0.283 | -0.072 | 1.154 × 10⁻⁰³ | 2.736 × 10⁻⁰² | 8.280 × 10⁻⁰¹ |
| *ABT1* | -0.171 | -0.275 | -0.063 | 2.028 × 10⁻⁰³ | 3.803 × 10⁻⁰² | 8.294 × 10⁻⁰¹ |
| *PLSCR5* | -0.168 | -0.272 | -0.060 | 2.422 × 10⁻⁰³ | 4.260 × 10⁻⁰² | 8.299 × 10⁻⁰¹ |
| *TUBAL3* | 0.182 | 0.074 | 0.285 | 1.006 × 10⁻⁰³ | 2.517 × 10⁻⁰² | 8.302 × 10⁻⁰¹ |
| *FAM125A* | 0.167 | 0.059 | 0.271 | 2.528 × 10⁻⁰³ | 4.343 × 10⁻⁰² | 8.323 × 10⁻⁰¹ |
| *GPR68* | 0.193 | 0.085 | 0.295 | 4.921 × 10⁻⁰⁴ | 1.726 × 10⁻⁰² | 8.327 × 10⁻⁰¹ |
| *OR51Q1* | -0.176 | -0.279 | -0.068 | 1.511 × 10⁻⁰³ | 3.162 × 10⁻⁰² | 8.327 × 10⁻⁰¹ |
| *PRAMEF11* | -0.176 | -0.279 | -0.068 | 1.483 × 10⁻⁰³ | 3.133 × 10⁻⁰² | 8.329 × 10⁻⁰¹ |
| *TACR3* | -0.275 | -0.373 | -0.172 | 4.788 × 10⁻⁰⁷ | 3.726 × 10⁻⁰⁴ | 8.373 × 10⁻⁰¹ |
| *BEND5* | -0.170 | -0.274 | -0.062 | 2.121 × 10⁻⁰³ | 3.898 × 10⁻⁰² | 8.383 × 10⁻⁰¹ |
| *OR52E2* | -0.203 | -0.305 | -0.096 | 2.429 × 10⁻⁰⁴ | 1.349 × 10⁻⁰² | 8.389 × 10⁻⁰¹ |
| *RNF214* | -0.186 | -0.289 | -0.079 | 7.634 × 10⁻⁰⁴ | 2.194 × 10⁻⁰² | 8.414 × 10⁻⁰¹ |
| *OR13C3* | -0.215 | -0.317 | -0.109 | 9.400 × 10⁻⁰⁵ | 8.417 × 10⁻⁰³ | 8.419 × 10⁻⁰¹ |
| *ACSM4* | -0.172 | -0.276 | -0.064 | 1.888 × 10⁻⁰³ | 3.608 × 10⁻⁰² | 8.421 × 10⁻⁰¹ |
| *PIM1* | 0.166 | 0.059 | 0.270 | 2.658 × 10⁻⁰³ | 4.496 × 10⁻⁰² | 8.428 × 10⁻⁰¹ |
| *CTSB* | 0.200 | 0.093 | 0.303 | 2.886 × 10⁻⁰⁴ | 1.433 × 10⁻⁰² | 8.441 × 10⁻⁰¹ |
| *C10orf96* | -0.341 | -0.434 | -0.241 | 2.885 × 10⁻¹⁰ | 1.658 × 10⁻⁰⁶ | 8.441 × 10⁻⁰¹ |
| *HTR5A* | -0.237 | -0.338 | -0.132 | 1.579 × 10⁻⁰⁵ | 3.042 × 10⁻⁰³ | 8.442 × 10⁻⁰¹ |
| *CCDC144NL* | -0.223 | -0.324 | -0.117 | 5.022 × 10⁻⁰⁵ | 5.903 × 10⁻⁰³ | 8.446 × 10⁻⁰¹ |
| *VLDLR* | 0.173 | 0.066 | 0.277 | 1.737 × 10⁻⁰³ | 3.448 × 10⁻⁰² | 8.450 × 10⁻⁰¹ |
| *PATZ1* | -0.245 | -0.345 | -0.140 | 8.060 × 10⁻⁰⁶ | 2.204 × 10⁻⁰³ | 8.457 × 10⁻⁰¹ |
| *GNL1* | -0.241 | -0.341 | -0.135 | 1.178 × 10⁻⁰⁵ | 2.478 × 10⁻⁰³ | 8.458 × 10⁻⁰¹ |
| *TSPAN10* | 0.196 | 0.089 | 0.298 | 3.945 × 10⁻⁰⁴ | 1.532 × 10⁻⁰² | 8.473 × 10⁻⁰¹ |
| *LOC285740* | -0.164 | -0.268 | -0.056 | 3.071 × 10⁻⁰³ | 4.859 × 10⁻⁰² | 8.483 × 10⁻⁰¹ |
| *HTATSF1* | -0.171 | -0.275 | -0.063 | 2.030 × 10⁻⁰³ | 3.803 × 10⁻⁰² | 8.493 × 10⁻⁰¹ |
| *ASTL* | -0.167 | -0.271 | -0.059 | 2.516 × 10⁻⁰³ | 4.338 × 10⁻⁰² | 8.506 × 10⁻⁰¹ |
| *NHEDC2* | 0.170 | 0.062 | 0.274 | 2.124 × 10⁻⁰³ | 3.898 × 10⁻⁰² | 8.506 × 10⁻⁰¹ |
| *LBXCOR1* | -0.205 | -0.307 | -0.098 | 2.100 × 10⁻⁰⁴ | 1.246 × 10⁻⁰² | 8.511 × 10⁻⁰¹ |
| *FRG2C* | -0.282 | -0.379 | -0.178 | 2.463 × 10⁻⁰⁷ | 2.373 × 10⁻⁰⁴ | 8.518 × 10⁻⁰¹ |
| *BRDT* | -0.181 | -0.285 | -0.074 | 1.050 × 10⁻⁰³ | 2.570 × 10⁻⁰² | 8.526 × 10⁻⁰¹ |
| *TLR6* | 0.164 | 0.056 | 0.269 | 2.999 × 10⁻⁰³ | 4.806 × 10⁻⁰² | 8.532 × 10⁻⁰¹ |
| *OSTF1* | 0.180 | 0.072 | 0.283 | 1.155 × 10⁻⁰³ | 2.736 × 10⁻⁰² | 8.544 × 10⁻⁰¹ |
| *C6orf163* | -0.175 | -0.279 | -0.067 | 1.576 × 10⁻⁰³ | 3.233 × 10⁻⁰² | 8.548 × 10⁻⁰¹ |
| *TM4SF1* | 0.188 | 0.080 | 0.291 | 6.908 × 10⁻⁰⁴ | 2.069 × 10⁻⁰² | 8.555 × 10⁻⁰¹ |
| *SEPT14* | -0.169 | -0.273 | -0.062 | 2.224 × 10⁻⁰³ | 4.030 × 10⁻⁰² | 8.579 × 10⁻⁰¹ |
| *TPO* | -0.188 | -0.291 | -0.080 | 6.932 × 10⁻⁰⁴ | 2.069 × 10⁻⁰² | 8.580 × 10⁻⁰¹ |
| *DPP9* | 0.191 | 0.083 | 0.293 | 5.621 × 10⁻⁰⁴ | 1.831 × 10⁻⁰² | 8.584 × 10⁻⁰¹ |
| *ZBTB12* | -0.255 | -0.354 | -0.150 | 3.302 × 10⁻⁰⁶ | 1.193 × 10⁻⁰³ | 8.585 × 10⁻⁰¹ |
| *GJA10* | -0.168 | -0.272 | -0.060 | 2.399 × 10⁻⁰³ | 4.240 × 10⁻⁰² | 8.588 × 10⁻⁰¹ |
| *RNF40* | -0.170 | -0.274 | -0.062 | 2.140 × 10⁻⁰³ | 3.914 × 10⁻⁰² | 8.603 × 10⁻⁰¹ |
| *OR9Q2* | -0.179 | -0.283 | -0.072 | 1.194 × 10⁻⁰³ | 2.784 × 10⁻⁰² | 8.612 × 10⁻⁰¹ |
| *ABHD5* | 0.165 | 0.057 | 0.269 | 2.924 × 10⁻⁰³ | 4.734 × 10⁻⁰² | 8.626 × 10⁻⁰¹ |
| *CARD11* | 0.179 | 0.071 | 0.282 | 1.217 × 10⁻⁰³ | 2.804 × 10⁻⁰² | 8.628 × 10⁻⁰¹ |
| *ALLC* | -0.173 | -0.276 | -0.065 | 1.819 × 10⁻⁰³ | 3.536 × 10⁻⁰² | 8.680 × 10⁻⁰¹ |
| *VN1R5* | -0.286 | -0.383 | -0.183 | 1.564 × 10⁻⁰⁷ | 1.758 × 10⁻⁰⁴ | 8.681 × 10⁻⁰¹ |
| *CCDC140* | -0.223 | -0.324 | -0.117 | 5.158 × 10⁻⁰⁵ | 5.930 × 10⁻⁰³ | 8.698 × 10⁻⁰¹ |
| *C7orf4* | -0.168 | -0.272 | -0.060 | 2.484 × 10⁻⁰³ | 4.289 × 10⁻⁰² | 8.700 × 10⁻⁰¹ |
| *CXorf48* | -0.191 | -0.294 | -0.084 | 5.320 × 10⁻⁰⁴ | 1.782 × 10⁻⁰² | 8.717 × 10⁻⁰¹ |
| *FEV* | -0.200 | -0.302 | -0.093 | 2.926 × 10⁻⁰⁴ | 1.433 × 10⁻⁰² | 8.726 × 10⁻⁰¹ |
| *GTF3C1* | -0.175 | -0.278 | -0.067 | 1.596 × 10⁻⁰³ | 3.262 × 10⁻⁰² | 8.733 × 10⁻⁰¹ |
| *HIRIP3* | -0.243 | -0.343 | -0.137 | 9.964 × 10⁻⁰⁶ | 2.454 × 10⁻⁰³ | 8.742 × 10⁻⁰¹ |
| *BEND3* | -0.196 | -0.298 | -0.089 | 3.926 × 10⁻⁰⁴ | 1.528 × 10⁻⁰² | 8.747 × 10⁻⁰¹ |
| *OR13C5* | -0.211 | -0.313 | -0.105 | 1.281 × 10⁻⁰⁴ | 9.894 × 10⁻⁰³ | 8.747 × 10⁻⁰¹ |
| *KRTAP10.11* | -0.169 | -0.273 | -0.061 | 2.294 × 10⁻⁰³ | 4.120 × 10⁻⁰² | 8.774 × 10⁻⁰¹ |
| *CACNG7* | -0.204 | -0.306 | -0.097 | 2.236 × 10⁻⁰⁴ | 1.304 × 10⁻⁰² | 8.792 × 10⁻⁰¹ |
| *TUBA3C* | -0.210 | -0.312 | -0.103 | 1.415 × 10⁻⁰⁴ | 1.035 × 10⁻⁰² | 8.808 × 10⁻⁰¹ |
| *BPTF* | -0.163 | -0.268 | -0.055 | 3.189 × 10⁻⁰³ | 4.984 × 10⁻⁰² | 8.815 × 10⁻⁰¹ |
| *MTNR1B* | -0.168 | -0.272 | -0.060 | 2.484 × 10⁻⁰³ | 4.289 × 10⁻⁰² | 8.816 × 10⁻⁰¹ |
| *EPHA6* | -0.194 | -0.296 | -0.087 | 4.530 × 10⁻⁰⁴ | 1.664 × 10⁻⁰² | 8.852 × 10⁻⁰¹ |
| *GPR119* | -0.177 | -0.281 | -0.069 | 1.375 × 10⁻⁰³ | 3.038 × 10⁻⁰² | 8.872 × 10⁻⁰¹ |
| *DAPP1* | 0.212 | 0.105 | 0.313 | 1.233 × 10⁻⁰⁴ | 9.632 × 10⁻⁰³ | 8.898 × 10⁻⁰¹ |
| *QRSL1* | -0.166 | -0.270 | -0.058 | 2.805 × 10⁻⁰³ | 4.614 × 10⁻⁰² | 8.900 × 10⁻⁰¹ |
| *DNAJB4* | 0.169 | 0.061 | 0.273 | 2.253 × 10⁻⁰³ | 4.070 × 10⁻⁰² | 8.905 × 10⁻⁰¹ |
| *CHRNA4* | -0.169 | -0.273 | -0.061 | 2.267 × 10⁻⁰³ | 4.085 × 10⁻⁰² | 8.914 × 10⁻⁰¹ |
| *CDH7* | -0.181 | -0.285 | -0.074 | 1.033 × 10⁻⁰³ | 2.555 × 10⁻⁰² | 8.930 × 10⁻⁰¹ |
| *OR6T1* | -0.175 | -0.279 | -0.068 | 1.539 × 10⁻⁰³ | 3.193 × 10⁻⁰² | 8.936 × 10⁻⁰¹ |
| *MYH4* | -0.175 | -0.279 | -0.068 | 1.523 × 10⁻⁰³ | 3.177 × 10⁻⁰² | 8.938 × 10⁻⁰¹ |
| *SAMD9* | 0.191 | 0.083 | 0.294 | 5.605 × 10⁻⁰⁴ | 1.829 × 10⁻⁰² | 8.945 × 10⁻⁰¹ |
| *LHFPL1* | -0.193 | -0.296 | -0.086 | 4.845 × 10⁻⁰⁴ | 1.711 × 10⁻⁰² | 8.948 × 10⁻⁰¹ |
| *GIP* | -0.189 | -0.292 | -0.081 | 6.447 × 10⁻⁰⁴ | 1.979 × 10⁻⁰² | 8.953 × 10⁻⁰¹ |
| *MSTN* | -0.168 | -0.272 | -0.060 | 2.421 × 10⁻⁰³ | 4.260 × 10⁻⁰² | 8.957 × 10⁻⁰¹ |
| *DDX6* | -0.184 | -0.287 | -0.077 | 8.678 × 10⁻⁰⁴ | 2.301 × 10⁻⁰² | 8.979 × 10⁻⁰¹ |
| *ST8SIA5* | -0.223 | -0.324 | -0.117 | 5.197 × 10⁻⁰⁵ | 5.938 × 10⁻⁰³ | 8.991 × 10⁻⁰¹ |
| *SLC38A3* | -0.175 | -0.278 | -0.067 | 1.612 × 10⁻⁰³ | 3.284 × 10⁻⁰² | 8.993 × 10⁻⁰¹ |
| *MAGEA12* | -0.176 | -0.280 | -0.069 | 1.454 × 10⁻⁰³ | 3.104 × 10⁻⁰² | 8.999 × 10⁻⁰¹ |
| *OTUD3* | -0.201 | -0.303 | -0.094 | 2.699 × 10⁻⁰⁴ | 1.433 × 10⁻⁰² | 9.023 × 10⁻⁰¹ |
| *C9orf95* | 0.179 | 0.071 | 0.282 | 1.240 × 10⁻⁰³ | 2.849 × 10⁻⁰² | 9.033 × 10⁻⁰¹ |
| *SLC25A43* | 0.190 | 0.082 | 0.293 | 6.023 × 10⁻⁰⁴ | 1.911 × 10⁻⁰² | 9.040 × 10⁻⁰¹ |
| *ARL8B* | 0.185 | 0.078 | 0.288 | 8.060 × 10⁻⁰⁴ | 2.231 × 10⁻⁰² | 9.043 × 10⁻⁰¹ |
| *SART1* | -0.260 | -0.359 | -0.156 | 2.065 × 10⁻⁰⁶ | 8.792 × 10⁻⁰⁴ | 9.044 × 10⁻⁰¹ |
| *HNRNPUL2* | -0.194 | -0.297 | -0.087 | 4.405 × 10⁻⁰⁴ | 1.641 × 10⁻⁰² | 9.055 × 10⁻⁰¹ |
| *ACTR3B* | -0.164 | -0.268 | -0.056 | 3.103 × 10⁻⁰³ | 4.897 × 10⁻⁰² | 9.055 × 10⁻⁰¹ |
| *TTC27* | -0.174 | -0.278 | -0.067 | 1.630 × 10⁻⁰³ | 3.316 × 10⁻⁰² | 9.056 × 10⁻⁰¹ |
| *AMAC1L3* | -0.198 | -0.300 | -0.091 | 3.439 × 10⁻⁰⁴ | 1.433 × 10⁻⁰² | 9.056 × 10⁻⁰¹ |
| *KRT28* | -0.253 | -0.352 | -0.148 | 3.982 × 10⁻⁰⁶ | 1.343 × 10⁻⁰³ | 9.062 × 10⁻⁰¹ |
| *BCOR* | -0.207 | -0.309 | -0.100 | 1.785 × 10⁻⁰⁴ | 1.167 × 10⁻⁰² | 9.076 × 10⁻⁰¹ |
| *EP400* | -0.178 | -0.282 | -0.071 | 1.260 × 10⁻⁰³ | 2.874 × 10⁻⁰² | 9.076 × 10⁻⁰¹ |
| *ZNF142* | -0.174 | -0.277 | -0.066 | 1.694 × 10⁻⁰³ | 3.386 × 10⁻⁰² | 9.098 × 10⁻⁰¹ |
| *PHF12* | -0.214 | -0.316 | -0.108 | 1.025 × 10⁻⁰⁴ | 8.755 × 10⁻⁰³ | 9.107 × 10⁻⁰¹ |
| *UGCG* | 0.166 | 0.058 | 0.270 | 2.790 × 10⁻⁰³ | 4.607 × 10⁻⁰² | 9.109 × 10⁻⁰¹ |
| *WNK3* | -0.175 | -0.278 | -0.067 | 1.608 × 10⁻⁰³ | 3.283 × 10⁻⁰² | 9.121 × 10⁻⁰¹ |
| *OR51M1* | -0.184 | -0.288 | -0.077 | 8.507 × 10⁻⁰⁴ | 2.282 × 10⁻⁰² | 9.128 × 10⁻⁰¹ |
| *SARS2* | -0.178 | -0.282 | -0.070 | 1.288 × 10⁻⁰³ | 2.914 × 10⁻⁰² | 9.139 × 10⁻⁰¹ |
| *ELP2P* | -0.199 | -0.301 | -0.092 | 3.156 × 10⁻⁰⁴ | 1.433 × 10⁻⁰² | 9.168 × 10⁻⁰¹ |
| *SUPT6H* | -0.185 | -0.288 | -0.077 | 8.396 × 10⁻⁰⁴ | 2.274 × 10⁻⁰² | 9.179 × 10⁻⁰¹ |
| *LOC145837* | -0.182 | -0.285 | -0.075 | 9.902 × 10⁻⁰⁴ | 2.498 × 10⁻⁰² | 9.191 × 10⁻⁰¹ |
| *PRAMEF4* | -0.190 | -0.293 | -0.083 | 5.771 × 10⁻⁰⁴ | 1.863 × 10⁻⁰² | 9.200 × 10⁻⁰¹ |
| *NCOA5* | -0.194 | -0.297 | -0.087 | 4.501 × 10⁻⁰⁴ | 1.664 × 10⁻⁰² | 9.205 × 10⁻⁰¹ |
| *NFRKB* | -0.220 | -0.321 | -0.114 | 6.443 × 10⁻⁰⁵ | 6.862 × 10⁻⁰³ | 9.208 × 10⁻⁰¹ |
| *OR52E8* | -0.191 | -0.294 | -0.084 | 5.441 × 10⁻⁰⁴ | 1.796 × 10⁻⁰² | 9.227 × 10⁻⁰¹ |
| *TUBA4A* | 0.184 | 0.076 | 0.287 | 8.816 × 10⁻⁰⁴ | 2.312 × 10⁻⁰² | 9.258 × 10⁻⁰¹ |
| *TULP1* | -0.173 | -0.277 | -0.065 | 1.752 × 10⁻⁰³ | 3.459 × 10⁻⁰² | 9.270 × 10⁻⁰¹ |
| *TRIM26* | -0.168 | -0.272 | -0.060 | 2.429 × 10⁻⁰³ | 4.267 × 10⁻⁰² | 9.273 × 10⁻⁰¹ |
| *RAP1GDS1* | 0.192 | 0.085 | 0.295 | 5.097 × 10⁻⁰⁴ | 1.757 × 10⁻⁰² | 9.278 × 10⁻⁰¹ |
| *ASAP1IT1* | -0.241 | -0.341 | -0.135 | 1.173 × 10⁻⁰⁵ | 2.478 × 10⁻⁰³ | 9.319 × 10⁻⁰¹ |
| *HNRNPH3* | -0.180 | -0.283 | -0.072 | 1.170 × 10⁻⁰³ | 2.751 × 10⁻⁰² | 9.320 × 10⁻⁰¹ |
| *ZNF804B* | -0.270 | -0.368 | -0.165 | 8.471 × 10⁻⁰⁷ | 5.357 × 10⁻⁰⁴ | 9.321 × 10⁻⁰¹ |
| *DDX51* | -0.219 | -0.321 | -0.113 | 6.814 × 10⁻⁰⁵ | 7.131 × 10⁻⁰³ | 9.322 × 10⁻⁰¹ |
| *ATP6V1C2* | -0.170 | -0.273 | -0.062 | 2.195 × 10⁻⁰³ | 3.991 × 10⁻⁰² | 9.325 × 10⁻⁰¹ |
| *RBPJL* | -0.255 | -0.354 | -0.150 | 3.446 × 10⁻⁰⁶ | 1.223 × 10⁻⁰³ | 9.348 × 10⁻⁰¹ |
| *MAPKAPK5* | -0.176 | -0.280 | -0.069 | 1.446 × 10⁻⁰³ | 3.095 × 10⁻⁰² | 9.359 × 10⁻⁰¹ |
| *KCNH5* | -0.254 | -0.354 | -0.150 | 3.514 × 10⁻⁰⁶ | 1.226 × 10⁻⁰³ | 9.365 × 10⁻⁰¹ |
| *PPP1R10* | -0.231 | -0.331 | -0.125 | 2.785 × 10⁻⁰⁵ | 4.174 × 10⁻⁰³ | 9.366 × 10⁻⁰¹ |
| *TUT1* | -0.163 | -0.268 | -0.055 | 3.174 × 10⁻⁰³ | 4.974 × 10⁻⁰² | 9.369 × 10⁻⁰¹ |
| *PLA2G4C* | 0.166 | 0.058 | 0.270 | 2.678 × 10⁻⁰³ | 4.515 × 10⁻⁰² | 9.372 × 10⁻⁰¹ |
| *RAPSN* | -0.203 | -0.305 | -0.096 | 2.333 × 10⁻⁰⁴ | 1.324 × 10⁻⁰² | 9.383 × 10⁻⁰¹ |
| *GPS2* | -0.189 | -0.292 | -0.082 | 6.291 × 10⁻⁰⁴ | 1.946 × 10⁻⁰² | 9.400 × 10⁻⁰¹ |
| *STK17A* | 0.196 | 0.089 | 0.299 | 3.867 × 10⁻⁰⁴ | 1.521 × 10⁻⁰² | 9.405 × 10⁻⁰¹ |
| *TRIM42* | -0.188 | -0.291 | -0.081 | 6.785 × 10⁻⁰⁴ | 2.043 × 10⁻⁰² | 9.416 × 10⁻⁰¹ |
| *MAGEA8* | -0.188 | -0.291 | -0.081 | 6.547 × 10⁻⁰⁴ | 1.992 × 10⁻⁰² | 9.433 × 10⁻⁰¹ |
| *OR13C2* | -0.195 | -0.298 | -0.088 | 4.098 × 10⁻⁰⁴ | 1.564 × 10⁻⁰² | 9.443 × 10⁻⁰¹ |
| *ZNF48* | -0.164 | -0.268 | -0.056 | 3.043 × 10⁻⁰³ | 4.836 × 10⁻⁰² | 9.458 × 10⁻⁰¹ |
| *CSMD1* | -0.197 | -0.300 | -0.090 | 3.600 × 10⁻⁰⁴ | 1.472 × 10⁻⁰² | 9.460 × 10⁻⁰¹ |
| *LOC144486* | -0.180 | -0.284 | -0.073 | 1.101 × 10⁻⁰³ | 2.653 × 10⁻⁰² | 9.460 × 10⁻⁰¹ |
| *FBXO8* | 0.174 | 0.066 | 0.278 | 1.657 × 10⁻⁰³ | 3.347 × 10⁻⁰² | 9.472 × 10⁻⁰¹ |
| *BSND* | -0.192 | -0.295 | -0.085 | 5.079 × 10⁻⁰⁴ | 1.754 × 10⁻⁰² | 9.474 × 10⁻⁰¹ |
| *ABCG5* | -0.223 | -0.324 | -0.117 | 5.078 × 10⁻⁰⁵ | 5.903 × 10⁻⁰³ | 9.484 × 10⁻⁰¹ |
| *CLRN1OS* | -0.207 | -0.309 | -0.101 | 1.696 × 10⁻⁰⁴ | 1.140 × 10⁻⁰² | 9.505 × 10⁻⁰¹ |
| *DCAF8L2* | -0.185 | -0.288 | -0.077 | 8.433 × 10⁻⁰⁴ | 2.275 × 10⁻⁰² | 9.526 × 10⁻⁰¹ |
| *EHMT2* | -0.246 | -0.345 | -0.141 | 7.665 × 10⁻⁰⁶ | 2.162 × 10⁻⁰³ | 9.535 × 10⁻⁰¹ |
| *ACVR1B* | -0.164 | -0.268 | -0.056 | 3.053 × 10⁻⁰³ | 4.845 × 10⁻⁰² | 9.537 × 10⁻⁰¹ |
| *ODZ1* | -0.168 | -0.272 | -0.060 | 2.480 × 10⁻⁰³ | 4.289 × 10⁻⁰² | 9.547 × 10⁻⁰¹ |
| *CCDC121* | -0.192 | -0.294 | -0.084 | 5.268 × 10⁻⁰⁴ | 1.772 × 10⁻⁰² | 9.551 × 10⁻⁰¹ |
| *GPR83* | -0.300 | -0.396 | -0.198 | 3.607 × 10⁻⁰⁸ | 5.614 × 10⁻⁰⁵ | 9.555 × 10⁻⁰¹ |
| *OR2A5* | -0.173 | -0.277 | -0.066 | 1.733 × 10⁻⁰³ | 3.448 × 10⁻⁰² | 9.556 × 10⁻⁰¹ |
| *TPRX1* | -0.221 | -0.322 | -0.115 | 6.076 × 10⁻⁰⁵ | 6.574 × 10⁻⁰³ | 9.561 × 10⁻⁰¹ |
| *LDHC* | -0.163 | -0.267 | -0.055 | 3.203 × 10⁻⁰³ | 4.989 × 10⁻⁰² | 9.565 × 10⁻⁰¹ |
| *FLJ42393* | -0.189 | -0.292 | -0.082 | 6.166 × 10⁻⁰⁴ | 1.927 × 10⁻⁰² | 9.574 × 10⁻⁰¹ |
| *ATP10D* | 0.177 | 0.069 | 0.280 | 1.421 × 10⁻⁰³ | 3.071 × 10⁻⁰² | 9.608 × 10⁻⁰¹ |
| *NEUROG2* | -0.180 | -0.284 | -0.073 | 1.119 × 10⁻⁰³ | 2.677 × 10⁻⁰² | 9.641 × 10⁻⁰¹ |
| *MUTYH* | -0.205 | -0.307 | -0.099 | 1.975 × 10⁻⁰⁴ | 1.200 × 10⁻⁰² | 9.641 × 10⁻⁰¹ |
| *POM121L2* | -0.201 | -0.304 | -0.094 | 2.671 × 10⁻⁰⁴ | 1.430 × 10⁻⁰² | 9.642 × 10⁻⁰¹ |
| *NR2C1* | -0.184 | -0.287 | -0.077 | 8.750 × 10⁻⁰⁴ | 2.305 × 10⁻⁰² | 9.649 × 10⁻⁰¹ |
| *TSPYL3* | -0.175 | -0.278 | -0.067 | 1.611 × 10⁻⁰³ | 3.284 × 10⁻⁰² | 9.666 × 10⁻⁰¹ |
| *DHX16* | -0.214 | -0.316 | -0.107 | 1.044 × 10⁻⁰⁴ | 8.766 × 10⁻⁰³ | 9.681 × 10⁻⁰¹ |
| *VSTM2B* | -0.223 | -0.324 | -0.117 | 5.223 × 10⁻⁰⁵ | 5.938 × 10⁻⁰³ | 9.697 × 10⁻⁰¹ |
| *MAF* | 0.168 | 0.060 | 0.272 | 2.472 × 10⁻⁰³ | 4.289 × 10⁻⁰² | 9.709 × 10⁻⁰¹ |
| *MYCN* | -0.165 | -0.269 | -0.057 | 2.882 × 10⁻⁰³ | 4.693 × 10⁻⁰² | 9.712 × 10⁻⁰¹ |
| *KRT38* | -0.167 | -0.271 | -0.059 | 2.589 × 10⁻⁰³ | 4.416 × 10⁻⁰² | 9.726 × 10⁻⁰¹ |
| *ZNRF4* | -0.243 | -0.343 | -0.138 | 9.526 × 10⁻⁰⁶ | 2.409 × 10⁻⁰³ | 9.732 × 10⁻⁰¹ |
| *ZNF618* | -0.166 | -0.270 | -0.058 | 2.792 × 10⁻⁰³ | 4.607 × 10⁻⁰² | 9.741 × 10⁻⁰¹ |
| *RNF138P1* | -0.211 | -0.313 | -0.104 | 1.313 × 10⁻⁰⁴ | 1.006 × 10⁻⁰² | 9.750 × 10⁻⁰¹ |
| *EMID2* | -0.178 | -0.281 | -0.070 | 1.308 × 10⁻⁰³ | 2.943 × 10⁻⁰² | 9.793 × 10⁻⁰¹ |
| *CLDN25* | -0.189 | -0.292 | -0.082 | 6.149 × 10⁻⁰⁴ | 1.926 × 10⁻⁰² | 9.814 × 10⁻⁰¹ |
| *GOSR1* | -0.166 | -0.270 | -0.058 | 2.786 × 10⁻⁰³ | 4.607 × 10⁻⁰² | 9.827 × 10⁻⁰¹ |
| *GUSBL1* | -0.204 | -0.306 | -0.098 | 2.133 × 10⁻⁰⁴ | 1.262 × 10⁻⁰² | 9.838 × 10⁻⁰¹ |
| *GALP* | -0.171 | -0.275 | -0.063 | 2.032 × 10⁻⁰³ | 3.803 × 10⁻⁰² | 9.862 × 10⁻⁰¹ |
| *GAD2* | -0.199 | -0.302 | -0.092 | 3.105 × 10⁻⁰⁴ | 1.433 × 10⁻⁰² | 9.866 × 10⁻⁰¹ |
| *PRAC* | -0.165 | -0.269 | -0.057 | 2.974 × 10⁻⁰³ | 4.781 × 10⁻⁰² | 9.867 × 10⁻⁰¹ |
| *TBC1D28* | -0.310 | -0.405 | -0.208 | 1.248 × 10⁻⁰⁸ | 2.525 × 10⁻⁰⁵ | 9.870 × 10⁻⁰¹ |
| *OR52L1* | -0.221 | -0.322 | -0.114 | 6.242 × 10⁻⁰⁵ | 6.718 × 10⁻⁰³ | 9.879 × 10⁻⁰¹ |
| *TAC4* | -0.164 | -0.268 | -0.056 | 3.017 × 10⁻⁰³ | 4.816 × 10⁻⁰² | 9.895 × 10⁻⁰¹ |
| *OR56A1* | -0.169 | -0.273 | -0.061 | 2.307 × 10⁻⁰³ | 4.125 × 10⁻⁰² | 9.903 × 10⁻⁰¹ |
| *PWRN1* | -0.163 | -0.268 | -0.055 | 3.197 × 10⁻⁰³ | 4.988 × 10⁻⁰² | 9.905 × 10⁻⁰¹ |
| *TAS2R7* | -0.183 | -0.286 | -0.075 | 9.400 × 10⁻⁰⁴ | 2.405 × 10⁻⁰² | 9.909 × 10⁻⁰¹ |
| *RAD17* | -0.198 | -0.301 | -0.091 | 3.319 × 10⁻⁰⁴ | 1.433 × 10⁻⁰² | 9.912 × 10⁻⁰¹ |
| *CHD4* | -0.184 | -0.287 | -0.077 | 8.741 × 10⁻⁰⁴ | 2.305 × 10⁻⁰² | 9.917 × 10⁻⁰¹ |
| *ZKSCAN2* | -0.236 | -0.336 | -0.130 | 1.810 × 10⁻⁰⁵ | 3.284 × 10⁻⁰³ | 9.949 × 10⁻⁰¹ |
| *ANP32A* | -0.242 | -0.342 | -0.137 | 1.024 × 10⁻⁰⁵ | 2.454 × 10⁻⁰³ | 9.955 × 10⁻⁰¹ |
| *TSG1* | -0.236 | -0.336 | -0.130 | 1.798 × 10⁻⁰⁵ | 3.284 × 10⁻⁰³ | 9.965 × 10⁻⁰¹ |

^*a^ *P* values for correlation analysis results.

^*b^ *P* values for gene expression-survival association results. *P* values were derived from Cox proportional hazards model adjusted for age, sex, smoking status, and clinical stage.

**Table S9.** Results of cg12821679*_MAPRE3_*-smoking cessation interaction analysis in LUAD and LUSC patients.

| Probe | Population | HR | 95% CI | | *P* |
| --- | --- | --- | --- | --- | --- |
| cg12821679*_MAPRE3_* | LUAD | 0.820 | 0.292 | 2.298 | 0.7052 |
|  | LUSC | 0.701 | 0.318 | 1.544 | 0.3781 |

The analysis was conducted in combined dataset.

**Table S10.** Results of *MAPRE3* expression-smoking cessation interaction analysis in NSCLC patients.

| Gene | Analysis | HR | 95% CI | | *P* |
| --- | --- | --- | --- | --- | --- |
| *MAPRE3* | Primary^*a^ | 0.69 | 0.502 | 0.962 | 0.0282 |
|  | Sensitivity^*b^ | 0.70 | 0.503 | 0.979 | 0.0373 |

The analysis was conducted in TCGA population.

^*a^ In primary analysis, the variable smoking cessation was encoded as ‘yes’ for former and ‘no’ for current smokers”.

^*b^ In sensitivity analysis, the variable smoking cessation was encoded as ‘yes’ for both former and never smokers, and ‘no’ for current smokers.

**Table S11.** Results of proportional hazards test for *MAPRE3* expression in NSCLC samples.

| Population | TCGA dataset^*a^ | |
| --- | --- | --- |
|  | *χ*^2^ | *P* |
| Low *MAPRE3* expression | 3.060 | 0.08 |
| High *MAPRE3* expression | 0.540 | 0.46 |

^*a^ Gene expression analyses were conducted only in TCGA dataset.

Patients were stratified into high and low expression groups based on the median *MAPRE3* expression. The variable of interest was smoking cessation, which was encoded as ‘yes’ for former and ‘no’ for current smokers.

**Fig. S1.** **Quality control procedures for epigenome-wide DNA methylation data.** DNA methylation was evaluated using Illumina Infinium HumanMethylation450 BeadChips (Illumina Inc.). Raw image data were processed in Genome Studio Methylation Module V1.8 (Illumina Inc.) to compute methylation signals, with normalization, background subtraction, and quality control (QC) performed. Methylation signals underwent quantile normalization (using the *betaqn* function in R package *minfi)*, correction for type I and II probes (using the *BMIQ* function in R package *lumi*), and batch effect adjustment (using the *ComBat* function in R package *sva*).


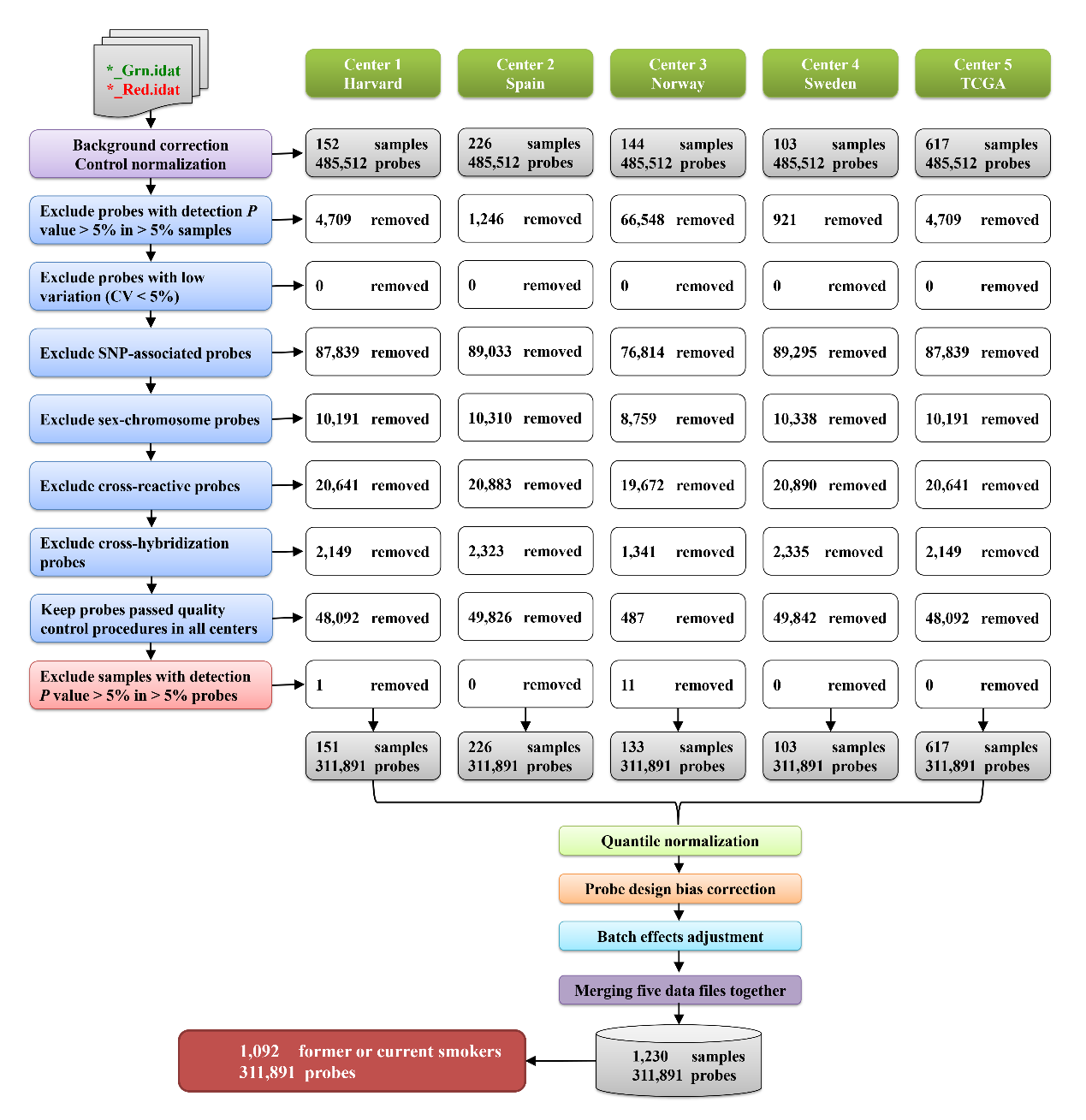


**Fig. S2. Meta-analysis of association between DNA methylation and LUSC prognosis from four cohorts (USA-HSPH, Spain, Sweden and TCGA).** The Norway cohort comprised only LUAD cases, with no LUSC cases. Fixed-effect and random-effects models were both applied, and effect heterogeneity among four cohorts was tested. Diamond represents the pooled effect of cg12821679*_MAPRE3_* among four cohorts. *MAPRE3*, Microtubule-associated protein RP/EB family member 3; LUSC, lung squamous cell carcinoma; LUAD, lung adenocarcinoma; TCGA, The Cancer Genome Atlas; *HR*, hazard ratio; CI, confidence interval.


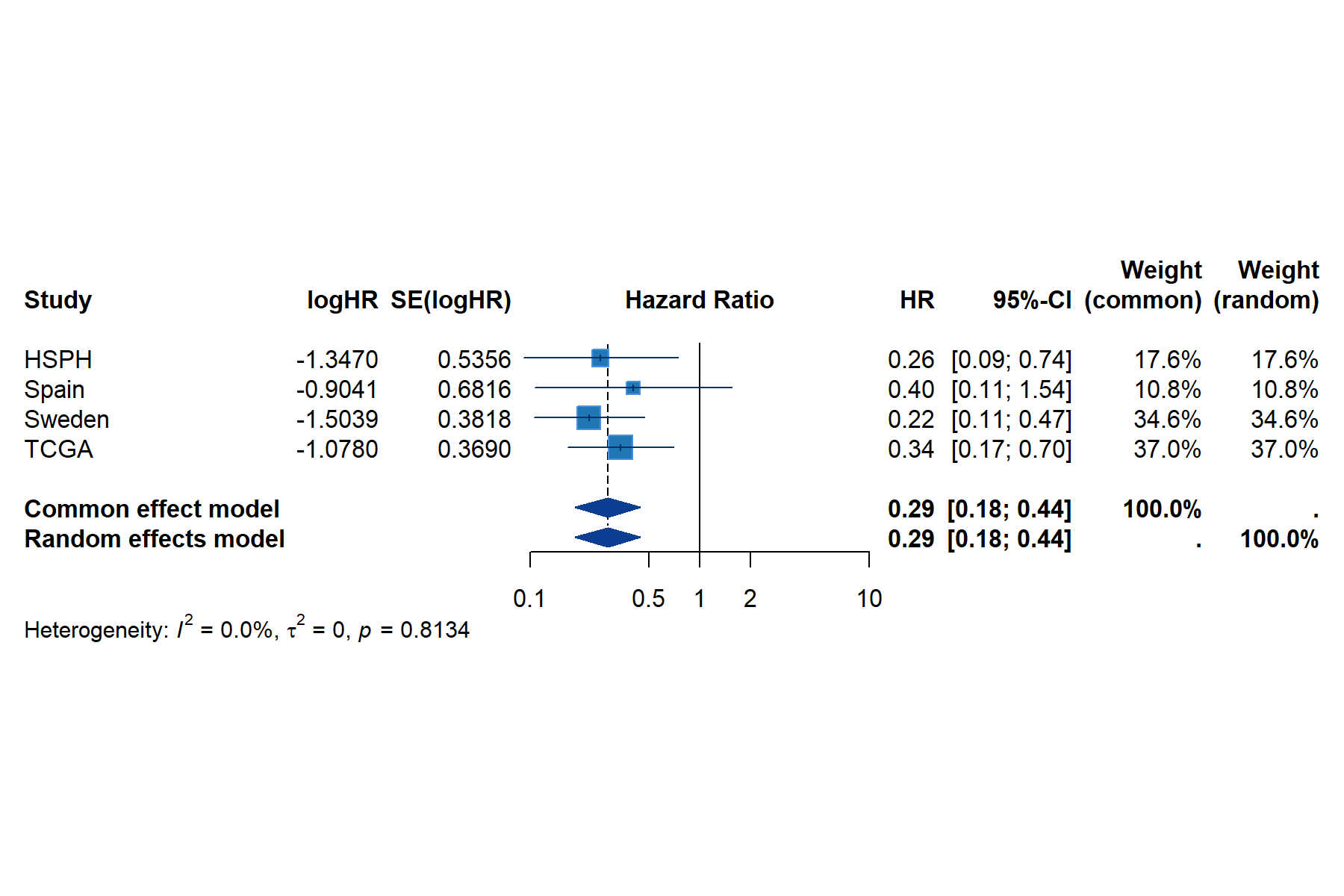


**Fig. S3．Distribution of cg12821679*_MAPRE3_* in LUAD and LUSC patients.** (A) Distribution plot of cg12821679*_MAPRE3_*. (B) Box plot of cg12821679*_MAPRE3_*. (C) Q-Q plot for association results of cg12821679*_MAPRE3_* in LUAD patients. (D) Q-Q plot for association results of cg12821679*_MAPRE3_* in LUSC patients. *MAPRE3*, Microtubule-associated protein RP/EB family member 3; LUSC, lung squamous cell carcinoma; LUAD, lung adenocarcinoma.

**
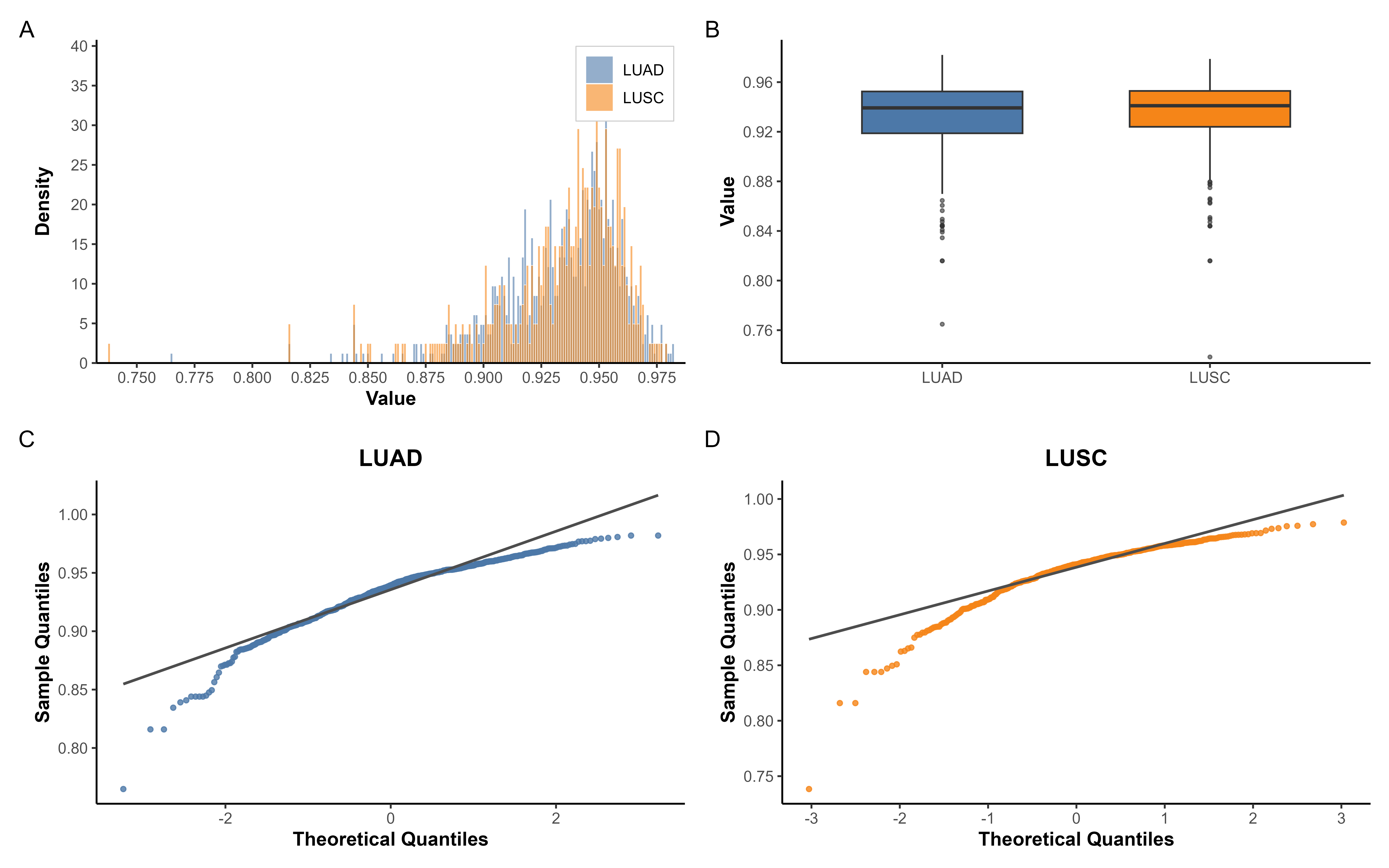
**
